# Control of dynamic cell behaviors during angiogenesis and anastomosis by Rasip1

**DOI:** 10.1242/dev.197509

**Published:** 2021-08-12

**Authors:** Minkyoung Lee, Charles Betz, Jianmin Yin, Ilkka Paatero, Niels Schellinx, Adam N. Carte, Christopher W. Wilson, Weilan Ye, Markus Affolter, Heinz-Georg Belting

**Affiliations:** 1 Department of Cell Biology, Biozentrum, University of Basel, Klingelbergstrasse 70, 4056 Basel, Switzerland; 2 Department of Molecular Biology, Genentech, Inc., South San Francisco, CA 94080, USA

**Keywords:** Zebrafish, Endothelial cells, Angiogenesis, Anastomosis, VE-cadherin, Rasip1

## Abstract

Organ morphogenesis is driven by a wealth of tightly orchestrated cellular behaviors, which ensure proper organ assembly and function. Many of these cell activities involve cell-cell interactions and remodeling of the F-actin cytoskeleton. Here, we analyze the requirement for Rasip1 (Ras-interacting protein 1), an endothelial-specific regulator of junctional dynamics, during blood vessel formation. Phenotype analysis of *rasip1* mutants in zebrafish embryos reveals distinct functions of Rasip1 during sprouting angiogenesis, anastomosis and lumen formation. During angiogenic sprouting, loss of Rasip1 causes cell pairing defects due to a destabilization of tricellular junctions, indicating that stable tricellular junctions are essential to maintain multicellular organization within the sprout. During anastomosis, Rasip1 is required to establish a stable apical membrane compartment; *rasip1* mutants display ectopic, reticulated junctions and the apical compartment is frequently collapsed. Loss of Ccm1 and Heg1 function mimics the junctional defects of *rasip1* mutants. Furthermore, downregulation of *ccm1* and *heg1* leads to a delocalization of Rasip1 at cell junctions, indicating that junctional tethering of Rasip1 is required for its function in junction formation and stabilization during sprouting angiogenesis.

## Abstract (precis)

**Summary:***In vivo* analysis of *rasip1* mutants reveals multiple roles for Rasip1 during angiogenic sprouting, anastomosis and lumen formation, including stabilization of tricellular junctions to permit coordinated cell rearrangements and multicellular tube formation.

## INTRODUCTION

The cardiovascular system is the first organ to become functional during embryonic development. The generation of vascular networks is essential for developmental patterning, growth and survival of the vertebrate embryo. As the embryo grows, the vasculature adjusts to the increasing demand of nutrients and oxygen by an expansion of the vasculature tree via sprouting angiogenesis, vascular remodeling and adaptation of blood vessel diameter. Vascular morphogenesis is driven by a wealth of dynamic cellular behaviors, which are regulated by molecular as well as physical cues, and are characterized by an extraordinary plasticity (Adams and Alitalo, 2007; Baeyens et al., 2016; Duran et al., 2017). At the cellular level, blood vessel morphogenesis and remodeling are accomplished by endothelial cell behaviors, including cell migration, cell rearrangement and cell shape changes (Betz et al., 2016). This repertoire of dynamic behaviors allows endothelial cells to respond rapidly to different contextual cues, for example during angiogenic sprouting, anastomosis, pruning, diapedesis or regeneration.

Previous studies have shown that vascular tube formation requires extensive and diverse cell shape changes and that these changes can be driven by junctional remodeling as well as dynamic regulation of the cortical actin cytoskeleton (Gebala et al., 2016; Paatero et al., 2018; Phng et al., 2015; Sauteur et al., 2014). Junctional remodeling is essential for cell rearrangements, which drive multicellular tube formation. Enlargement of the luminal space, by contrast, requires apical membrane invagination (Barry et al., 2016; Strilić et al., 2009). During anastomosis, the apical membrane can invaginate through the entire cell leading to the formation of a unicellular tube (Lenard et al., 2013).

Junctional remodeling and membrane invagination rely on dynamic regulation of the F-actin cytoskeleton at the endothelial cell junction and apical cortex, respectively. Small GTPases of the Rho family, including Cdc42, Rac1 and RhoA are essential regulators of F-actin dynamics and have been shown to play crucial roles during blood vessel formation *in vitro* and *in vivo* (reviewed by Barlow and Cleaver, 2019). In the vasculature, these GTPases are partially regulated by the adaptor protein Rasip1. Rasip1 has been shown to promote Rac1 and Cdc42 activity, whereas it inhibits RhoA activity by binding to the GTPase-activating protein Arhgap29 (Barry et al., 2016; Xu et al., 2011). Ablation of Rasip1 in mice and knockdown of *rasip1* in zebrafish cause severe vascular defects (Wilson et al., 2013; Xu et al., 2011). During vasculogenesis, Rasip1 is required for the lumenization of the dorsal aorta, in particular for clearing of apical membrane compartments from junctional proteins and for opening of the vascular lumen between endothelial cells (Barry et al., 2016). However, the role of Rasip1 during sprouting angiogenesis and anastomosis has not been studied in detail.

To gain more insight into the cellular and molecular mechanisms of vascular tube formation during angiogenesis, we have generated loss-of-function alleles in the zebrafish *rasip1* gene and performed high-resolution time-lapse imaging to observe junctional dynamics during sprouting angiogenesis and anastomosis. Loss of *rasip1* causes multiple vascular defects, with respect to angiogenic sprouting, including defects in cell proliferation, junctional stability and lumen formation. Furthermore, analyses of *radil-b* and *rasip1;radil-b* double mutants reveal partly redundant roles for the two proteins. Lastly, knockdown of *ccm1* and *heg1* phenocopies the apical junctional defects seen in *rasip1* mutants, suggesting a functional interaction between these proteins during blood vessel formation.

## RESULTS

### Loss of Rasip1 function causes broad vascular defects

To investigate the role of Rasip1 in vascular morphogenesis, we employed CRISPR/Cas9 technology to generate several mutant alleles, namely *rasip1^ubs23^*, *rasip1^ubs24^* and *rasip1^ubs28^*, respectively (Fig. S1). The *rasip1^ubs28^* allele comprises a deletion of about 35 kb, including the *rasip1* coding region from exon 3 to 16, resulting in a severely truncated protein lacking the Ras-association, Forkhead-association and Dilute domains (Fig. S1A). Because the truncated protein lacks all the conserved domains and cannot be detected by anti-Rasip immunostaining (Fig. S1F), we consider *rasip1^ubs28^* to be a null allele and focused our studies on the analysis of this mutant.

Homozygous *rasip1* mutants displayed hemorrhages and vascular instability in the cranial vasculature at 3 days of development (Fig. S1D). Furthermore, we observed transient pericardial edema between 3 and 5 days post-fertilization (dpf) (Fig. S1E), which usually disappeared during larval development. In the trunk, *rasip1* mutants showed reduced blood flow, which correlated with irregular and generally reduced diameter of the dorsal aorta (DA) and intersegmental vessels (ISVs) (Fig. S2A-C). These differences were transient; by 5 dpf, the average vessel diameter had recovered to normal size. Despite these severe defects, homozygous *rasip1* mutants recovered and ∼30% of them developed to become fertile adults.

### Reduced motility and proliferation during angiogenic sprouting in *rasip1* mutants

To find out whether the loss of *rasip1* affects dynamic cell behaviors, we performed time-lapse analyses, concentrating on the developing ISVs. ISV sprouts emerge from the DA at about 22-24 hours post-fertilization (hpf) and extend towards the dorsal side of the embryo. In wild-type embryos, they reach the dorsal part of the neural tube by 28 hpf and initiate formation of the dorsal longitudinal anastomotic vessel (DLAV) (Lawson and Weinstein, 2002). In our time-lapse movies, we observed that by 30 hpf almost all sprouts in the trunk region had completed dorsal sprouting and engaged in contact with neighboring sprouts (Fig. S3A,B; Movie 15). In contrast, ISV sprouting in *rasip1* mutants appeared sluggish and 25% of sprouts were incomplete by 30 hpf (Fig. S3A,B; Movie 16). To determine whether stunted outgrowth was reflected by a difference in the number of endothelial cells contributing to ISV sprouts, we counted nuclei within each sprout at 30 hpf (Fig. S3C). In wild-type and *rasip1* mutant embryos, the number of ISV nuclei was variable, ranging between one and five. However, we observed a clear enrichment of ISVs containing one or two nuclei in mutants compared with wild type, which contained three to four nuclei. Quantification of 5-ethynyl-2′-deoxyuridine incorporation in endothelial cells did reveal mild albeit non-significant proliferation defect in ISV and the DA (see peer review history file associated with this article). To determine whether the diminished cell number in ISV sprouts was caused either by reduced recruitment or by proliferation defects of endothelial cells, we tracked endothelial cell nuclei during ISV formation (Fig. S3D-G). In wild-type siblings, we observed that two cells migrated from the DA into the sprout, undergoing one round of division, each thus giving rise to an ISV consisting of four cells (Fig. S3E; Movie 17); ISVs comprising three cells were usually formed by two migrating cells and a single cell division (Fig. S3F; Movie 19). In *rasip1* mutants, we rarely observed cell divisions within the sprouts (Fig. S3D,G; Movies 18 and 20). Instead, most cells in the sprout originated from the DA, and occasionally we observed three cells migrating into the sprout (Fig. S3F; Movie 20). Hence, these results show that proliferation in sprouting endothelial cells is reduced in *rasip1* mutants and suggest that paucity of cell number may be compensated for partially by the recruitment of additional cells into the sprout.

### Instability of tricellular junctions inhibits formation of multicellular tubes

We next examined whether loss of *rasip1* affects angiogenic tube formation. In wild-type embryos, the multicellular architecture of ISVs tubes is established by concerted migratory and proliferative activities of endothelial cells. More specifically, the multicellular configuration is driven by junctional rearrangements along the vessel axis, which leads to effective cell pairing and, thus, multicellularity.

**Fig. 1.**
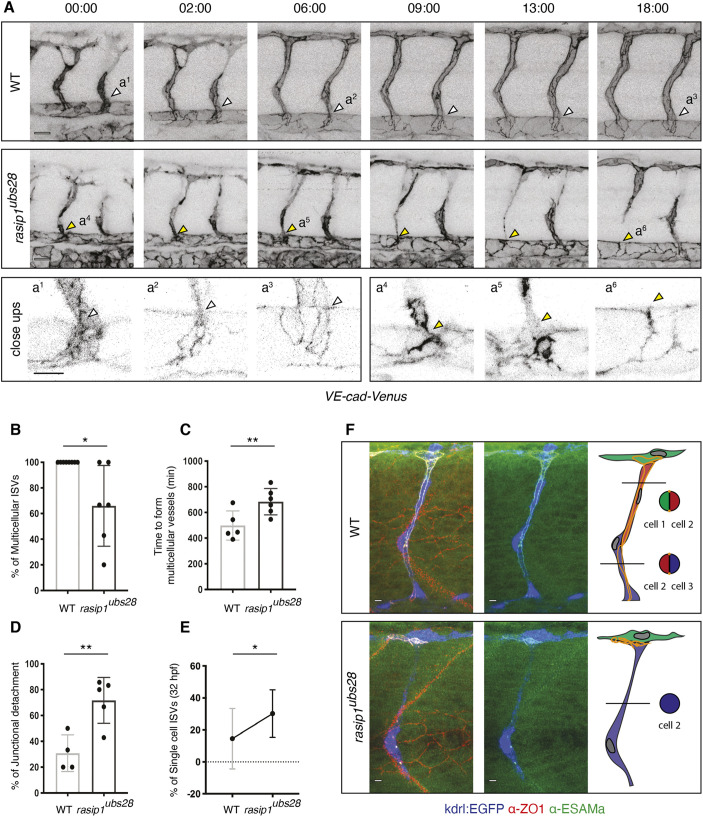
**Formation of multicellular vessels is impaired in *rasip1* mutants.** (A) Still images of time-lapse movies showing endothelial cell junctions (Cdh5-Venus) in wild-type (WT) and *rasip1^ubs28^* embryos (Movies 1 and 2). White arrowheads show maintained junctional contacts in wild-type ISV sprouts. Yellow arrowheads indicate junction detachment in mutant embryos. Bottom row shows close-ups (a1-a3: wild type; a4-a6: mutant) showing junctional detachment in a4 and a5. Scale bars: 20 μm. (B-E) Quantification of junctional and cellular configuration during ISV formation in wild-type and *rasip1^ubs28^* mutant embryos. (B) Percentage multicellular tubes at 48 hpf (WT *n*=8, mutant *n*=6). (C) Speed of multicellular tube formation (WT *n*=5, mutant *n*=6). (D) Percentage of ISVs per embryo showing junctional detachment (WT *n*=4, mutant *n*=5). (E) Percentage of single-cell ISVs at 32 hpf (WT *n*=8, mutant *n*=8). Quantifications were performed by counting ISVs showing the respective phenotypes, averaged by total ISVs analyzed per embryo. (F) Immunofluorescence staining of ZO-1 and Esama in *Tg(kdrl:EGFP)^s843^* at 32 hpf. Schematics on the right show the different cellular configurations of multicellular (WT) and unicellular (*rasip1* mutant) ISVs. Scale bars: 5 μm. The data were analyzed by unpaired two-tailed Mann–Whitney test (*P<0.1, **P<0.01); error bars indicate s.d.

As multicellular tubes have been characterized and can be recognized by continuous junctions along the blood vessel axis (Blum et al., 2008), we used a *VE-cad-Venus* reporter [*Tg(cdh5:cdh5-TFP-TENS-Venus)^uq11bh^*; Lagendijk et al., 2017] to follow the dynamics of endothelial junctions during ISV formation. In wild-type siblings, cell junctions elongated and spanned the entire extent of the ISV giving rise to multicellular tubes by 48 hpf (Fig. 1A). Notably, adherens junctions maintained the continuity of ISVs with the DA, where they formed vertices or tricellular junctions (Fig. 1A, white arrowheads; Movie 1). *rasip1* mutants showed a clear delay in multicellular tube formation (Fig. 1C) and at 48 hpf, on average, ∼40% of ISVs had not achieved a multicellular configuration (Fig. 1B). Moreover, time-lapse analysis of VE-cad-Venus showed defects in junctional development (Fig. 1A; Movie 2). Specifically, at the ventral base of the sprout, junctions that were normally tethered to the DA in wild-type embryos, lost this attachment and the junctional ring was ‘released’ in mutant embryos (Fig. 1A, yellow arrowheads,1D; Movie 2). This detachment resulted in one of the stalk cells moving up into the DLAV leaving a single cell spanning the distance between the DA and the DLAV (Fig. 1A,E,F). These results indicate that junctional interconnections at the base of the sprout are essential for cell intercalation to occur during multicellular tube formation. Thus, the loss of these connections in *rasip1* mutant prevents cell pairing and results in unicellular ISVs and, consequently, a defect in the cord-hollowing process underlying multicellular tube formation.

### Defects in junctional dynamics during blood vessel anastomosis

**Fig. 2.**
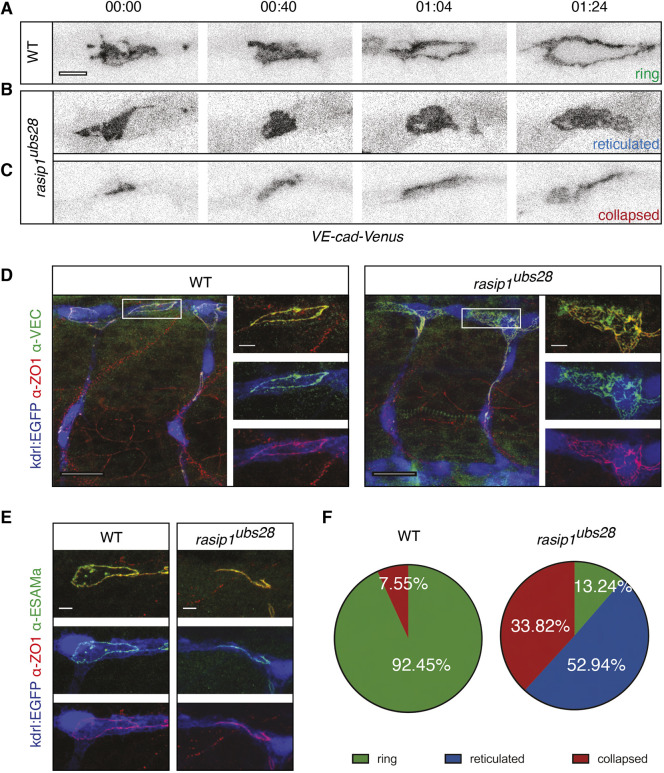
**Requirement of Rasip1 for dynamic re-localization of junctional proteins and junctional ring formation during anastomosis.** (A-C) Still images of time-lapse movies showing normal junctional patch-to-ring transformation in wild type (WT) (A; Movie 3) and aberrant ring formation in *rasip1^ubs28^* mutants (B,C; Movies 4 and 5). Transgenic embryos expressing a VE-cadherin-Venus fusion protein were imaged, starting at 30 hpf. Scale bar: 5 μm. (D) Immunofluorescence analysis of ZO-1 and VE-cadherin in *Tg(kdrl:EGFP)^s843^* at 32 hpf. *rasip1^ubs28^* mutant shows reticulated junctions between two cells in the DLAV; wild-type embryo forms a cleared apical compartment and a ring-shaped junction. Boxed areas indicate the regions shown at higher magnification to the right. Scale bars: 20 μm (main panels); 5 μm (insets). (E) Immunofluorescence analysis of ZO-1 and Esama in *Tg(kdrl:EGFP)^s843^* at 32 hpf showing a collapsed junction in the *rasip 1^ubs28^* mutant. Scale bars: 5 μm. (F) Quantification of observed junctional phenotypes at 32 hpf. *rasip1^ubs28^* mutants show a significant number of reticulated junctions and collapsed anastomotic rings compared with wild type (WT *n*=6 embryos, 53 analyzed rings; mutant *n*=8 embryos, 68 analyzed rings). *P*<0.0001 (χ^2^ test).

The growth and interconnection of vascular networks requires angiogenic sprouting as well as the interconnection of all sprouts by the process of anastomosis. At the cellular level, anastomosis occurs in a stereotyped fashion (reviewed by Betz et al., 2016). Neighboring sprouts initiate contact via tip cell filopodia and form a junctional ring, which surrounds apically polarized membrane. Junctional ring and apical membrane formation in both of the contacting cells leads to the formation of a luminal pocket, which is later connected to the lumen of the nascent vessel. The process of anastomosis in zebrafish serves as a paradigm to study the cell biology of blood vessel formation and includes processes such as apical polarization, junctional rearrangements and lumen formation, which occur within a 4-6 h window (Herwig et al., 2011). To assess the role of Rasip1 during anastomosis, we compared the junctional dynamics in wild type and *rasip1* mutants using VE-cad-Venus (Fig. 2A-C; Movies 3-5) and Pecam-EGFP (Fig. S4; Movies 21 and 22) as junctional reporters. Time-lapse analyses revealed two different defects in *rasip1* mutants during junctional ring formation. In ∼53% of anastomosis events (Fig. 2A,D,F), we observed ectopic accumulation of VE-cadherin-Venus or Pecam-EGFP (Fig. S4) within the junctional ring, revealing a defect in relocating these junctional proteins from the apical compartment to cell junctions. Alternatively, in ∼34% of cases, the anastomotic ring (Fig. 2C,E,F) elongated along the blood vessel axis but failed to maintain a lateral axis, leading to a collapsed junctional ring.

The described defects were confirmed by immunofluorescent analysis of the junctional proteins VE-cadherin (VE-cad; Cdh5), ZO-1 (tight junction protein 1, Tjp1) and Esama (endothelial-selective adhesion molecule a) (Fig. 2D,E). In *rasip1* mutants, VE-cad, ZO-1 and Esama colocalized and formed reticulated junctions within the apical compartment. Together, these observations indicate that Rasip1 plays a crucial role in the dynamic re-localization of junctional components during *de novo* junction and lumen formation.

### Autonomous requirement of *rasip1* during sprouting angiogenesis and anastomosis

**Fig. 3.**
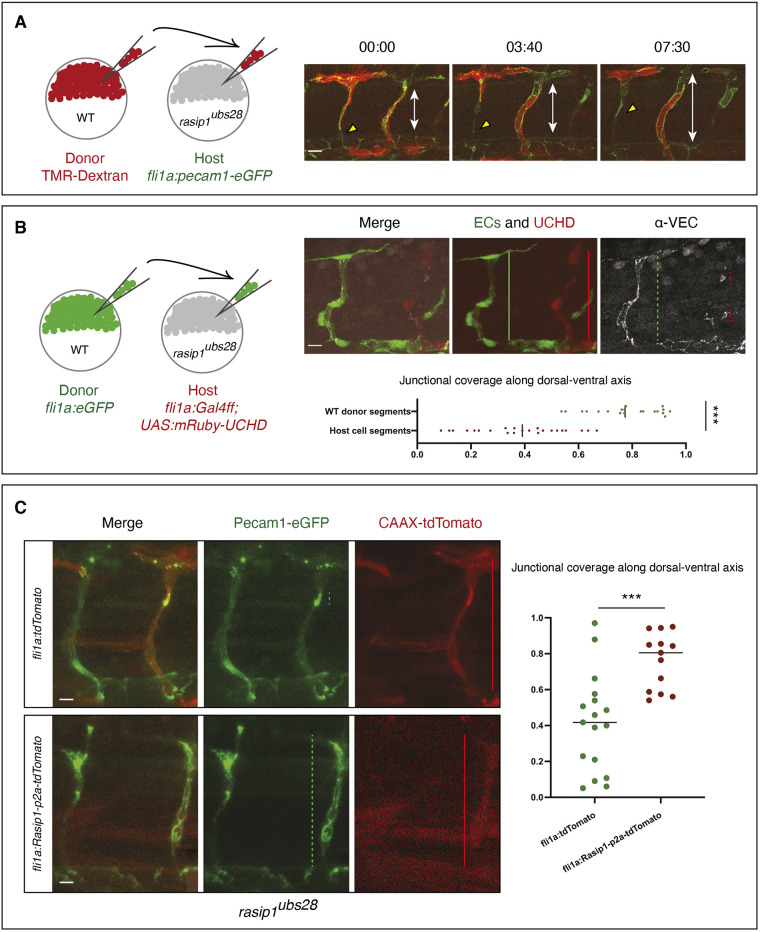
**Autonomous requirement of Rasip1 during ISV formation.** (A) Schematic showing transplantation of TMR-labeled donor wild-type (WT) cells into *rasip1^ubs28^* mutant hosts with both donor and host cells expressing Pecam1-EGFP in endothelial cells. Time-lapse images of wild-type cells (red) in *rasip1^ubs28^* mutant hosts showing that wild-type cells elongate and maintain junctional contacts (Movie 6). Double-headed arrows indicate the extent of the ISV between DA and DLAV. (B) Schematic showing transplantation of EGFP-labeled wild-type donor cells [*Tg(flia:EGFP)^y1^*] into *rasip1* mutant host embryos [*Tg(flia:Galff)^ubs3^; (UAS:UCHD-mRuby2)^ubs20^*]. Immunofluorescence analysis of VE-cadherin in embryos at 32 hpf. Transplanted wild-type cells elongate and form multicellular tubes. Graph shows quantification of junctional coverage along the dorsal-ventral axis (ratio between the accumulated length of junctions to the length of the ISV) (analyzed WT donor segment *n*=20, host cell segment *n*=23). (C) Rescue by transient endothelial-specific *rasip1* expression. Endothelial expression was achieved by DNA microinjection using a *fli1a:Rasip1-p2a-tdTomato-CAAX* construct or *fli1a:tdTomato-CAAX* as a control. Graph shows quantification of junctional coverage along the dorsal-ventral axis (analyzed ISVs in *rasip1^ubs28^* mutant background *n*=17, *rasip1* over-expression *n*=13). Scale bars: 20 μm. Analyzed by unpaired two-tailed Mann–Whitney test (***P<0.0001 in B, ***P<0.001 in C); error bars indicate s.d.

The diverse vascular defects in *rasip1* mutants prompt the question of whether mutant phenotypes may arise as secondary defects. To address this question, we performed transplantation experiments to generate genetic mosaic embryos and tested whether single or small groups of wild-type endothelial cells behave normally in a *rasip1* mutant background (Fig. 3). We performed two types of experiments. First, we used *Tg(fli1a:pecam1-EGFP)^ncv27^* and transplanted TMR-labeled wild-type donor cells into *rasip1* mutant hosts (Fig. 3A). Time-lapse analysis showed that transplanted wild-type endothelial cells maintained junctional contacts with the DA, and were able to elongate their junctions within the sprouts and form a multicellular tube in the *rasip1* mutant background (Fig. 3A; Movie 6). For a quantitative analysis of ISV morphology, we transplanted wild-type [*Tg(fli1a:EGFP)^y1^*] donor cells into *rasip1* mutant hosts expressing mRuby-UCHD [*Tg(fli1a:Gff)^ubs3^*;*(UAS:mRuby-UCHD)^ubs20^*] and analyzed junctional patterns by anti-VE-cadherin immunofluorescent staining (Fig. 3B). In ISVs made up by host cells, we found discontinuous junctional patterns consistent with the cell-pairing defects observed in *rasip1* mutants. In contrast, donor-derived wild-type cells were able to rearrange and form a multicellular tube.

To test, whether expression of *rasip1* in endothelial cells is sufficient to rescue the *rasip1* mutant phenotype, we performed rescue experiments by transient *rasip1* expression and assayed for junctional coverage as in the previous experiments (Fig. 3C). Injection of the *fli1a:Rasip1-p2a-tdTomato-CAAX* rescue construct into *rasip1* mutant embryos led to mosaic expression of tdTomato in endothelial cells. In contrast to control embryos, which expressed only the fluorescent reporter, expression of *rasip1* resulted in efficient rescue of cell rearrangement in forming ISV, illustrated by increased junctional coverage along the vessel. In summary, these transplantation and overexpression studies indicate an autonomous requirement for Rasip1 in the control of junctional dynamics during angiogenesis.

### *rasip1* mutants display transient intracellular luminal pockets

**Fig. 4.**
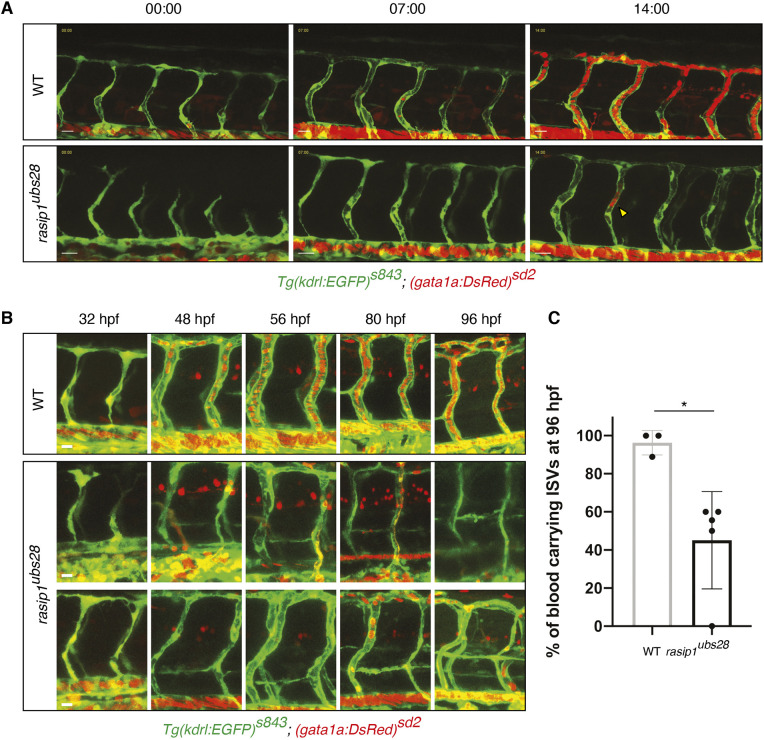
**Protracted delays in lumen formation in *rasip1* mutants.** (A,B) Live images of *Tg(kdrl:EGFP)^s843^; (gata1a:DsRed)^sd2^* embryos. (A) Still images of time-lapse movies starting at 30 hpf (Movies 7 and 8). (B) Tracking of individual unlumenized ISV during embryonic development (32 to 96 hpf). Scale bars: 20 μm. (C) Percentage of blood-carrying ISVs at 96 hpf (WT *n*=3 embryos, 28 analyzed ISVs, mutant *n*=5, 46). Analyzed by unpaired two-tailed Mann–Whitney test (*P<0.1); error bars indicate s.d.

**Fig. 5.**
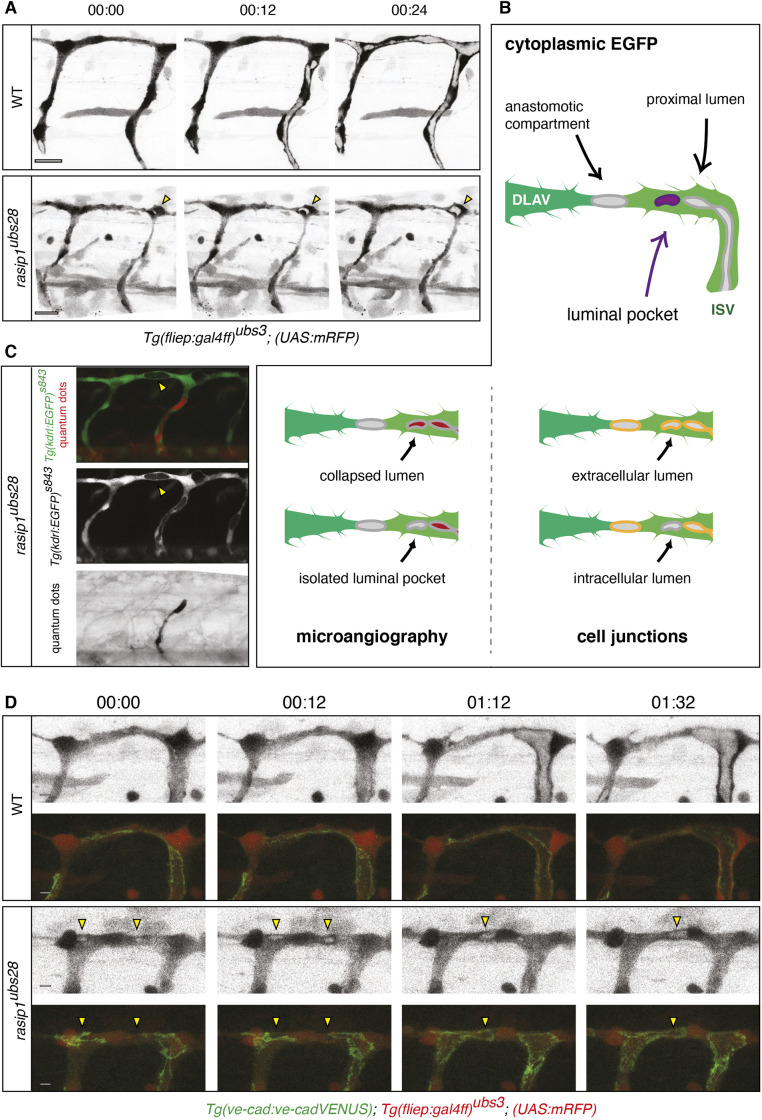
**Analysis of ectopic luminal pockets during DLAV formation in *rasip1* mutants.** (A) Still images of time-lapse movies showing the emergence of ectopic luminal pockets (yellow arrowheads) in *rasip1* mutants (Movies 9 and 10). (B) Schematic of possible cellular localizations of ectopic lumens. To differentiate between these possibilities, two types of experiments were performed: microangiography (C) and colocalization of luminal pockets with junctional marker (D). (C) Visualization of ectopic lumens and patent lumens in a *rasip1^ubs28^* embryo (36 hpf). Ectopic luminal pockets are indirectly visualized by the absence of cytoplasmic EGFP (yellow arrowheads) [*Tg(kdrl:EGFP)^s843^*]. The patent lumen is marked by microangiography using quantum dots in red (black in bottom panel). Ectopic lumens are not part of the patent vasculature. (D) Still images of time-lapse movies during lumen formation in the DLAV from around 32 hpf onward in wild-type (WT; top) and *rasip1^ubs28^* (bottom) embryos (Movies 11-14). Endothelial cells are labeled with mRFP (grayscale images) and junctions are labeled by VE-cad-Venus (merged images). Yellow arrowheads indicate the ectopic luminal pockets in the *rasip1* mutant. Scale bars: 5 μm.

As shown above, *rasip1* mutant embryos display reduced vessel diameter and luminal defects (Fig. S2A-C). These luminal defects affect the onset of blood flow in the ISVs (Fig. 4A, yellow arrowhead; Movies 7 and 8). We observed that initially unlumenized ISVs remained unlumenized at least until day 4 of development (96 hpf) (Fig. 4B). Moreover, in some instances we observed that initially lumenized blood-carrying ISVs collapsed in subsequent stages (up to 96 hpf), indicating a role for Rasip1 in lumen maintenance (Fig. 4B,C). Thus, although luminal defects can be attributed to the inability of endothelial cells to rearrange into a multicellular configuration (Figs 2 and 3), the above observations suggest an additional defect in the formation or maintenance of a continuous luminal compartment. This notion is supported by time-lapse analysis of ISV and DLAV formation during lumen formation (Fig. 5A). The timing of lumen formation in the ISV is variable but usually starts between 30 and 32 hpf. In wild-type embryos, we observed that, upon initiation, continuous lumens were formed within 30 min (Fig. 5A; Movies 9 and 10). In *rasip1* mutants, lumen formation was delayed and discontinuous. Instead, we often observed luminal pockets in the dorsal aspects of the ISV (Fig. 5A, yellow arrowheads; Movie 10; Fig. S6D). We surmised that luminal pockets could arise in three different ways (Fig. 5B): (1) by a collapse of a previously patent tube, (2) by a local cord hollowing event, which failed to interconnect with other luminal pockets, or (3) by the formation of large intracellular vacuolar structures, which failed to fuse with luminal membrane.

To differentiate between these scenarios, we performed a series of experiments. To test whether luminal pockets arise by lumen collapse, we performed microangiography using a fluorescent tracer dye in 28-30 hpf embryos (Fig. 5C). Upon intravascular injection, the entire patent vasculature was labeled by quantum dots. In *rasip1* mutants, however, we observed that, although the base of the ISV was positive for quantum dots, local luminal pockets in the DLAV were negative (Fig. 5C, yellow arrowheads). This strongly argues that luminal pockets in *rasip1* mutants arise locally and do not represent luminal remainders formed by lumen collapse.

We next wanted to check whether luminal pockets represented intra- or extracellular compartments. Extracellular luminal compartments arise between cells in a process called cord hollowing, whereas intracellular lumens are thought to form by vacuolation (Davis et al., 2011). During anastomosis in zebrafish embryos, cord hollowing generates luminal pockets, which form as transient structures at the interface between contacting tip cells (Blum et al., 2008). This interface is formed by a ring-shaped junction, which surrounds an apical compartment of both tip cells (Herwig et al., 2011). Thus, these extracellular pockets are demarcated by junctional rings, whereas intracellular pockets should be outside of these rings. To test these possibilities, we examined lumen formation in *rasip1* mutants expressing VE-cad-Venus and found that they were, in contrast to wild type, located outside the junctional ring (Fig. 5D, 00:12; Movies 11-14), appearing as vesicular structures within the endothelial cytoplasm. Later on (Fig. 5D, 01:32), these intracellular lumens were incorporated into the area covered by the junctional ring, therefore representing transient structures. Taken together, these findings show that loss of Rasip1 function leads to a transient accumulation of intracellular vesicles, which subsequently merge into the anastomotic compartment, suggesting that Rasip1 may be required for normal vesicle transport or vesicle fusion during the cord-hollowing process, which occurs during anastomosis.

### Rasip1 localizes to apical membranes and endothelial cell junctions

**Fig. 6.**
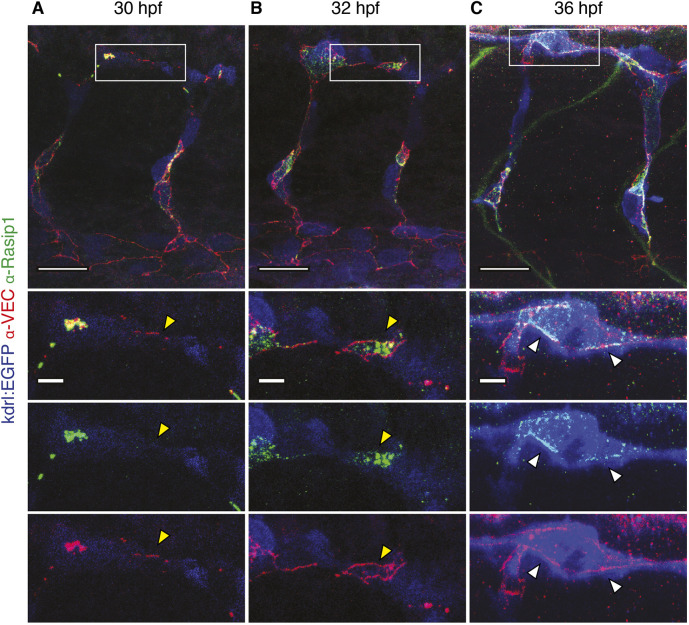
**Apical-to-junctional re-localization of Rasip1 during blood vessel fusion.** (A-C) Immunofluorescence labeling of Rasip1 and VE-cadherin during different stages of DLAV formation (30-36 hpf). At 30 and 32 hpf Rasip1 does not localize to endothelial junctions (yellow arrowheads). At 32 hpf, Rasip1 is restricted to the apical surface of the anastomotic ring (yellow arrowheads). At 36 hpf, Rasip1 localizes to endothelial junctions (VE-cadherin, white arrowheads). Boxed areas indicate the regions shown at higher magnification below. Scale bars: 20 μm (top panels); 5 μm (insets).

Our mutant analyses indicate a requirement for Rasip1 in junction formation and remodeling, as well as in lumen formation and maintenance. To gain a better understanding of how Rasip1 may be involved in these processes, we generated an antibody against zebrafish Rasip1 to discern the subcellular localization of the protein during angiogenesis. Immunofluorescence analysis confirmed endothelial expression of Rasip1 in zebrafish embryos (Fig. S5). Notably, Rasip1 protein levels appeared to be dynamically regulated. During vasculogenesis, until the emergence of intersegmental sprouts, Rasip1 was detected at high levels in the dorsal aorta (Fig. S5A,B, yellow arrowheads). In contrast, Rasip1 protein was highly expressed in sprouting ISVs whereas it became downregulated in the dorsal aorta (Fig. S5C,D, yellow bars), supporting the notion that Rasip1 primarily functions during blood vessel morphogenesis rather than during vessel maintenance. High-resolution imaging revealed specific subcellular localization during blood vessel formation. In the context of anastomosis, three different phases could be discerned. First, during contact formation (30 hpf), we found that Rasip1 is absent from newly formed contacts (Fig. 6A, yellow arrowheads). However, Rasip1 was observed colocalizing with large junctional patches prior to discernable formation of apical compartments. During later stages of anastomosis, when the anastomotic ring had formed, Rasip1 was restricted to the apical compartment within the junctional ring with no detectable Rasip1 at the junction (Fig. 6B, yellow arrowheads). However, shortly later – during the establishment of the DLAV (36 hpf) and subsequently after full development of angiogenic vessels (48 hpf) – we found that Rasip1 also localized to endothelial cell junctions (Fig. 6C; Fig. S5E, white arrowheads). Taken together, these studies show that Rasip1 is dynamically distributed during different phases of blood vessel formation. In particular, the dynamic subcellular distribution to apical membrane compartments and endothelial cell junctions suggests a sequential requirement for Rasip1 during apical compartment formation and junctional remodeling, respectively.

Because of the early localization of Rasip1 to the apical membrane, we wanted to test whether loss of Rasip1 function affects apical polarization during blood vessel formation. To this end, we generated a transgenic reporter [*Tg(EGFP-podxl)^ubs29^*], which labels the apical membrane compartment (Fig. S6E). At 48 hpf, we observed normal luminal localization of EGFP-Podxl in *rasip1* mutants (Fig. S6E). In ISVs that displayed luminal defects, we observed slightly irregular distribution of EGFP-Podxl in affected areas (insets in Fig. S6E). These observations suggest that, in spite of its apical localization, Rasip1 is not required for apical polarization in endothelial cells.

### Overlapping requirement of Rasip1 and Radil-b in blood vessel formation

Rasip1 protein has been shown to be an effector protein of the small GTPase Rap1 (Gingras et al., 2016). Protein-binding studies have shown that Rasip1 can form multimeric complexes consisting of Rap1, Rasip1, Radil (Ras-associating-dilute-domain) and the GTPase-activating protein Arhgap29 (Post et al., 2013). Furthermore, it has been shown that the core of these complexes can be formed by a Rasip1 homodimer or a Rasip1/Radil heterodimer (de Kreuk et al., 2016), indicating partly overlapping functions of these proteins in endothelial cells. Rasip1 and Radil are closely related proteins (Fig. S6A) sharing several protein interaction domains, such as a Ras association (RA) domain conferring binding to Rap1, and a forkhead association (FHA) domain binding the transmembrane receptor Heg1. An additional PDZ domain is unique to Radil and is thought to interact with the GTPase-activating protein Arhgap29 (Post et al., 2013). Radil function in endothelial cells has, so far, only been addressed in cell culture experiments, which established its above-mentioned protein interactions and indicated a role of Radil in endothelial barrier maintenance and the regulation of endothelial cell adhesion (de Kreuk et al., 2016; Pannekoek et al., 2014). Thus, we wanted to determine the role of Radil during blood vessel morphogenesis *in vivo* and compare its requirement with that of Rasip1. The zebrafish genome contains three *radil* paralogs: *radil-a*, *-b* and -*c* (Fig. S6A). We analyzed blood vessel formation in *radil-b^sa20161^* mutants, which carry a nonsense mutation (Tyr129→STOP) near the N terminus of the protein (Fig. S6B).

**Fig. 7.**
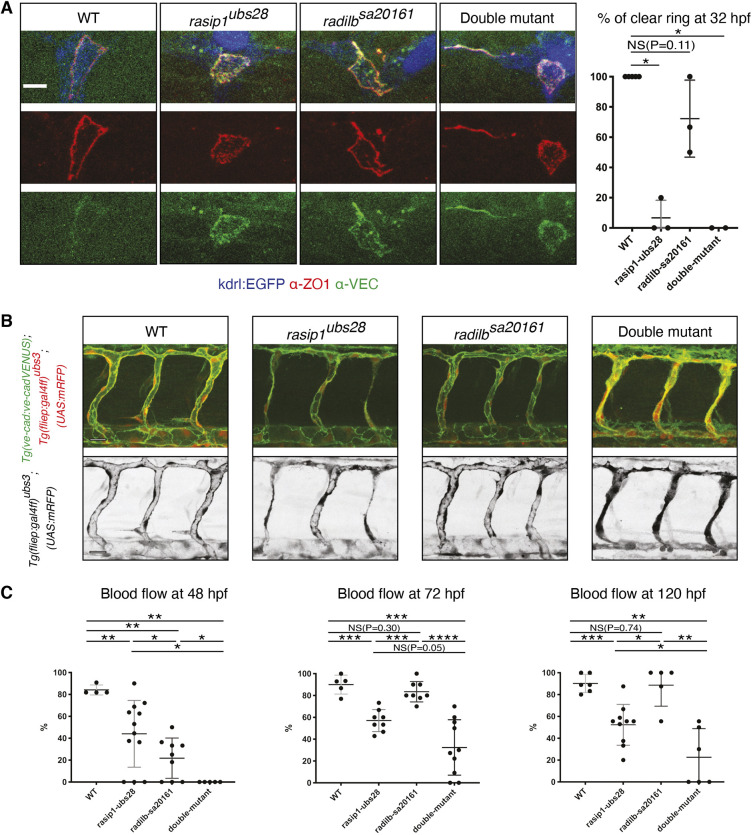
**Phenotypic comparison *of radil-b* single and of *rasip1/radil-b* double mutants suggests partially overlapping functions during vascular morphogenesis.** (A) Immunofluorescence analysis of ZO-1 and VE-cadherin distribution in *Tg(kdrl:EGFP)^s843^* at 32 hpf. The graph shows the percentage of DLAVs exhibiting a clear ring at 32 hpf. Number of embryos and junctions analyzed at 32 hpf: WT (5, 15), *rasip1^ubs28^* (3, 11), *radil-b^sa20161^* (3, 6) and double mutant (2, 3). (B) Live images at 48 hpf using *Tg(ve-cad:ve-cadVENUS); Tg(fliep:gal4ff)^ubs3^; (UAS:mRFP)* reporter lines. Scale bars: 20 µm. (C) Quantification of blood flow defects in ISVs at 48, 72 and 120 hpf in *rasip1^ubs28^*, *radil-b^sa20161^* and in double mutants. *radil-b^sa20161^* mutants show only transient defects in blood flow at 48 hpf. *rasip1^ubs28^*; *radil-b^sa20161^* double mutants show a strongly enhanced phenotype. Number of embryos and ISVs analyzed at 48, 72 and 120 hpf, respectively: WT (4, 44; 5, 71; 6, 87), *rasip1^ubs28^* (12, 176; 8, 119; 10, 118), *radil-b^sa20161^* (9, 105; 8, 78; 5, 41) and double mutant (5, 56; 10, 102; 6, 55). Analyzed by unpaired two-tailed Mann–Whitney test (A,C) (NS, no significance; *P<0.1, **P<0.01, ***P<0.001, ****P<0.0001); error bars indicate s.d.

*radil-b* mutants were homozygous viable and could be raised to fertile adulthood. Nevertheless, they exhibited several vascular defects similar to *rasip1* mutants, including cerebral hemorrhages (Fig. S6C), isolated luminal pockets (Fig. S6D) and a reduction of blood flow (Fig. 7C), supporting the notion that both proteins are involved in the same molecular pathways. In contrast, *radil-b* mutants did not phenocopy the junctional re-localization defect observed in *rasip1* mutants during anastomosis (Fig. 7A), suggesting that the proteins may also have unique functions. *radil-b* mutants generally exhibited milder defects, in particular with respect to cellular architecture compared with single *rasip1* mutants and *rasip1;radil-b* double mutants (Fig. 7B). Furthermore, *rasip1;radil-b* double mutants showed stronger sprouting and lumen formation defects than either single mutant (Fig. 7; Fig. S6E), suggesting that, although both proteins are required in this process, they likely act in a partially redundant manner.

### Knockdown of *ccm1* and *heg1* phenocopies aspects of the *rasip1* mutant

The Rasip1/Radil/Rap1 complex can bind via the FHA domain to the orphan transmembrane receptor Heg1 (Gingras et al., 2016). This interaction has been shown to tether Rasip1 to endothelial cell junctions (Post et al., 2013). To address the relevance of this interaction during ISV morphogenesis, we analyzed *heg1* morphants in order to test whether loss of Heg1 function showed any *rasip1*-like vascular phenotypes. We also examined the phenotypes of *ccm1* morphants. Ccm1 binds to Heg1 independently of Rasip1 (Gingras et al., 2012), and thus might indirectly influence Rasip1 function.

**Fig. 8.**
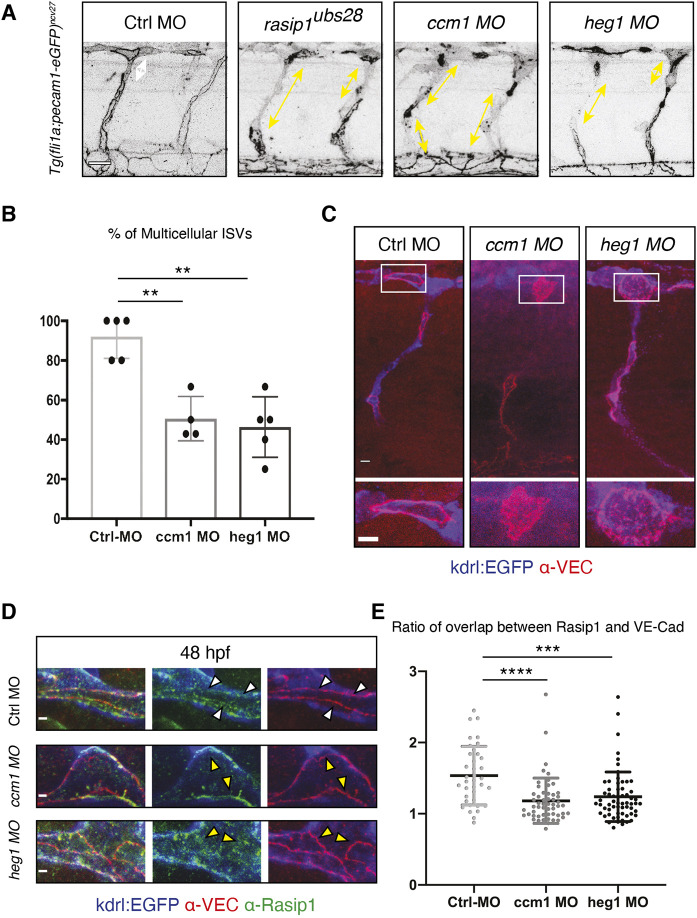
**Loss of Ccm1 and Heg1 phenocopies aspects of *rasip1* mutants.** (A) Defects in cell rearrangements induced by loss of *rasip1*, *ccm1* and *heg1* function. Live images at 48 hpf using the *Tg(fli1a:Pecam-EGFP)^ncv27^* reporter line. White and yellow arrows show the gaps between junctions. Scale bar: 20 µm. (B) Quantification of multicellular ISVs at 48 hpf [control (Ctrl) morpholino (MO)-injected embryos *n*=5, 24 analyzed ISVs; *ccm1* MO *n*=4, 26; *heg1* MO *n*=5, 23]. (C) Immunofluorescence analysis of control, *ccm1* and *heg1* morphants at 32 hpf. Transgenic *Tg(kdrl:EGFP)^s843^* embryos were stained for VE-cadherin. Boxed areas indicate the regions shown at higher magnification below. Scale bars: 5 μm. (D) Immunofluorescence analysis of control, *ccm1* and *heg1* morphants using anti-VE-cadherin and anti-Rasip1 antibodies at 48 hpf. Scale bars: 2 µm. White arrowheads indicate colocalization of VE-cadherin and Rasip1. Yellow arrowheads indicate absence of Rasip1 from endothelial cell junctions. (E) Quantification of the relative intensity of Rasip1 localized at junctions compared with that on the apical membrane. Ratio=junctional Rasip1/apical Rasip1 referenced by VE-Cadherin (junction). Control-MO injected embryos display elevated junctional Rasip1 compared with the *ccm1* and *heg1* morphants (analyzed regions in control MO *n*=37; *ccm1* MO *n*=54; *heg1* MO *n*=62). Analyzed by unpaired two-tailed Mann–Whitney test (**P<0.01, ***P<0.001, ****P<0.0001); error bars indicate s.d.

Consistent with previous studies, *ccm1* and *heg1* morphants lacked blood flow and exhibited blood vessel and heart dilations (Hogan et al., 2008; Kleaveland et al., 2009; Stainier et al., 1996). We consistently observed hemorrhages, which were less pronounced than in *rasip1* mutants (Fig. S7A). However, similar to *rasip1* mutants, ISV sprouting and development appeared to be delayed (Fig. S7B-D). To assess the role of Heg1 and Ccm1 in junctional rearrangements, we imaged the dynamics of Pecam1-EGFP fusion proteins during ISV formation (Fig. 8A,B). Here, we observed detachment of junctions and an inhibition of cell rearrangements (Fig. 8A), which resulted in a failure in the formation of multicellular tubes (Fig. 8B). In addition, it appeared that Pecam-EGFP was not entirely cleared from apical compartments during the formation of the DLAV. To verify these observations, we performed immunofluorescence analyses to determine whether endogenous VE-cadherin protein was cleared from the apical membrane compartment in *heg1* and *ccm1* morphants. In both conditions, VE-cadherin accumulated in the apical compartment embedded within the junctional rings, similar to observations in *rasip1* mutants (Fig. 8C).

The above findings show that *ccm1* and *heg1* loss of function phenocopy specific defects of *rasip1* mutants during crucial processes of blood vessel formation. Because of the established Rasip1/Heg1 interaction, we investigated whether these defects may be caused by Rasip1 delocalization. To this end, we performed immunofluorescence analyses and compared the relative Rasip1 levels in endothelial cell junctions (marked by VE-cadherin) and the neighboring apical membrane (Fig. 8D,E). Whereas control embryos showed an average junction-to-apical membrane ratio of about 1.5, this ratio was significantly reduced in both *ccm1* and *heg1* morphants, indicating impaired recruitment of Rasip1 to the junction (Fig. 8E). Together, these findings support the notion that junctional localization of Rasip1 is essential for junctional remodeling during angiogenic sprouting and anastomosis and that this Rasip1 localization requires Heg1 as well as Ccm1.

## DISCUSSION

Small GTPases of the Rho family play a key role in the regulation of cellular activities during blood vessel formation. For example, they serve as molecular switches to control cytoskeletal dynamics, cell adhesion and junction assembly during angiogenic sprouting and lumen formation (Barlow and Cleaver, 2019). Rasip1 has been described as an effector protein of small GTPase signaling during blood vessel formation and maintenance (Koo et al., 2016; Wilson et al., 2013; Xu et al., 2011). Rasip1 protein contains multiple protein-binding domains and has been shown to interact directly with its paralog Radil, the small GTPase Rap1 and the transmembrane protein Heg1 (de Kreuk et al., 2016; Gingras et al., 2016; Wilson et al., 2013). By further association with proteins such as Arhgap29 and Ccm1, these proteins control cortical actomyosin tension and endothelial junction formation and dynamics (Post et al., 2015; reviewed by Lampugnani et al., 2017; Wilson and Ye, 2014). Analyses of mutant mouse embryos have shown that Rasip1 is required for proper lumen formation and maintenance in blood and lymphatic vessels (Koo et al., 2016; Liu et al., 2018; Wilson et al., 2013). During vasculogenesis, Rasip1 is required for the establishment of the nascent apical compartment and subsequently for lumen expansion, presumably mediated by regulation of Cdc42 and RhoA, respectively (Barry et al., 2016).

### Multiple vascular defects in zebrafish *rasip1* mutants

In order to gain a better understanding of how morphogenetic cell behaviors are controlled by Rasip1, we generated loss-of-function mutants in the zebrafish *rasip1* gene and analyzed these mutants focusing on cellular and junctional dynamics during angiogenic sprouting and anastomosis. Overall, our findings are in agreement with previously published vascular defects in mouse development, but also provide insights into the regulation of junctional dynamics during angiogenic sprouting and lumen formation.

*rasip1* mutants display numerous vascular defects, including cranial hemorrhage, reduced blood circulation and reduced diameter of the dorsal aorta, consistent with previously published *rasip1* knockdown experiments in zebrafish (Wilson et al., 2013). Furthermore, we observe delayed angiogenic sprouting, as well as abnormalities in lumen formation and impaired cell rearrangements, junctional dynamics and stability. Whereas the wide range of mutant phenotypes may suggest that some defects may arise secondarily, our transplantation and rescue experiments show that endothelial expression of *rasip1* is sufficient to regulate junctional dynamics cell-autonomously during angiogenic sprouting and anastomosis. The phenotypes of *rasip1* mutants are consistent with defects in the control of F-actin and junctional dynamics. These phenotypes are, however, distinct from those seen in *ve-cad* (*cdh5*) mutants (Sauteur et al., 2014), indicating that Rasip1 regulates endothelial activities beyond cell junctions.

### Dynamic regulation of Rasip1 expression and subcellular localization

Endothelial-specific expression of Rasip1 has been reported in several vertebrate species, including mouse, *Xenopus* and zebrafish, and Rasip1 appears to be expressed in the entire embryonic vasculature (Wilson et al., 2013; Xu et al., 2009). In contrast to its broad endothelial transcription, the distribution of Rasip1 protein appears to highly regulated, with respect to the overall level and its subcellular localization. During vasculogenesis, Rasip1 is readily detected at high levels in the dorsal aorta, whereas expression is somewhat reduced at later stages. This downregulation of Rasip1 in the dorsal aorta coincides with the commencement of blood flow, suggesting that Rasip1 protein levels and localization may be controlled by shear stress. The downregulation of Rasip1 protein in more mature vessels suggests an essential role during blood vessel morphogenesis. In agreement with this, Rasip1 does not appear to be required in established blood or lymphatic vessels (Koo et al., 2016; Liu et al., 2018).

During ISV sprouting, we observe a shift in Rasip1 distribution from the apical membrane compartment during the early stages to higher levels at cell junctions during the later stages of vascular tube formation. The early apical localization of Rasip1 is in agreement with a previously proposed role in early apical-basal polarization, likely upstream of Cdc42 (Barry et al., 2016). It will be important to determine how this differential localization is accomplished or to what extent it reflects different functions of Rasip1 during tubulogenesis. In *Drosophila*, it has been shown that apical localization of Canoe (the ortholog of vertebrate *afadin* and a homolog of *rasip1*) is dependent on Rap1 (Bonello et al., 2018). Therefore, Rap1 is a putative candidate for localization of Rasip1 to the apical membrane during vertebrate angiogenesis.

### A role for Rasip1 in angiogenic sprouting and blood vessel assembly

Sprouting angiogenesis is accomplished by concerted endothelial cell dynamics, including cell migration, rearrangement, elongation and proliferation, all of which are affected by the loss Rasip1 function. *rasip1* mutant ISVs contain fewer cells than do wild-type ISVs, which reflects a reduced rate of cell proliferation during sprouting.

Furthermore, we observe a frequent failure of *rasip1* mutant ISVs to form multicellular tubes. Formation of multicellular ISVs does not depend of the number of cells present in the sprout (Angulo-Urarte et al., 2018), but rather relies on cell rearrangements driven by junctional remodeling (Sauteur et al., 2014). For example, loss of VE-cad prevents junction elongation and renders endothelial cells unable to move over each other and effectively pair to form a multicellular tube (Paatero et al., 2018; Sauteur et al., 2014). In *rasip1* mutants, however, the defect in cell pairing is caused by junctional detachments. When we imaged VE-cad dynamics during ISV sprouting in wild-type embryos, we found that, in many cases, two stalk cells maintain contact with the dorsal aorta. In *rasip1* mutant sprouts, we observed that one of these cells detached from the dorsal aorta and ‘retracted’ to the dorsal part of the ISV, leaving the remaining cell unpaired. Stalk cell detachment appears to occur at tricellular junctions, indicating that these junctions may be important as an anchor point to resist the mechanical forces that occur during cell rearrangements. Hence, these tricellular contacts appear to be essential for generation and maintenance of multicellular configuration during vascular tube formation. A study performed in MDCK cells has shown that Afadin accumulates at tricellular junctions in response to tension (Choi et al., 2016). This finding supports the interesting notion that Rasip1 may play a specific role in reinforcing tricellular junctions during sprouting angiogenesis. Interestingly, *heg1* and *ccm1* knockdown mimics the junctional defects of *rasip1* mutants during sprouting angiogenesis and anastomosis. *In vitro* experiments have shown that Rasip1 localization at cell junctions requires its interaction with the orphan receptor Heg1 (de Kreuk et al., 2016), and it has been suggested that Rasip1 acts in concert with Heg1, Rap1 and Ccm1 and other proteins in junction stabilization (reviewed by Lampugnani et al., 2017). Our phenotypic analyses are in agreement with this concept. In particular, in ISV sprouts we observed cell-pairing defects similar to those of *rasip1* mutants. During anastomosis, *heg1* and *ccm1* morphants displayed junctional-clearance defects (discussed below). Moreover, loss of Heg1 and Ccm1 function causes a partial delocalization of Rasip1 from cell junctions, suggesting that junctional localization is essential for Rasip1 to exert its function during formation and remodeling of endothelial cell junctions.

### Rasip1 is required for lumen patency

Lumen formation in *rasip1* mutants is delayed and ISVs as well as the dorsal aorta show reduced vessel diameters during early embryonic development. At 48 hpf, we observed that about 50% of the ISVs were not patent and this defect was maintained at least until day 4 of development. Defects in luminal patency may have multiple causes. First, as described above, junctional detachment can prevent endothelial cell pairing and thus multicellular tube formation. Second, our examinations of the apical membrane using Podxl-EGFP and Cherry-CAAX transgenic markers revealed an irregular shape of the apical membrane, suggesting luminal collapse. This observation agrees with earlier findings that Rasip1 regulates cortical actin tension during lumen opening of the dorsal aorta in mouse (Barry et al., 2016).

During DLAV formation, we also observed luminal pockets, which were not labeled by microangiography and appeared outside of cell junctions, indicating that these lumens are intracellular and consist of large vesicles or vacuolar structures. Rasip1 has been shown to associate with early Rab5-positive and recycling Rab8-positive endosomes (Barry et al., 2016) and Rab8 has been implicated in the transport of Podocalyxin to the apical membrane in a Cdc42-dependent manner (Bryant et al., 2010). Further analyses will have to be undertaken to determine whether these intracellular lumens are endosomal compartments and whether Rasip1 plays a role in targeting recycling endosomes to the apical compartment.

### Rasip1 during anastomosis

Vascular anastomosis is the process by which blood vessels connect and form a network. Formation of the DLAV in the zebrafish is initiated by the interaction of two neighboring tip cells, which establish contact and form a localized *de novo* lumen at their interface (Herwig et al., 2011; reviewed by Betz et al., 2016). Formation of this luminal pocket follows a relatively stereotyped sequence: upon initial interfilopodial contact, a junctional spot is formed, which is transformed into a ring surrounding apical membrane. This spot-to-ring transformation entails the formation and expansion of a stable junctional ring and the removal of junctional proteins from the center to permit formation of an apical membrane compartment. Loss of Rasip1 prevents apical clearance, leading to ectopic junctions within newly formed apical compartments. A similar phenotype has been observed in *Rasip1* mouse mutants during lumen formation of the DA and it was shown that this requirement is upstream of Cdc42 (Barry et al., 2016). Thus, the molecular mechanisms driving apical clearance during vasculogenesis and anastomosis appear to be conserved.

Rasip1, Radil and Arghap29 have been shown to form a complex and are thought to regulate RhoA. Our analysis of *radil-b* and *rasip1/radil-b* double mutants has shown that both proteins have similar functions during angiogenic sprouting and lumen formation and maintenance. However, Radil-b appears to be dispensable for apical junctional re-localization during anastomosis. In agreement with this interpretation, studies in the mouse DA have shown that clearance of apical junctions requires Cdc42 and is independent of RhoA (Barry et al., 2016).

In the developing mouse DA, loss of Rasip1 leads to an overactivation of Rock and an increase of cortical actomyosin tension in the apical compartment (Barry et al., 2016). As a consequence, the luminal surface of the endothelium cannot expand and the lumen is constricted. In relation to this, we observe a collapse of the junctional ring during anastomosis whereas the junctions are still maintained and elongate along the vascular axis, resulting in a narrower apical compartment within the collapsed ring, eventually leading to a close alignment of the junctions along the extending axis. We speculate that this collapse of the junctional ring may be caused by an imbalance of the cortical actin between the apical and basolateral compartments, caused by an overactivation of Rock at the apical side. Further studies on local actomyosin regulation will be required to understand better the formation of a luminal surface during vasculogenesis and vascular anastomosis.

## MATERIALS AND METHODS

### Zebrafish strains and morpholinos

Zebrafish were maintained according to FELASA guidelines (Aleström et al., 2019). All experiments were performed in accordance with federal guidelines and were approved by the Kantonales Veterinäramt of Kanton Basel-Stadt (1027H, 1014HE2, 1014G). Zebrafish lines used were *Tg(fli1a:EGFP)*^y1^ (Lawson and Weinstein, 2002), *Tg(gata1a:DsRed)*^sd2^ (Traver et al., 2003), *Tg(kdrl:EGFP)^s843^* (Jin et al., 2005), *Tg(kdrl:EGFPnls)^ubs1^* (Blum et al., 2008), *Tg(5xUAS:RFP)* (Asakawa and Kawakami, 2008), *Tg(fli1ep:gal4ff)^ubs3^* (Herwig et al., 2011), *Tg(fli1a:Pecam-EGFP)^ncv27^* (Ando et al., 2016), *Tg(cdh5:cdh5-TFP-TENS-Venus)^uq11bh^* (Lagendijk et al., 2017), *Tg(UAS:mRuby2-UCHD)^ubs20^* (Paatero et al., 2018), *Tg(UAS:EGFPpodxl)^ubs29^* (this study) and *rasip1^ubs28^* (this study) and *radil-b^sa20161^* (European Zebrafish Resource Center, Karlsruhe, Germany). Morpholinos (Gene-Tools) used were as follows: *ccm1* 5′-GCTTTATTTCACCTCACCTCATAGG-3′ (Mably et al., 2006), *heg1* 5′-GTAATCGTACTTGCAGCAGGTGACA-3′ (Mably et al., 2003), standard control 5′-CCTCTTACCTCAGTTACAATTTATA-3′.

### Generation of *rasip1* mutant alleles

For this study, three *rasip1* mutants was generated. Two gRNA sites were selected for a null mutant (*rasip1^ubs28^* mutant) using an online tool http://www.crisprscan.org (Moreno-Mateos et al., 2015) based on a high score:

Cris6 (GGCGGGGGAAGGGGATGGAGAGG, exon3, score 101) and Cris7 (TGAAGCTCAGGGCTGGGGATTGG, exon16, score 63).

For *rasip1^ubs23^* and *rasip1^ubs24^* mutant, target sites were chosen: Cris1 (GGAATGTCCCTTACAGCTGGTGG, exon3); Cris2 (GGCGGGGGAAGGGGATGGAGAGG, exon 2); Cris3 (GGACAAGACAGGTAGCGGAGGGG, exon12); Cris4 (GGTGGAGTGAGAGAGGGAGG, exon2);

Cris5 (GGCGGGACGGGAGTCACACGCGG, exon7).

gRNAs/Cas9 injections were performed according to Gagnon et al. (2014). gRNAs were cloned into vector DR274. The injection mixture consisted of (final concentration): Cas9 protein (1 μg/μl); KCl (0.2 M), sgRNA (0.2 μg/μl). One to two nanoliters of injection mixture were injected into 1- to 2-cell-stage embryos. Mutants were identified by sequencing the genomic target region.

### Genotyping of *rasip1* and *radil-b* mutant alleles

*rasip1*and *radil* mutants were identified by multiplex-PCR using combinations of non-specific and allele-specific primers. Primer sequences are listed in Table S1.

### Generation of *Tg(UAS:EGFP-podxl)^ubs29^*

The p5E-4xnrUAS promoter, pME-EGFP-podocalyxin (Navis et al., 2013) and p3E-polyA (Kwan et al., 2007) were cloned into a Tol2 vector pDestTol2CG2 carrying cmcl2:GFP to drive expression of GFP in the heart. The final plasmid was co-injected with tol2 mRNA into the *Tg(fli1ep:gal4ff)^ubs3^;(UAS:mRFP)* embryos. These mosaic embryos were raised to adulthood and outcrossed with the parental fish line to generate stable fish lines. The resulting *Tg(UAS:EGFP-podxl)* embryos were identified on the basis of GFP expression in the heart; proper apical localization of EGFP-Podocalyxin was confirmed using confocal microscopy. Two transgenic lines, ubs29 and ubs30, were isolated, and the ubs29 line showing more homogenous expression levels in endothelium was used in experiments.

### Transient expression of Rasip1-p2a-tdTomato-CAAX in zebrafish embryos

To express Rasip1 transiently in endothelial cells of zebrafish embryos, transposase mRNA/plasmid DNA injections were performed according to Suster et al. (2009). fli:Rasip1-p2a-tdTomato-CAAX and fli:tdTomato-Caax constructs were cloned into vector PDestTol2CG2 through gateway cloning. Injection mix was 10 μl of 0.2 M KCl, 2 μl of Phenol Red solution, 2 μl of 250 ng/μl transposase mRNA, 2 μl of 250 ng/μl plasmid DNA, and 4 μl of water to reach a final volume of 20 μl. Around 1 nl of the mixture was injected into 1- to 4-cell-stage *rasip1^ubs28^* embryos. Thirty hours after injection, healthy embryos expressing tdTomato were selected. Embryos are mounted in low-melting point agarose and imaged using a Leica SP5 confocal microscope.

### Immunofluorescence

Immunofluorescence was performed as previously described (Herwig et al., 2011). The following antibodies were used: rabbit anti-zf-Cdh5 (1:200; Blum et al., 2008), rabbit anti-Esama (1:200; Sauteur et al., 2017), mouse anti-human-Zo-1 (1:100; Thermo Fisher Scientific, 33-9100), rabbit anti-Rasip1 (1:500; this paper), chicken anti-GFP (1:200; Abcam, ab13970), Alexa 405 goat anti-chicken immunoglobulin Y (IgY H&L) (1:1000; Abcam, ab175674), Alexa 568 goat anti-rabbit immunoglobulin G (IgG) (1:1000; Thermo Fisher Scientific, A-11011) and Alexa 633 goat anti-mouse IgG (1:1000; Thermo Fisher Scientific, A-21053). The anti-zf-Rasip1 antibodies were raised in rabbits against a synthetic peptide (CRTFLWGLDQDELPANQRTRL-COOH) comprising the terminal amino acid residues (aa 970-989) of the protein (YenZym Antibodies)

### Live imaging

Time-lapse imaging was performed as previously described (Paatero et al., 2018). All movies were taken with Leica SP5 or SP8 confocal microscopes using a 40× water immersion objective (NA=1.1) with a frame size of 1024×512 or 1024×1024 pixels. Routinely, *z*-stacks consisted of 80-100 slices with a step size of 0.8-1 μm. Stacks were taken every 8 or 10 min. High-resolution imaging was performed on a Zeiss LSM880 microscope using a 40× water immersion objective (NA=1.2) using a vertical step size of 0.25 µm.

### Cell transplantation

Embryos were collected at the 1-cell stage in 1× E3 medium and immediately enzymatically dechorionated using 1 mg/ml pronase (Millipore-Sigma) in 1× E3 medium+1×10^−4^% (w/v) Methylene Blue, pH 7.0. Pecam-eGFP wild-type donors were injected at the 1-cell stage with 0.2 ng of 10 kDa tetramethylrhodamine-dextran conjugate (Thermo Fisher Scientific) in a volume of 1 nl. All embryos were kept in 1× E3 medium+1×10^−4^% (w/v) Methylene Blue, pH 7.0, on 2% agarose-coated plastic dishes at 28.5°C unless stated otherwise.

Transplantations were conducted at sphere stage in 1× Ringer's solution (116 mM NaCl, 2.8 mM KCl, 1 mM CaCl_2_, 5 mM HEPES), pH 7.0, using a custom-made apparatus. Briefly, a 3 ml syringe with Luer-Lok tip (BD) was connected to a PicoNozzle v2 tip assembly (World Precision Instruments) using 1/16-inch inner-diameter polyurethane tubing and a corresponding female Luer hose barb adapter (Cole-Parmer). The PicoNozzle was mounted on a Narishige M-152 manipulator and used to hold a filament-less borosilicate glass needle. The syringe plunger was gently retracted to draw cells into the needle and depressed to discharge cells into host embryos. Approximately 50 cells were removed from the animal-pole of wild-type donors and placed at margin of mutant hosts. In the case of Pecam-EGFP donors and *rasip1^ubs28^* mutant hosts, cells were placed at two separate locations along the margin to increase the probability of donor cells developing into intersegmental vessels. Upon completion of transplantations, host embryos were promptly returned to 1× E3 medium+10^−4^% (w/v) Methylene Blue, pH 7.0, on 2% agarose-coated plastic dishes at 28.5°C until fixation or mounting for imaging. In experiments in which *rasip1^ubs28^* mutant hosts expressed UCHD-mRuby2, *Tg*(*fli1a:EGFP)^y1^* embryos were used as wild-type donors.

### Phylogenetic comparison of Rasip1 and Radil homologs

The analysis was carried out using the online program *phylo.io* (http://dev.phylo.io/) (Robinson et al., 2016). The following peptide sequences were used: *Mus musculus* (mouse) Rasip1: ENSMUSG00000044562; *Homo sapiens* (human) Rasip1: ENSG00000105538; *Danio rerio* (zebrafish) Rasip1: ENSDART00000155407.3; *Mus musculus* (mouse) Radil: ENSMUSG00000029576; *Homo sapiens* (human) Radil: ENSG00000157927; *Danio rerio* (zebrafish) *radil-a*: ENSDARP00000101722; *radil-b*: ZDB-GENE-130530-682 si:ch73-281f12.4; *radil-c*: ZDB-GENE-121214-224 si:ch211-176g6.2.

### Statistics

Unless explicitly stated, all results shown were obtained from at least three independent experiments, sample sizes were not predetermined, the experiments were not randomized and investigators were not blinded to allocation during experiments and outcome assessment. Experiments performed on genetic mutants were blinded by post-experimental determination of genotypes. Statistical analyses were performed using Prism software (GraphPad) and ordinary unpaired two-tailed Mann–Whitney test.

## Supplementary Material

Supplementary information

Reviewer comments
