# Supplementary material for: Control of dynamic cell behaviors during angiogenesis and anastomosis by Rasip1
Source: Development. 2021 Aug 12;148(15):dev197509. doi: 10.1242/dev.197509 (PMC8380458; doi:10.1242/dev.197509)
Supplement: Supplementary information [file develop-148-197509-s1.pdf]

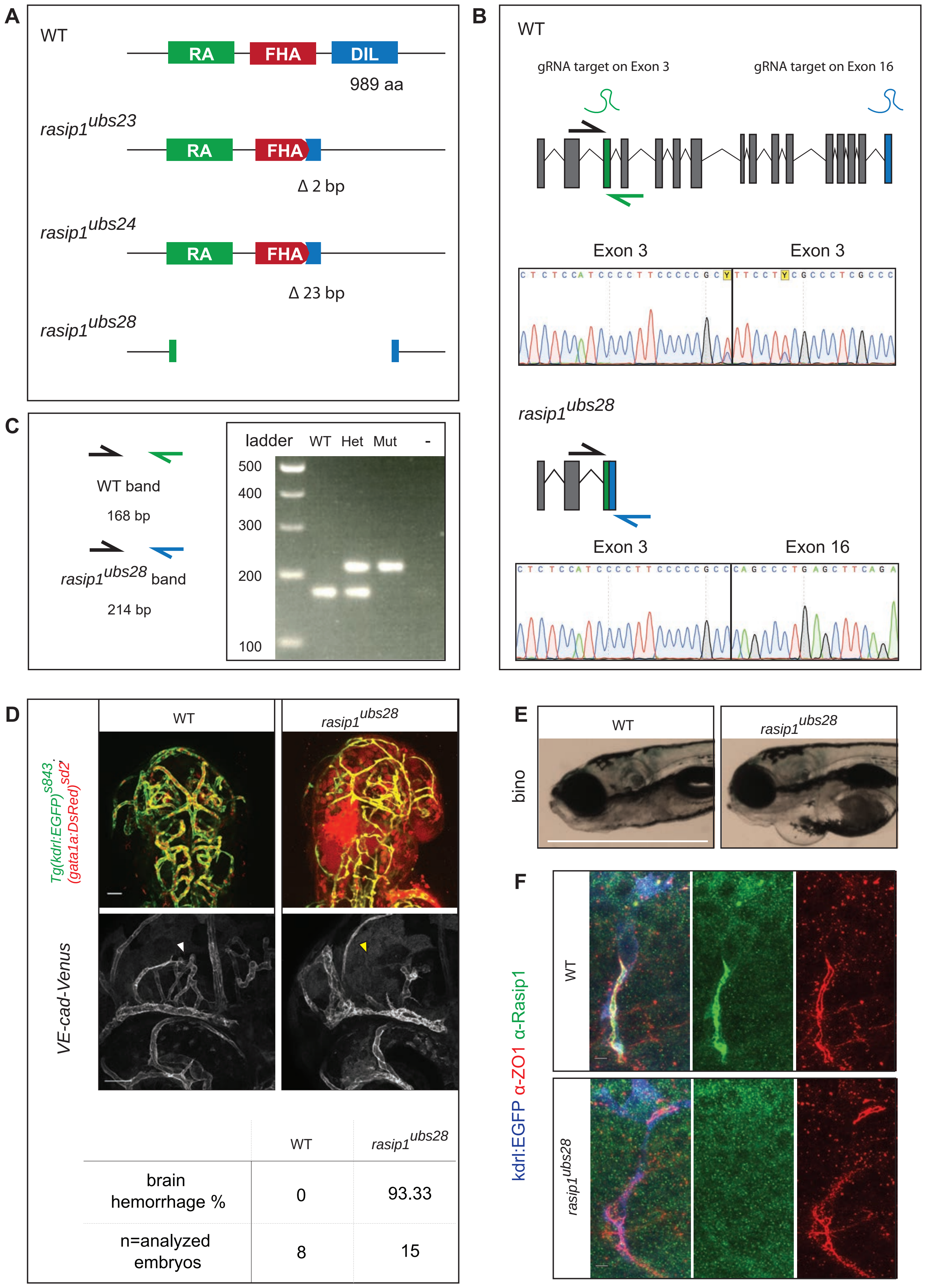

Development • Supplementary information

Fig. S1. Characterization of *rasip1* mutant alleles. (**A**) Schematic representation of the *rasip1* locus in wild-type, *rasip1^{ubs23}*, *rasip1^{ubs24}* and *rasip1^{ubs28}* alleles. Conserved protein domains in Rasip1. RA: Ras association domain. FHA forkhead-association domain. DIL: dilute domain. **(B)** Design of gRNAs to target exon3 and exon16. The wild-type DNA sequence of exon3 is shown. The *rasip1^{ubs28}* mutant allele consists of a 35kb deletion from exon3 to exon16, comprising all three conserved protein domains. **(C)** PCR strategy to identify the *rasip1^{ubs28}* allele. **(D)** Cranial vascular defects in rasip1ubs28 mutant embryos. Top panels: confocal images showing cranial hemorrhages in rasip1ubs28 embryo (72 hpf). Blood cells are visualized by gata1:dsRed expression. Scale bar, 50 µm. Middle panels: vascular morphogenesis defects in the midbrain of *rasip1^{ubs28}* embryo. Endothelial cell junctions are visualized by VE-cad-Venus (Tg(*cdh5:cdh5-TFP-TENS-Venus*) $^{uq11bh)}$. The basal communicating artery and the middle mesencephalic central arteries do not form in *rasip1^{ubs28}* embryo (yellow arrow head). Scale bar, 20 µm. The quantification of cranial hemorrhages in rasip1ubs28 mutant embryos. p<0.0001 (Fisher's exact test). **(E)** Bright field image of wild-type and *rasip1^{ubs28}* embryo showing pericardial edema (120 hpf). Scale bar, 2 mm. **(F)** I mmunofluorescent staining of Rasip1 (green) in the zebrafish vasculature (32 hpf). The anti-zf-Rasip1 antibody is directed against the C-terminal domain of the protein (see Materials and Methods). The endothelium is labeled by *Tg(kdrl:EGFP)^{s843}* (blue) and junctions are labeled by Zo-1 (red). Rasip1 protein is not detectable in *rasip1^{ubs28}* mutants. Scale bars, 5 µm.

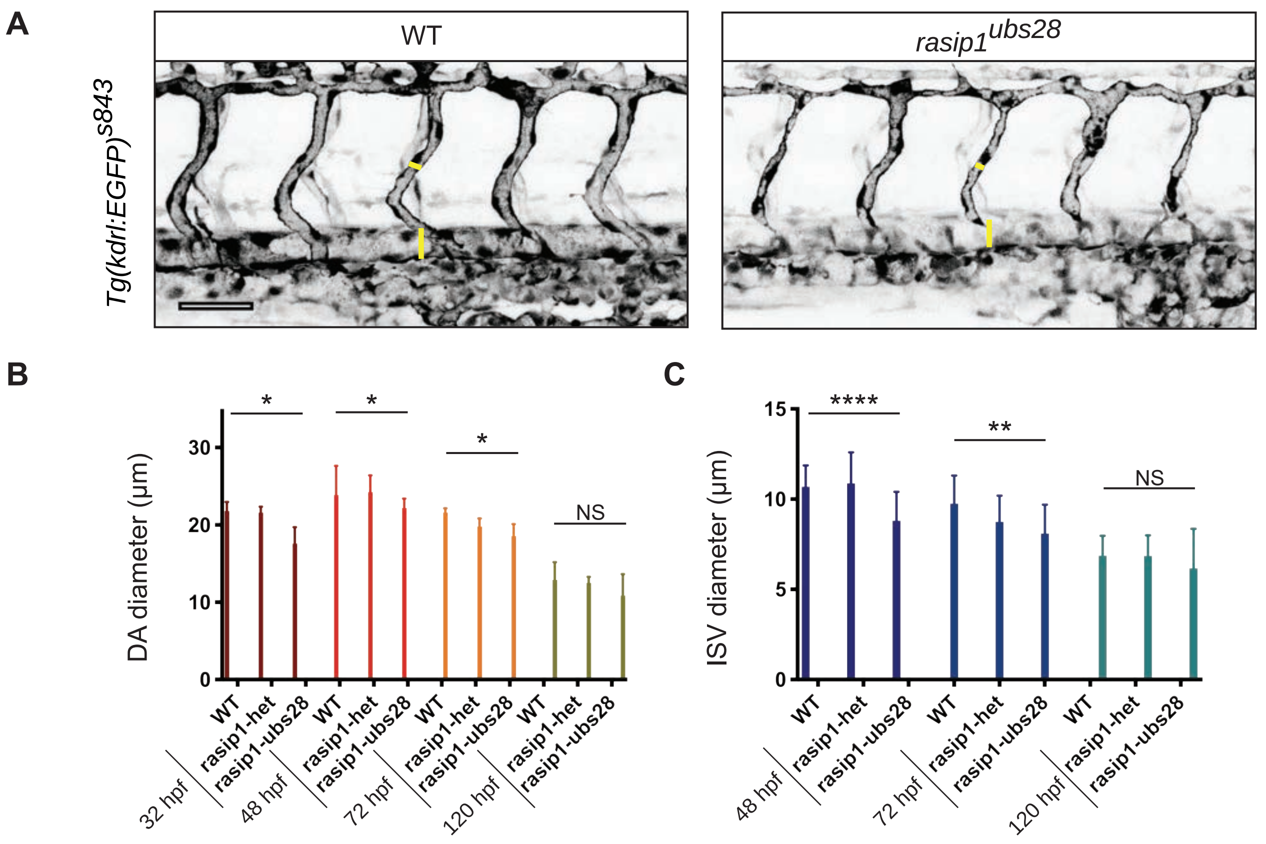

Fig. S2. Reduced blood vessel caliber in zebrafish *rasip1* mutants. (A) Confocal images of wild-type and *rasip1*^{ubs28} mutant. The *rasip1*^{ubs28} mutant shows narrower DA and irregular ISV diameters. Scale bar, 50 µm. **(B)** Quantification of DA diameters (µm) during embryonic development (32 to 120 hpf). Mann-Whitney test and error bars indicate standard deviation; significance (ns=not significant, *p < 0.1).(WT 32, 48, 72, 120 hpf: *n*=4, 4, 9, 13 embryos; *rasip1*$^{ubs28/+}$ *n*=5, 12, 19, 33; *rasip1*^{ubs28} *n*=6, 10, 9, 10). **(C)** Quantification of ISV diameters (µm) during embryonic development (32 to 120 hpf). Embryos were analyzed by unpaired two-tailed Mann-Whitney test and error bars indicate standard deviation; significance (ns=not

Development • Supplementary information

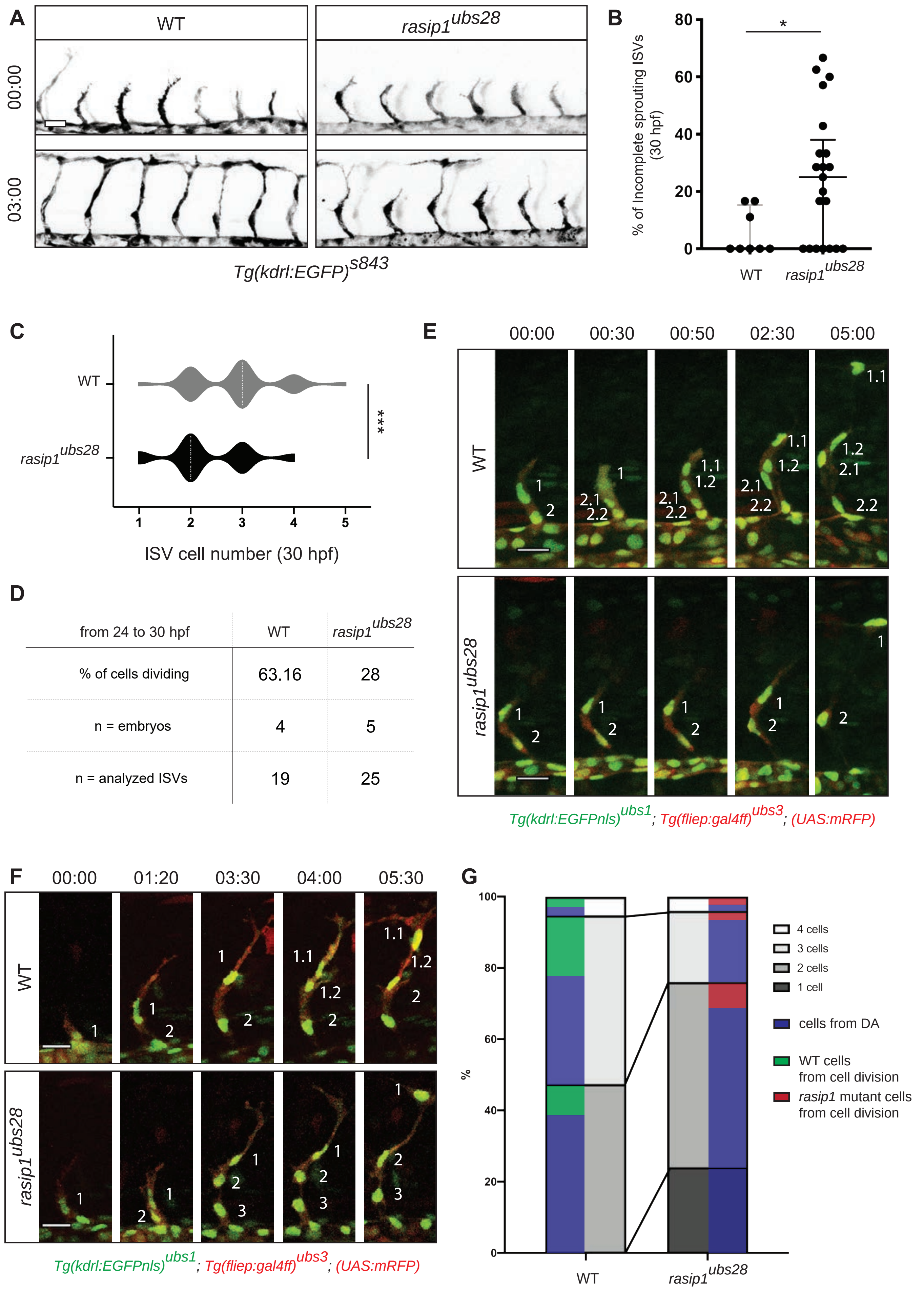

from 24 to 30 hpf	WT	rasip1ubs28
% of cells dividing	63.16	28
n = embryos	4	5
n = analyzed ISVs	19	25

Development • Supplementary information

Fig. S3. ISV sprouting is affected in *rasip1* mutants. (A) Still pictures of time-lapse movies showing ISV sprouting in wild-type and rasip1^{ubs28} embryo between 27 and 30 hpf (Movies 15, 16). Scale bars, 20 µm. **(B)** Quantification of incomplete ISVs at 30 hpf (WT n=8 embryos, mut n=21). Median value: WT=0, mut=25%. Sprouting ISVs showing incomplete growth were counted and divided by the total ISV number per embryo. (unpaired two-tailed Mann-Whitney test and error bars indicate standard deviation; significance: *p < 0.1) **(C)** Proportion of ISVs of different cell numbers at 30 hpf (WT n=12 embryos, 58 ISVs; mut n=12, 60). Unpaired two-tailed Mann-Whitney test and error bars indicate standard deviation; significance: ***p < 0.001. **(D)** Cell division rates from 24 to 30 hpf are decreased in rasip1^{ubs28} compared to wild-type. Embryos were analyzed by Fisher exact test: p=0.0316. **(E,F)** Still-pictures of time-lapse analysis showing endothelial cell proliferation and movements (visualized by nuclear EGFP) in wild-type and rasip1^{ubs28} embryos (Movies 17-20). rasip1 mutants show reduced cell proliferation within the sprout (E). Reduced cell number may be partially compensated by migration of additional cells into the sprout (F). Scale bars, 5 µm. **(G)** Quantification of time-lapse analyses on the relative contribution (%) of cell migration and proliferation to ISVs of different cell content. The ratio of cells from divisions and cells originated from the DA were quantified in rasip1^{ubs28} compared to wild-type (WT n=4, mut n=5).

Development • Supplementary information

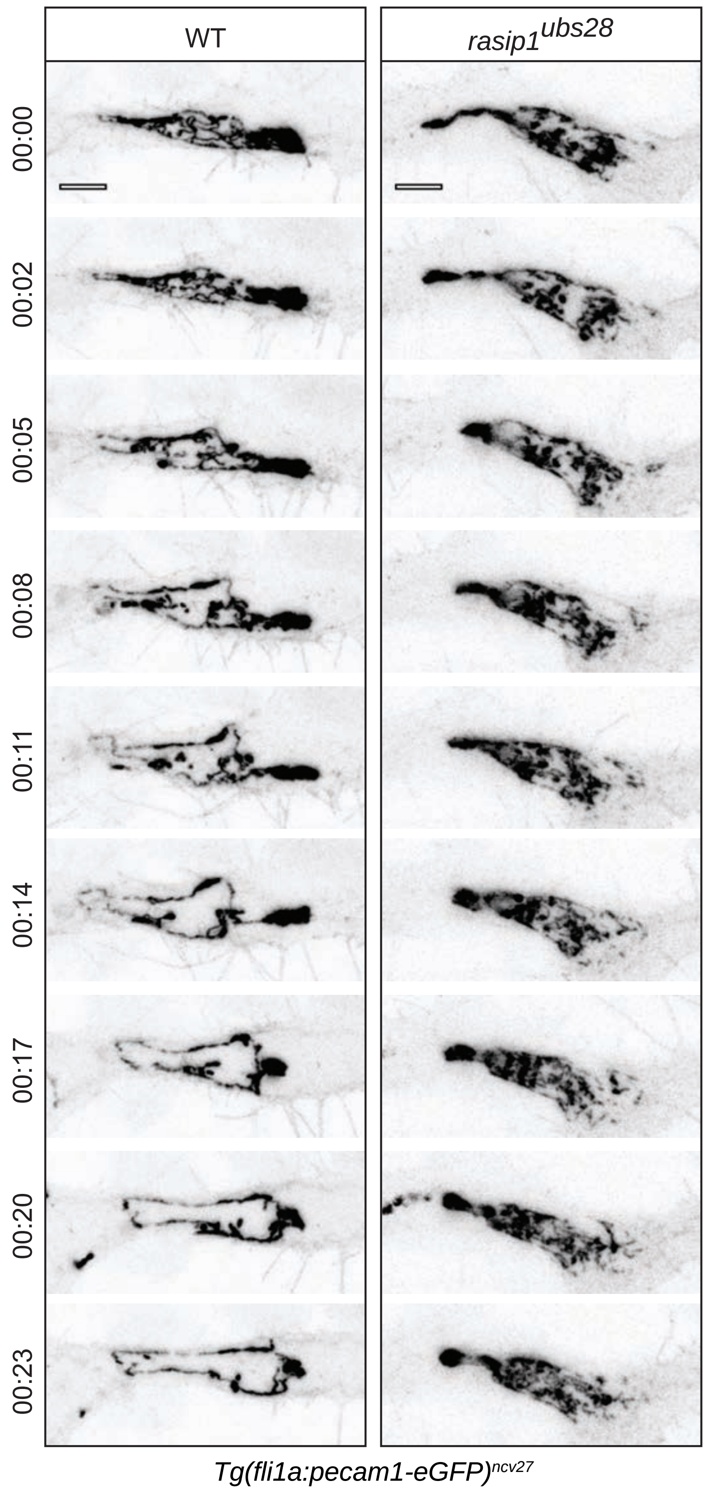

Fig. S4. Re-localization of junctional molecules from the apical region during anastomosis. Still images with high spatial and temporal resolution (hh:mm) from a movie of a PECAM-EGFP expressing embryos Tg(fli1a:Pecam-EGFP)^{ncv27} (Movies 21, 22). DLAV junctions were imaged from 32 hpf onwards. Scale bar, 5 µm.

Development • Supplementary information

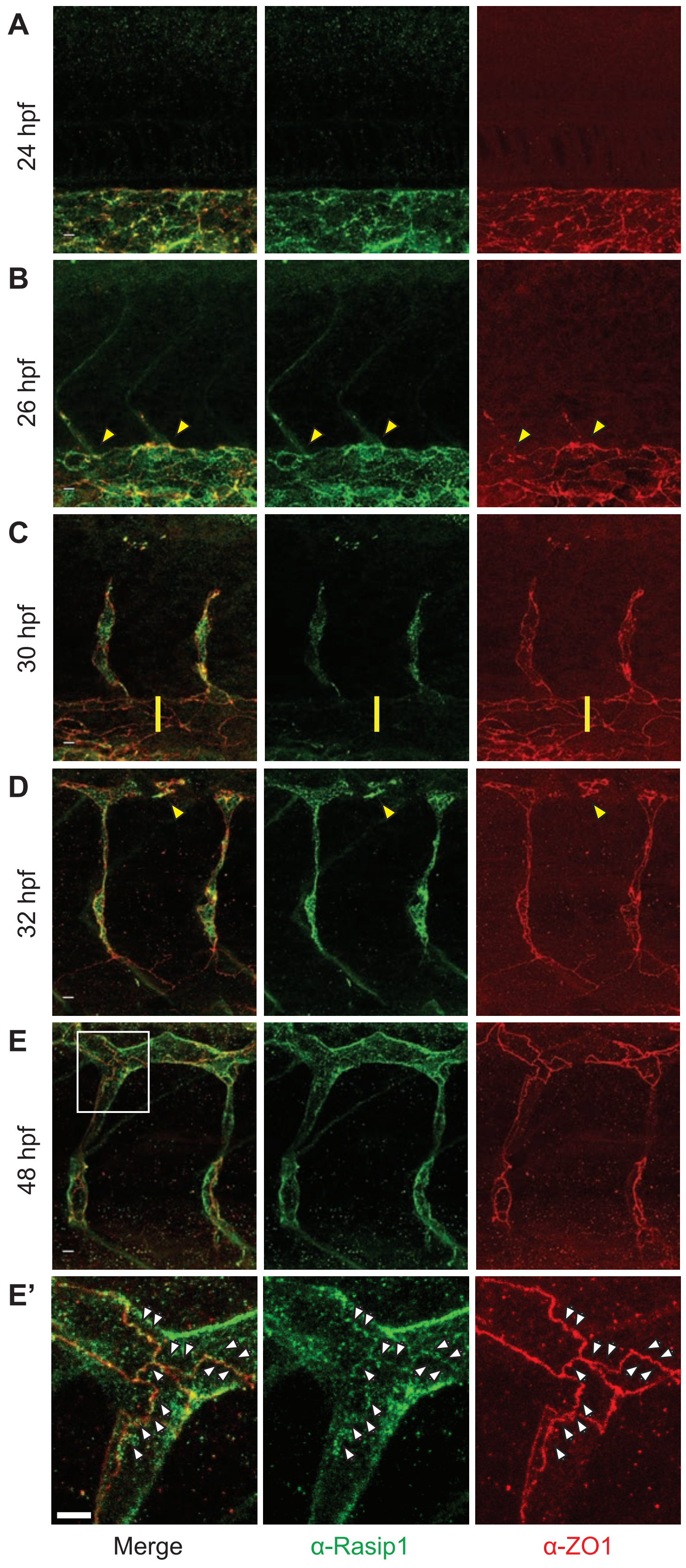

Fig. S5. Dynamic distribution of Rasip1 during vascular development. (A-E) Immunofluorescence of Rasip1 and Zo-1 at different developmental stages. Rasip1 protein is specifically expressed in the developing vasculature, visible in the DA at 24 hpf (A) and then in sprouting endothelial cells at 26 hpf (yellow arrowheads) (B). Expression in the DA is lost at 30 hpf (yellow bar) (C). Rasip1 is apically localized at 30-32 hpf (yellow arrowheads, C and D) and also detectable at endothelial cell junctions at 48 hpf (white arrowheads in close ups, E'). Scale bar, 20 µm.

Development • Supplementary information

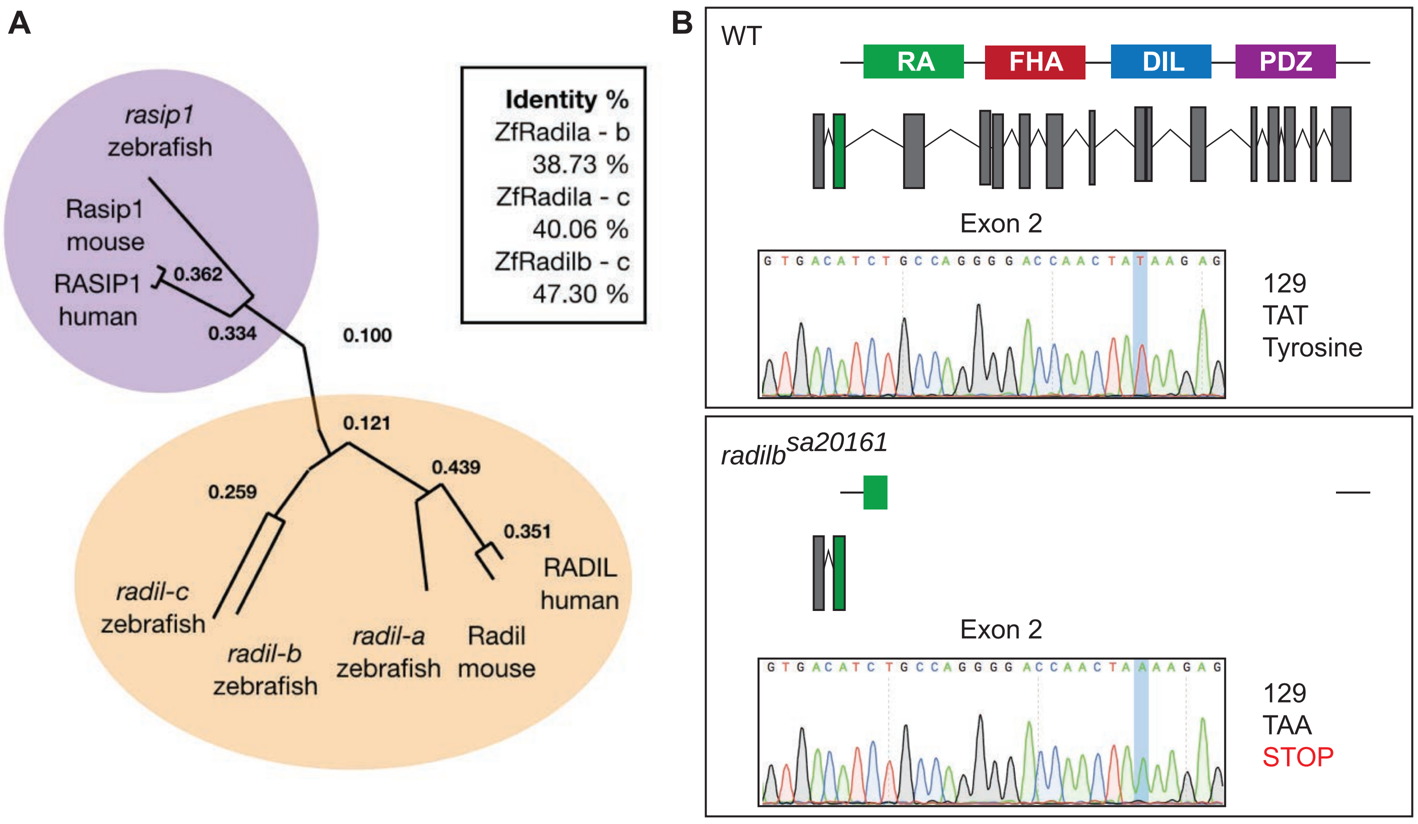

C

72 hpf	WT	$rasip1^{ubs28}$	$radilb^{sa20161}$	Double mutant
brain hemorrhage %	0	93.33	87.5	87.5
n=analyzed embryos	8	15	16	8

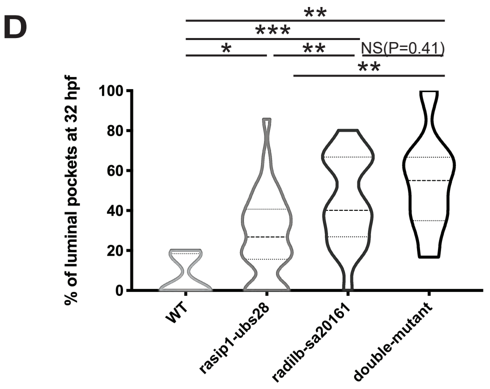

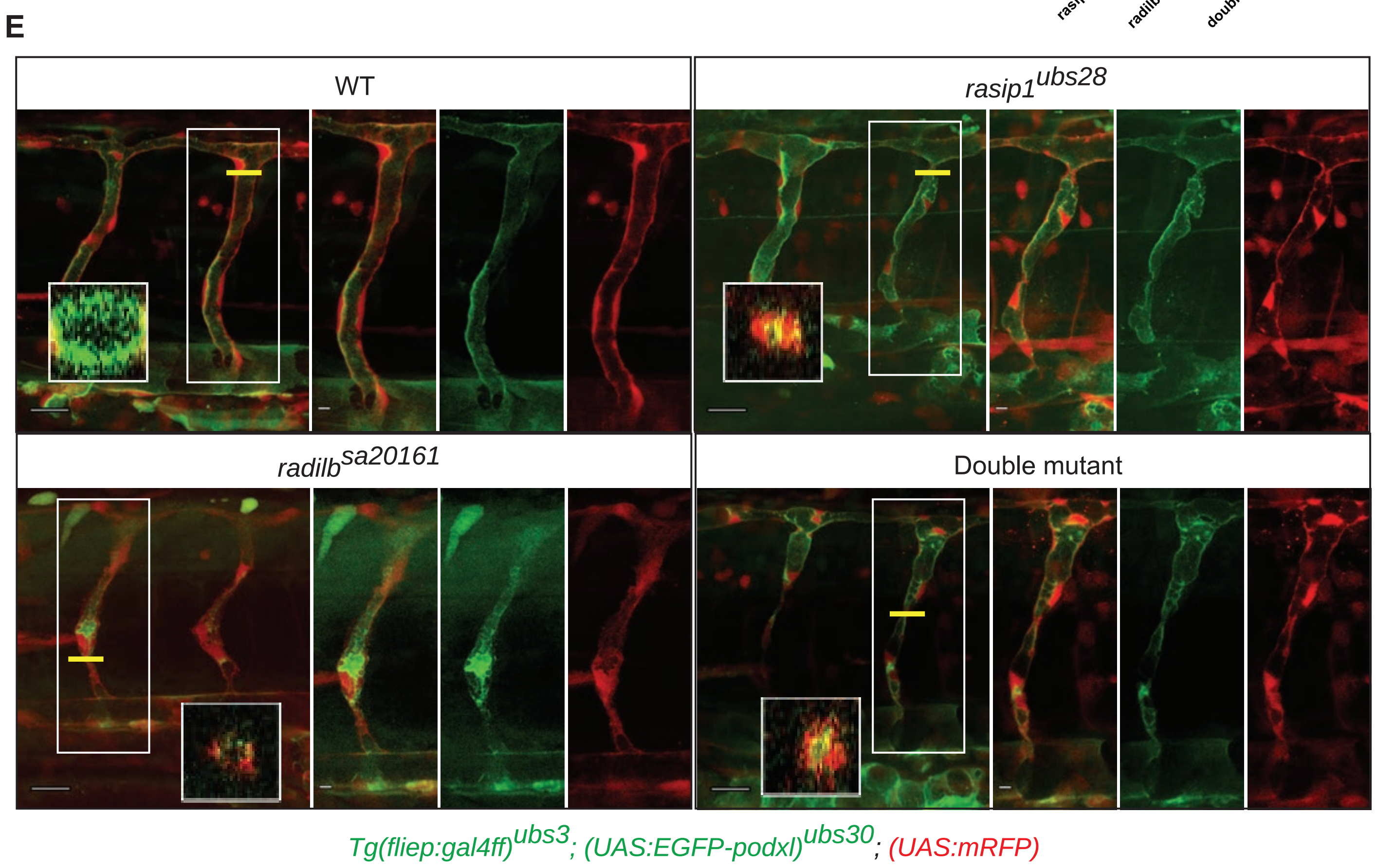

Development • Supplementary information

Fig. S6. Loss of *radil-b* enhances lumen defects and blood-flow of *rasip1* mutants. (A) Phylogenetic tree based on the alignment of the entire protein sequences of human, mouse and zebrafish rasip1 and radil. There are three radil paralogues in zebrafish. Numbers at branch points present bootstrap values. Zebrafish Radil-a has much closer relationship with regard to its mouse and human homologue based on protein-protein interaction databases. Radil-b and Radil-c were newly identified in this study and annotated from organism-specific databases. **(B)** A nonsense mutation in exon2 of radil-b^{sa20161} mutants ablates the Ras association (RA) domain, the dilute (DIL) domain (Rasip1 binding site) and the PDZ domain. **(C)** Quantification of cranial brain hemorrhage in rasip1^{ubs28}, radil-b^{sa20161} and rasip1^{ubs28}; radil-b^{sa20161} double mutants. **(D)** Quantification of luminal pockets from the still images of wild-type, single rasip1^{ubs28} and radilb^{sa20161} and rasip1^{ubs28}; radilb^{sa20161} double mutants at 32 hpf. The number of ISVs containing ectopic lumens is divided by the total number of ISVs analyzed per embryo (WT n=5, rasip1^{ubs28} mut n=34, radilb^{sa20161} mut n=21, rasip1^{ubs28}; radilb^{sa20161} mut n=8). **(E)** Live images showing EGFP-Podxl in WT, rasip1^{ubs28}, radilb^{sa20161} and rasip1^{ubs28}; radilb^{sa2016} double mutants displaying luminal constrictions at 48 hpf (see inset z-projections). Insets show digital cross sections of the ISV (indicated by yellow bars). Scale bars, 20 µm (overview) and 5 µm (inset).

Development • Supplementary information

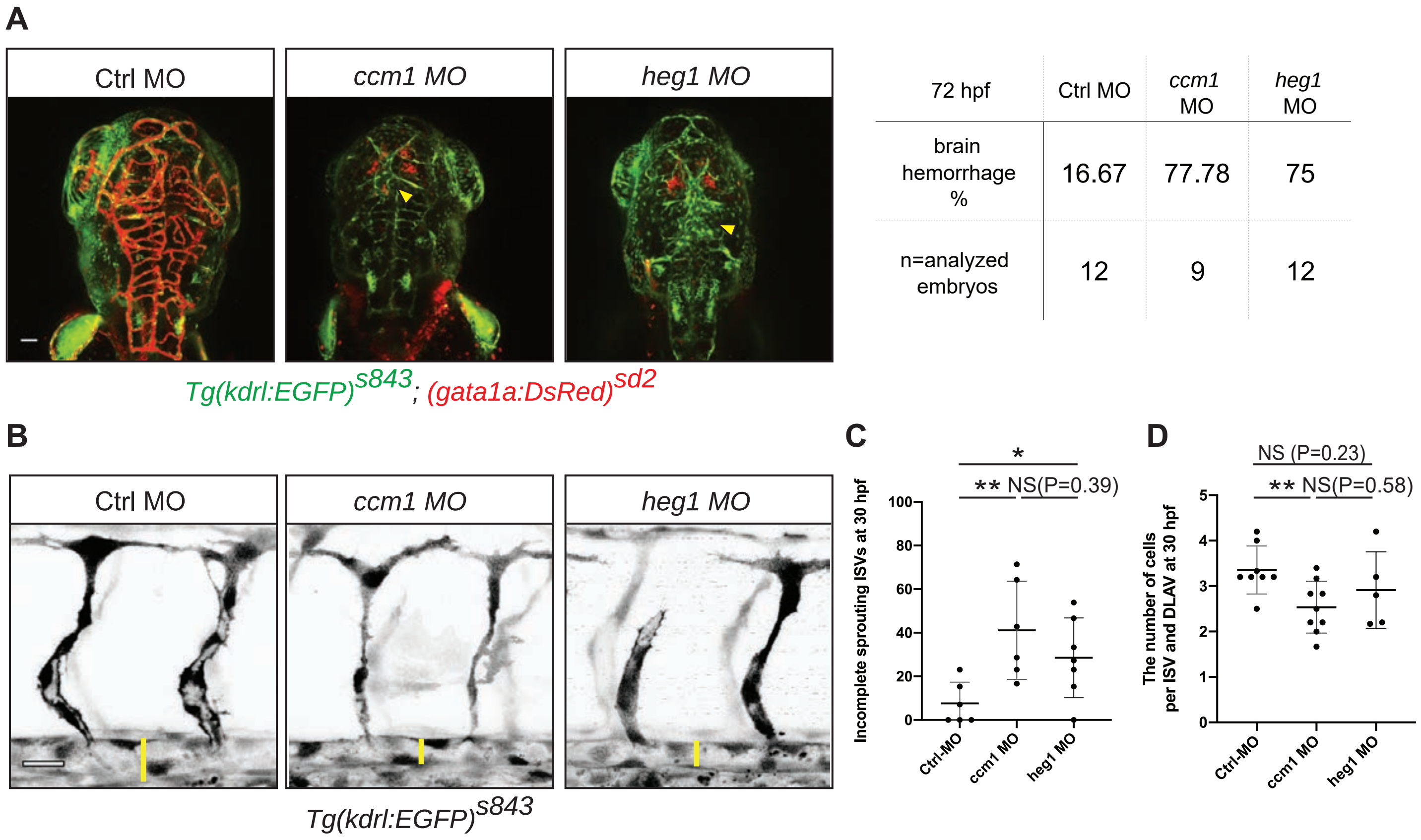

Fig. S7. Vascular defects in *heg1* and *ccm1* morphants. (A) Live images of *Tg(kdrl:EGFP)*^{s843}*; (gata1:Dsred)*^{sd2} at 72 hpf. *ccm1* and *heg1* morphants display mesenphalic hemorrhages while cranial circulation appears completely disrupted. In addition, the MsV (Mesencephalic veins) and DLV (dorsal longitudinal vein) (yellow arrowheads) are malformed. Scale bar, 50 µm. Cranial brain hemorrhages are observed in *ccm1* and *heg1* MO injected embryos with higher incidence when compared to control MO injected embryos. **(B)** Live images of control, *ccm1* and *heg1* morphants at 32 hpf. *ccm1* and *heg1* morphants show reduced DA diameters (yellow bars) and defective ISV formation. Scale bar, 20 µm. (C) Quantification of incompletely sprouting ISVs at 30 hpf (Control MO injected embryos n=6, ccm1 MO n=6, heg1 MO n=7). (D) The number of cells per ISV and DLAV at 30 hpf (Control MO injected embryos n=8, ccm1 MO n=9, heg1 MO n=5). Analyzed by unpaired two-tailed Mann-Whitney test and error bars indicate standard deviation; significance (ns=no significance, *p < 0.1, **p < 0.01).

Table S1. Primer sequences

**Primer**	**Name**	**Sequence (5'-3')**
Rasip1-1	Rasip1-fwd	TGTTGCCATCAGATCCACCAC
Rasip1-2	Rasip1-wt-rev	TTGGCCCGGGATTGCTGATT
Rasip1-3	Rasip1-ubs28-rev	GTCCGCTGATTAGCAGGAAGT
Radilb-1	Radil-b-fwd	CCACAACAACCGGCTAACCAC
Radilb-2	Radil-b-rev	ACAATGAGCCTGGGTTGCAAATAA
Radilb-3	Radil-b-wt-fwd	TGGCCAGCACACTCTTTT
Radilb-4	Radil-b-sa20161-rev	GCCAGGGGACCAACTATA

Development • Supplementary information

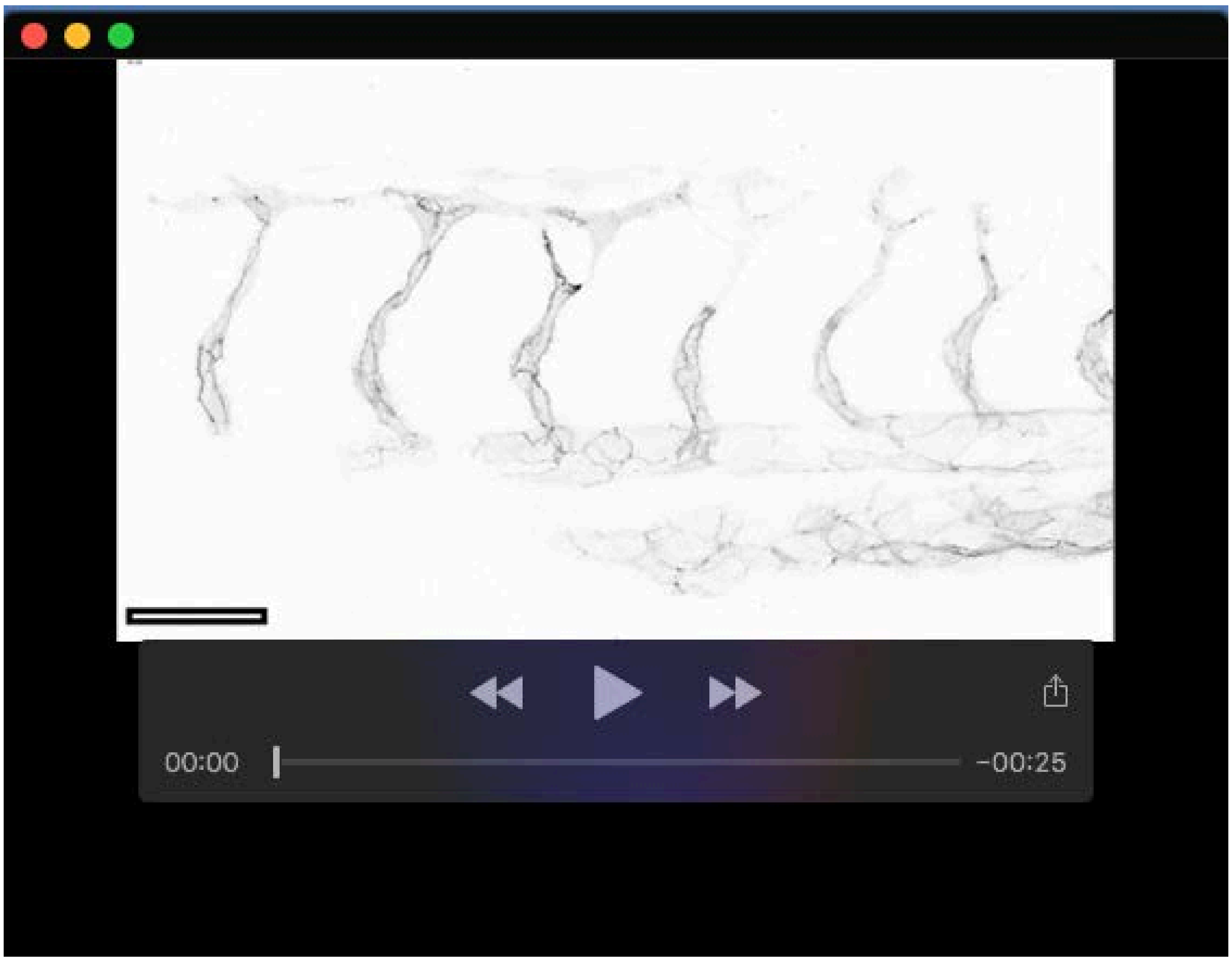

Movie 1 (Main figure 1A): Confocal time-lapse movie of ISV formation (30-48 hpf) in a wild-type and a *rasip1*^{ubs28} mutant embryo. Endothelial cell junctions are labeled by VE-cad-Venus (*Tg(cdh5:cdh5-TFP-TENS-Venus)*^{uq11bh}) and imaged 1frame/h (reverse contrast). Scale bar, 50 µm.

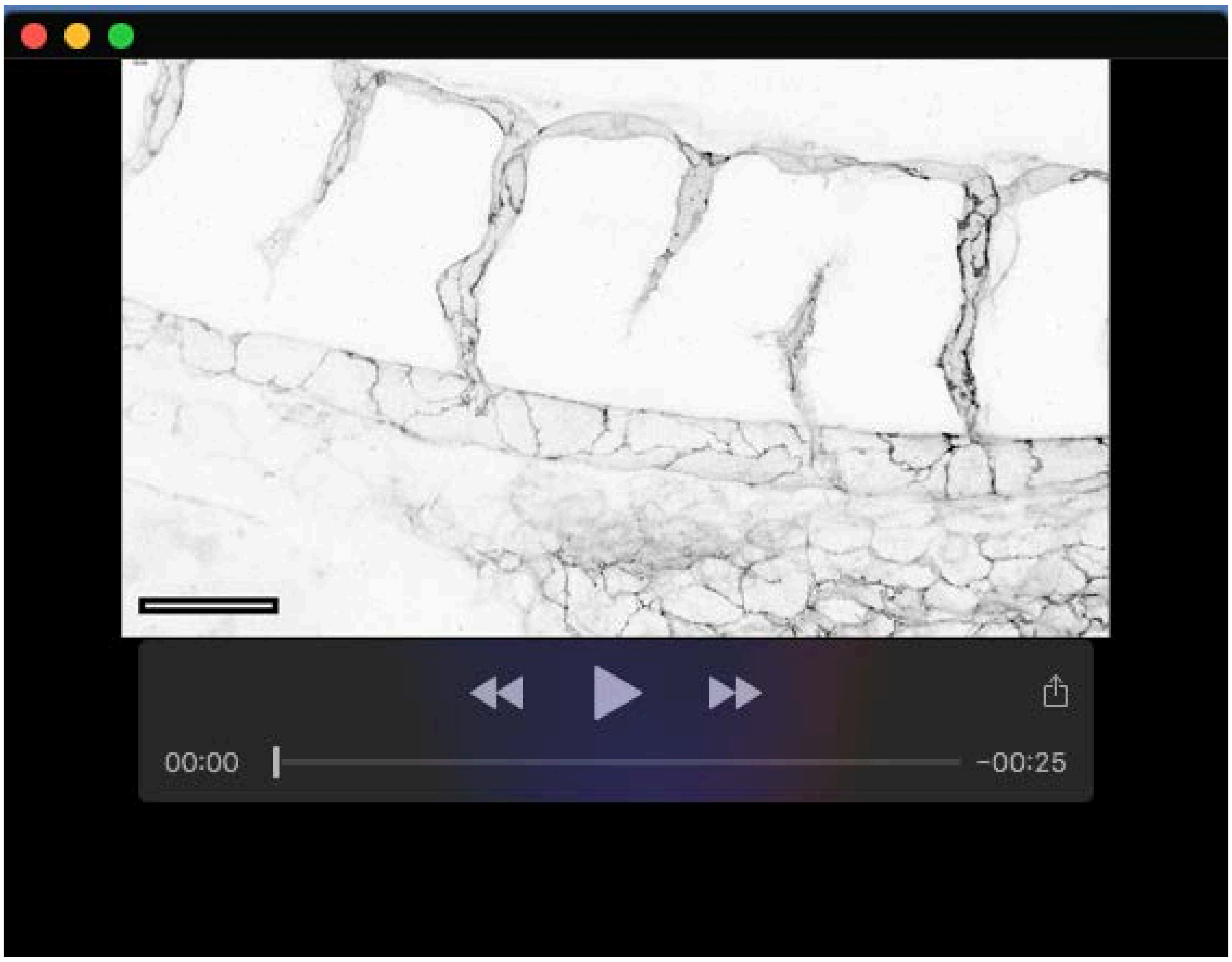

Movie 2 (Main figure 1A): Confocal time-lapse movie of ISV formation (30-48 hpf) in a wild-type and a *rasip1*^{ubs28} mutant embryo. Endothelial cell junctions are labeled by VE-cad-Venus (*Tg(cdh5:cdh5-TFP-TENS-Venus)*^{uq11bh}) and imaged 1frame/h (reverse contrast). Scale bar, 50 μm.

Development • Supplementary information

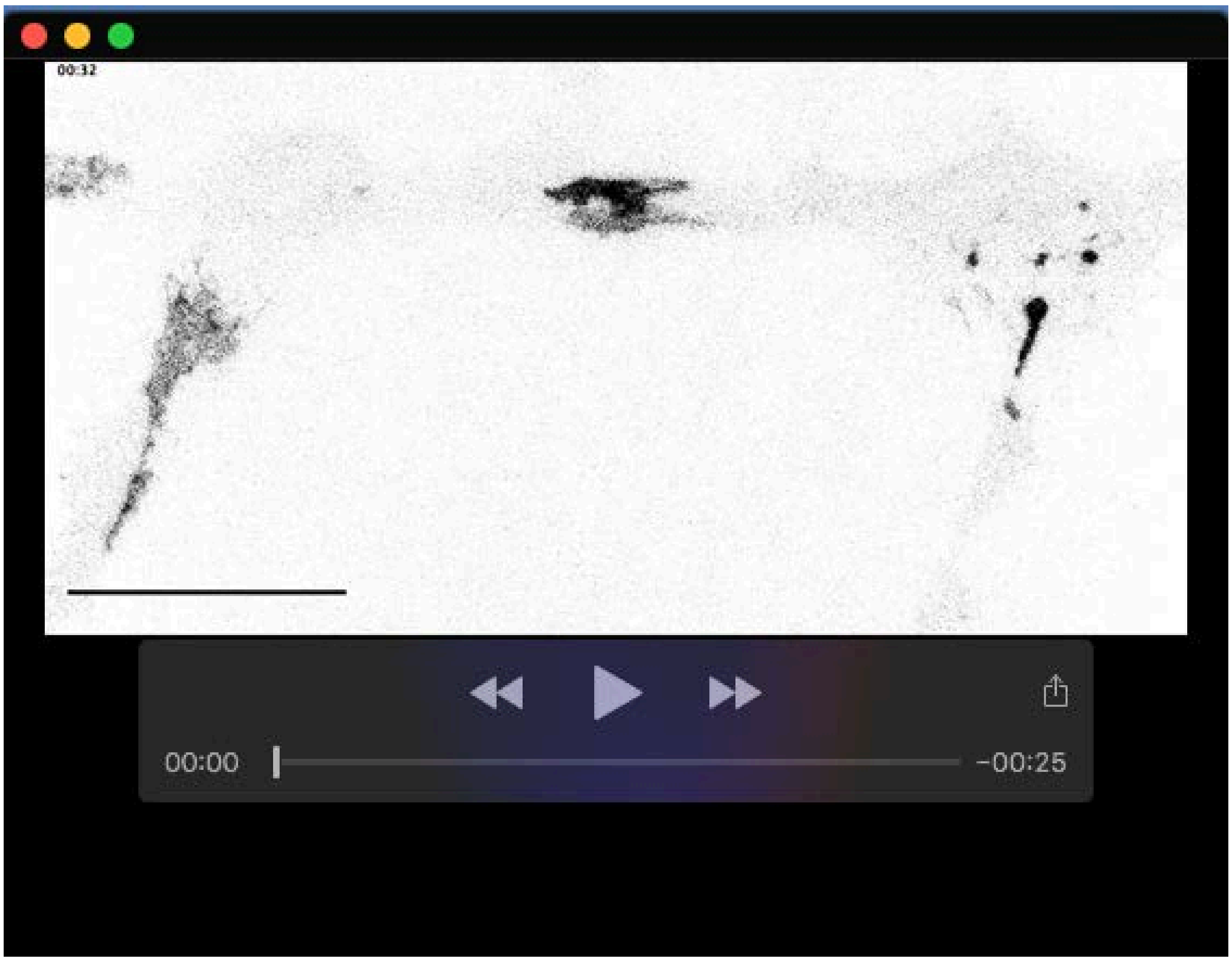

Movie 3 (Main figure 2A-C): Confocal time-lapse movie of anastomotic ring formation in a wild-type and in two *rasip1*^{ubs28} mutant embryos from 32 hpf. Endothelial cell junctions are labeled by VE-cad-Venus (*Tg(cdh5:cdh5-TFP-TENS-Venus)*^{uq11bh}) (reverse contrast). Scale bar, 20 μm.

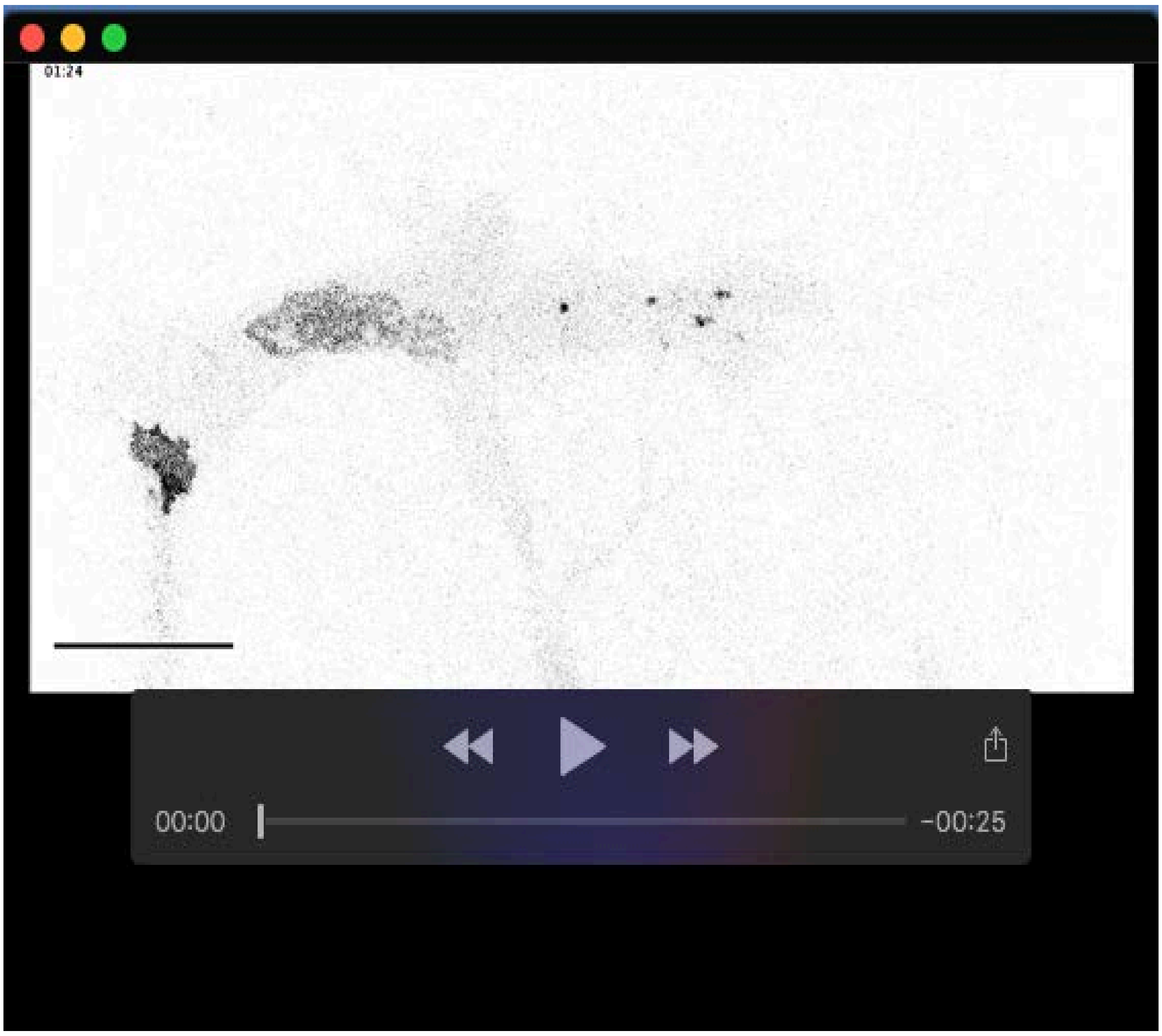

Movie 4 (Main figure 2A-C): Confocal time-lapse movie of anastomotic ring formation in a wild-type and in two *rasip1*^{ubs28} mutant embryos from 32 hpf. Endothelial cell junctions are labeled by VE-cad-Venus (*Tg(cdh5:cdh5-TFP-TENS-Venus)*^{uq11bh}) (reverse contrast). Scale bar, 20 µm.

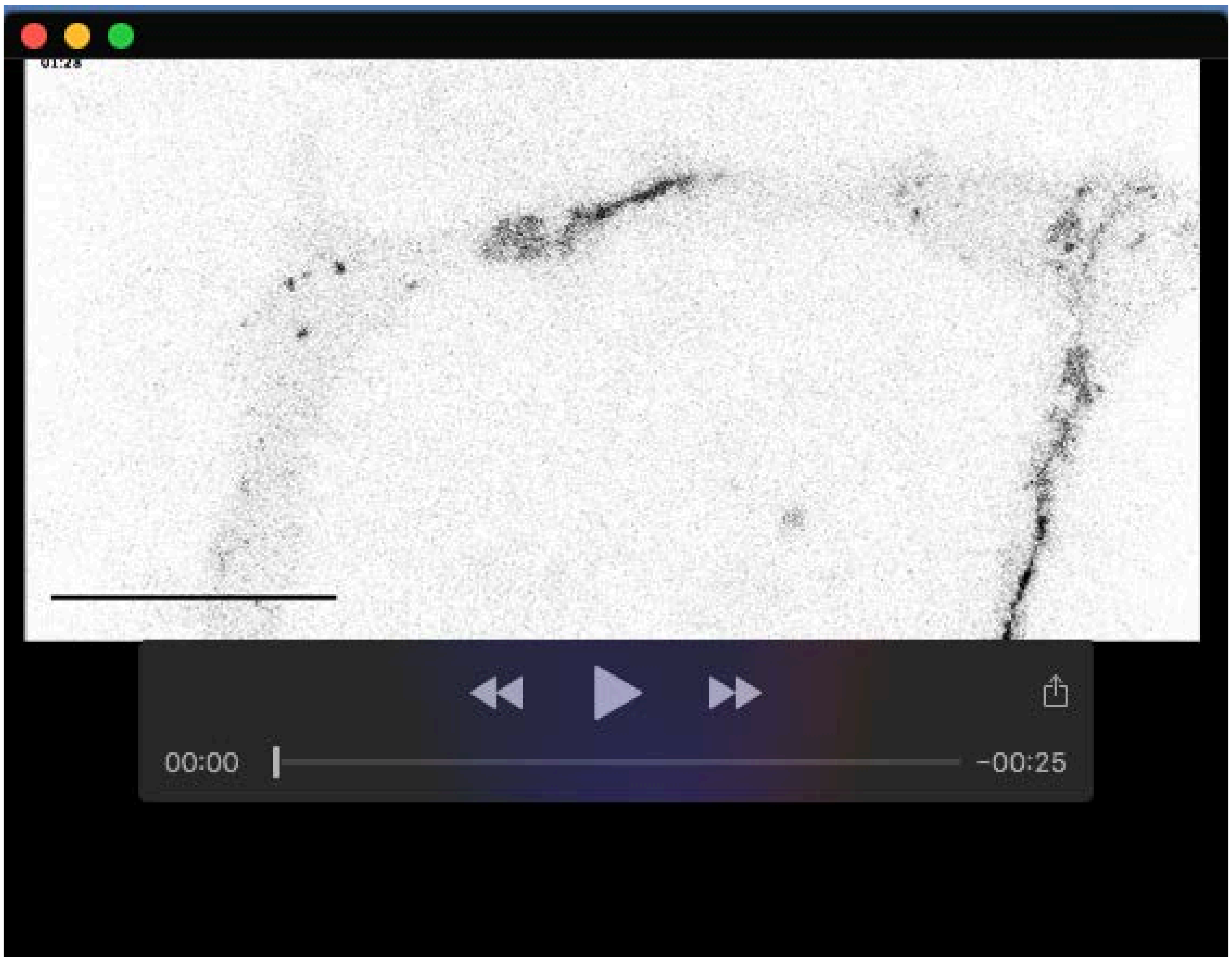

Movie 5 (Main figure 2A-C): Confocal time-lapse movie of anastomotic ring formation in a wild-type and in two *rasip1*^{ubs28} mutant embryos from 32 hpf. Endothelial cell junctions are labeled by VE-cad-Venus (*Tg(cdh5:cdh5-TFP-TENS-Venus)*^{uq11bh}) (reverse contrast). Scale bar, 20 µm.

Development • Supplementary information

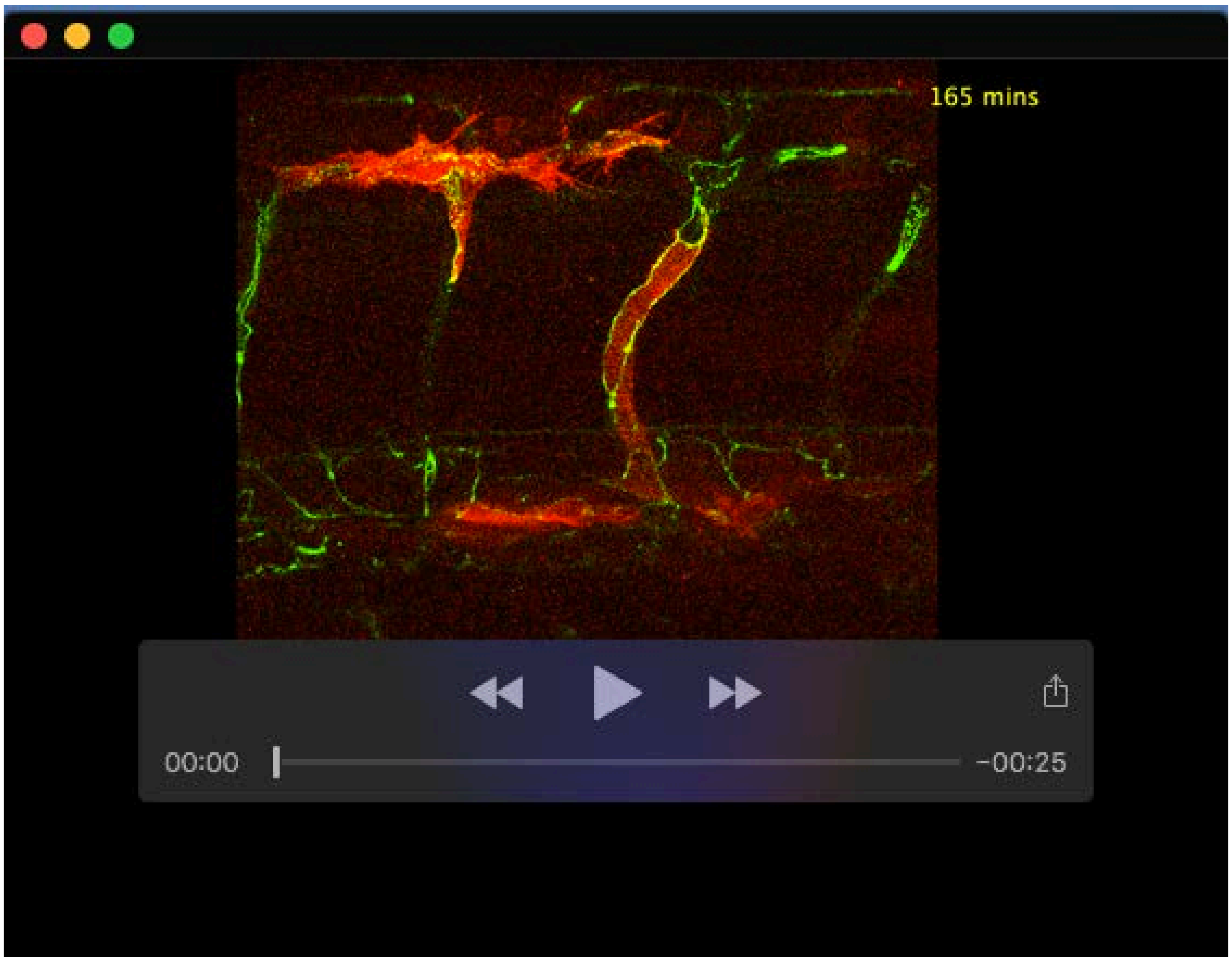

Movie 6 (Main figure 3A): Confocal time-lapse movie showing behavior of transplanted wild-type cells (red) in a *rasip1*^{ubs28} mutant embryo starting at 30 hpf (interval is 5 minute). Endothelial junctions are visualized by EGFP *(Tg(fli1a:pecam1-eGFP)*^{ncv27}*)*.

Development • Supplementary information

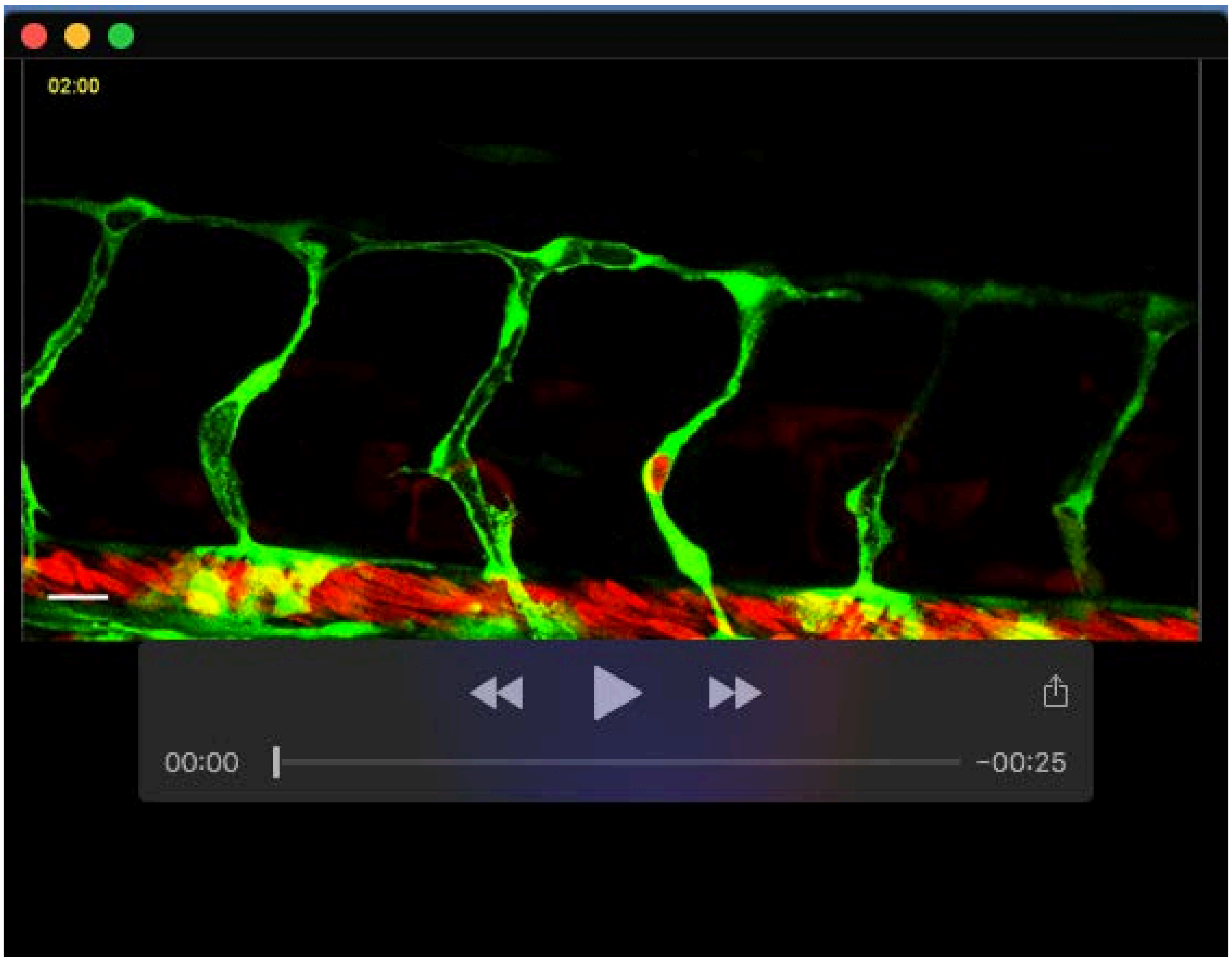

Movie 7 (Main figure 4A): Confocal time-lapse movie showing lumen formation and the onset of blood circulation in a wild-type and in a *rasip1*^{ubs28} mutant embryo starting at 32 hpf. Endothelial cells are labeled by EGFP (*Tg(kdrl:EGFP)*^{s843}; blood cells are labeled by DsRed *Tg(gata1:DsRed)*^{sd2}. Scale bars, 20 µm.

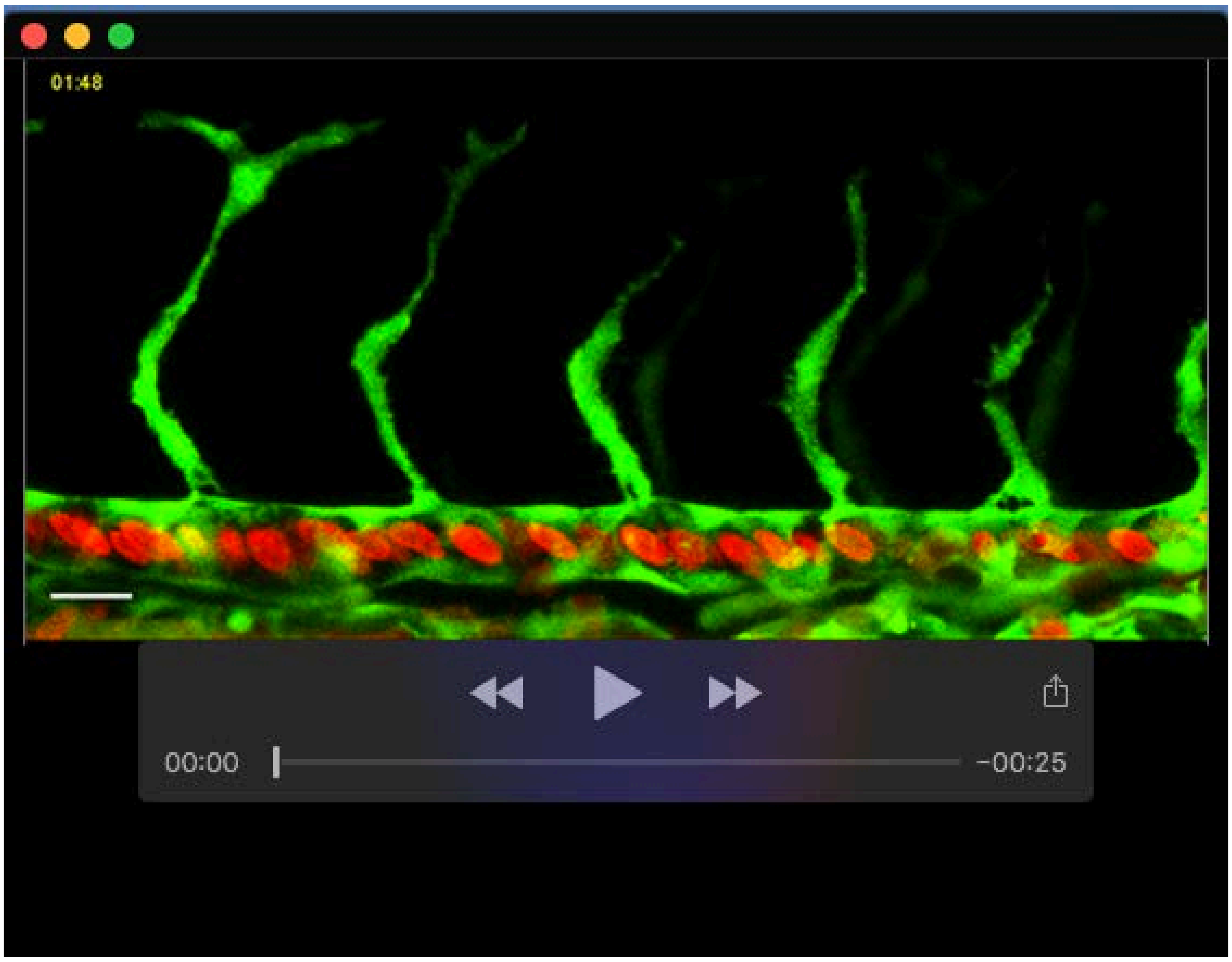

Movie 8 (Main figure 4A): Confocal time-lapse movie showing lumen formation and the onset of blood circulation in a wild-type and in a *rasip1*^{ubs28} mutant embryo starting at 32 hpf. Endothelial cells are labeled by EGFP (*Tg(kdrl:EGFP)*^{s843}; blood cells are labeled by DsRed *Tg(gata1:DsRed)*^{sd2}. Scale bars, 20 µm.

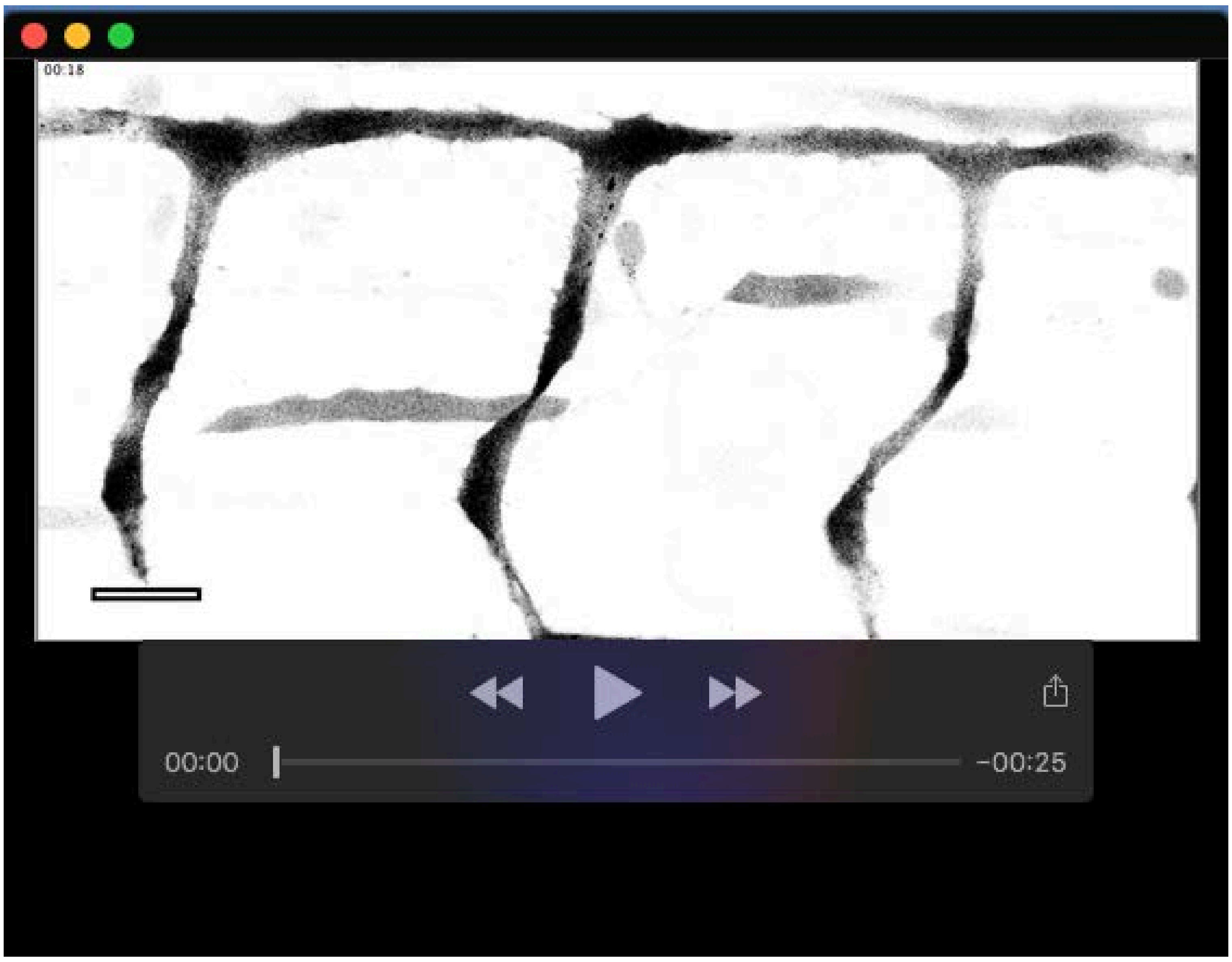

Movie 9 (Main figure 5A): Confocal time-lapse movie of ISV formation (24-30 hpf) in a wild-type and in a *rasip1*^{ubs28} mutant embryo. Endothelial cells are labeled by *Tg(kdrl:EGFP)*^{s843} (inversed contrast). Scale bar, 50 µm.

Development • Supplementary information

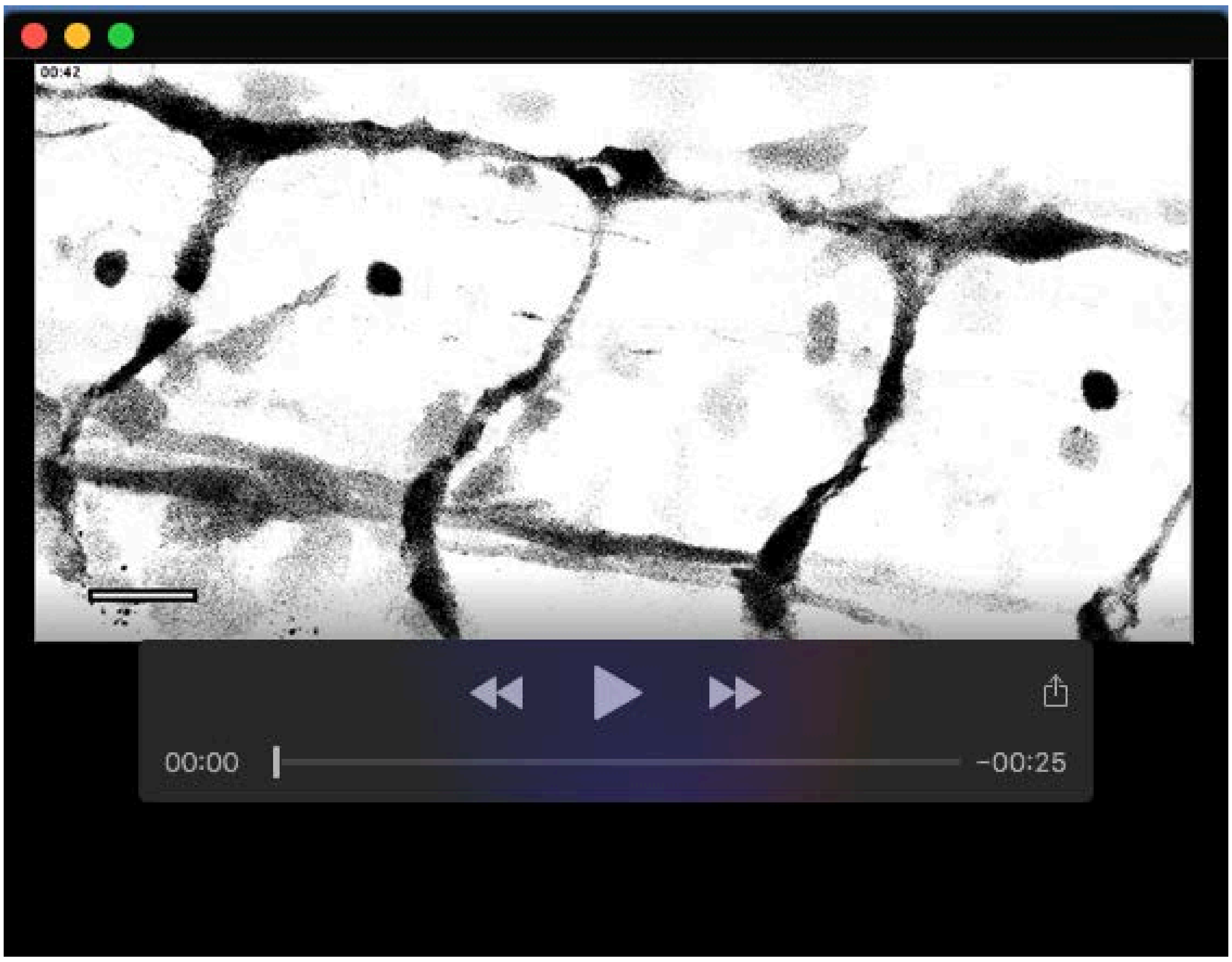

Movie 10 (Main figure 5A): Confocal time-lapse movie of ISV formation (24-30 hpf) in a wild-type and in a *rasip1*^{ubs28} mutant embryo. Endothelial cells are labeled by *Tg(kdrl:EGFP)*^{s843} (inversed contrast). Scale bar, 50 µm.

Development • Supplementary information

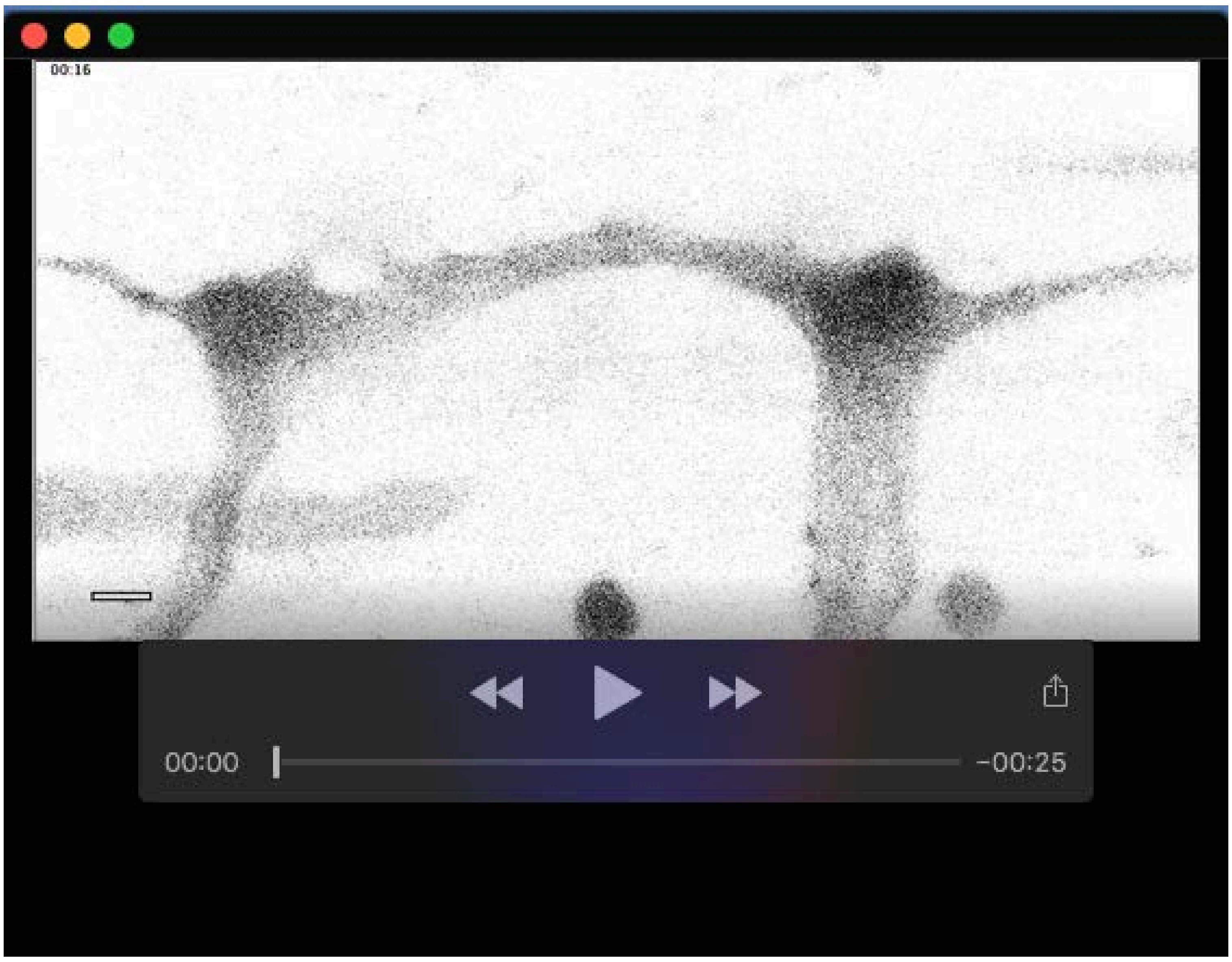

Movie 11 (Main figure 5D): Confocal time-lapse movie showing lumen formation in the ISV and DLAV from 34 hpf onwards in a wild-type embryo. Endothelial cells are labeled by cytoplasmic RFP (*Tg(fli1a:gal4ff)*^{ubs3}*; (UAS:mRFP)*) and endothelial cell junctions in green (VE-cad-Venus) ((*Tg(cdh5:cdh5-TFP-TENS-Venus)*^{uq11bh}).

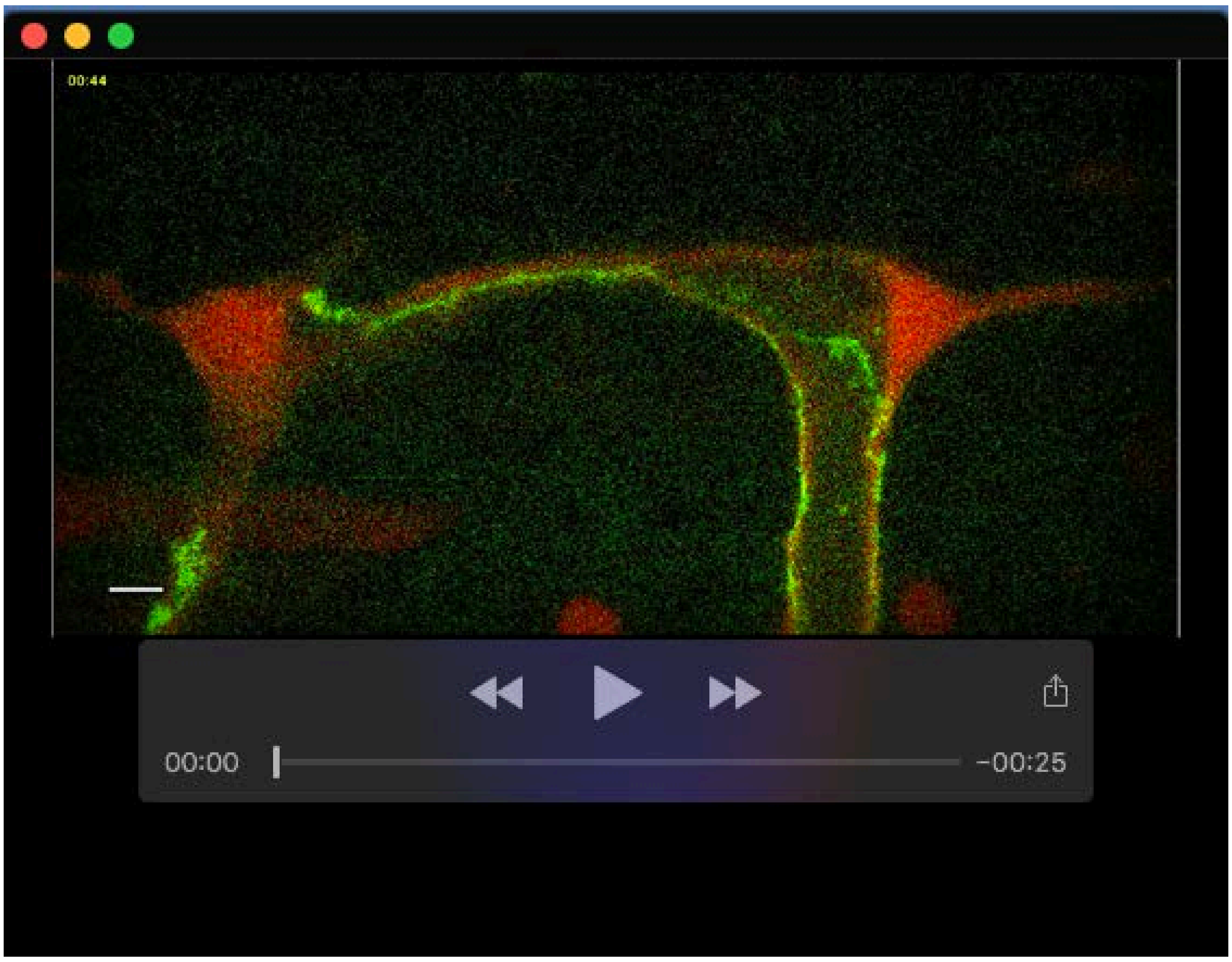

Movie 12 (Main figure 5D): Confocal time-lapse movie showing lumen formation in the ISV and DLAV from 34 hpf onwards in a wild-type embryo. Endothelial cells are labeled by cytoplasmic RFP (*Tg(fli1a:gal4ff)*^{ubs3}*; (UAS:mRFP)*) and endothelial cell junctions in green (VE-cad-Venus) ((*Tg(cdh5:cdh5-TFP-TENS-Venus)*^{uq11bh}).

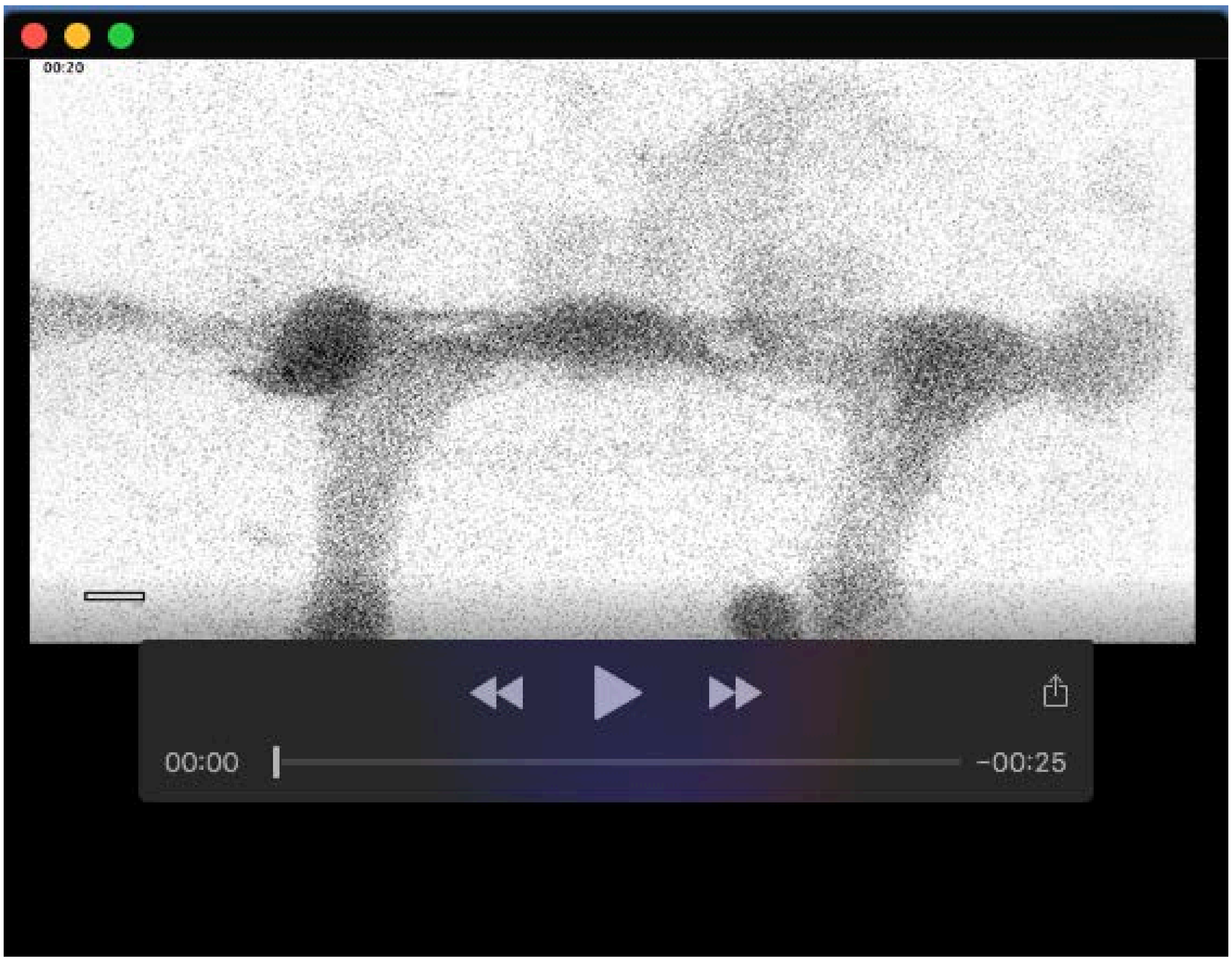

Movie 13 (Main figure 5D): Confocal time-lapse movie showing lumen formation in the ISV and DLAV from 34 hpf onwards in a wild-type embryo. Endothelial cells are labeled by cytoplasmic RFP (*Tg(fli1a:gal4ff)^{ubs3}; (UAS:mRFP)*) and endothelial cell junctions in green (VE-cad-Venus) ((*Tg(cdh5:cdh5-TFP-TENS-Venus)^{uq11bh}*).

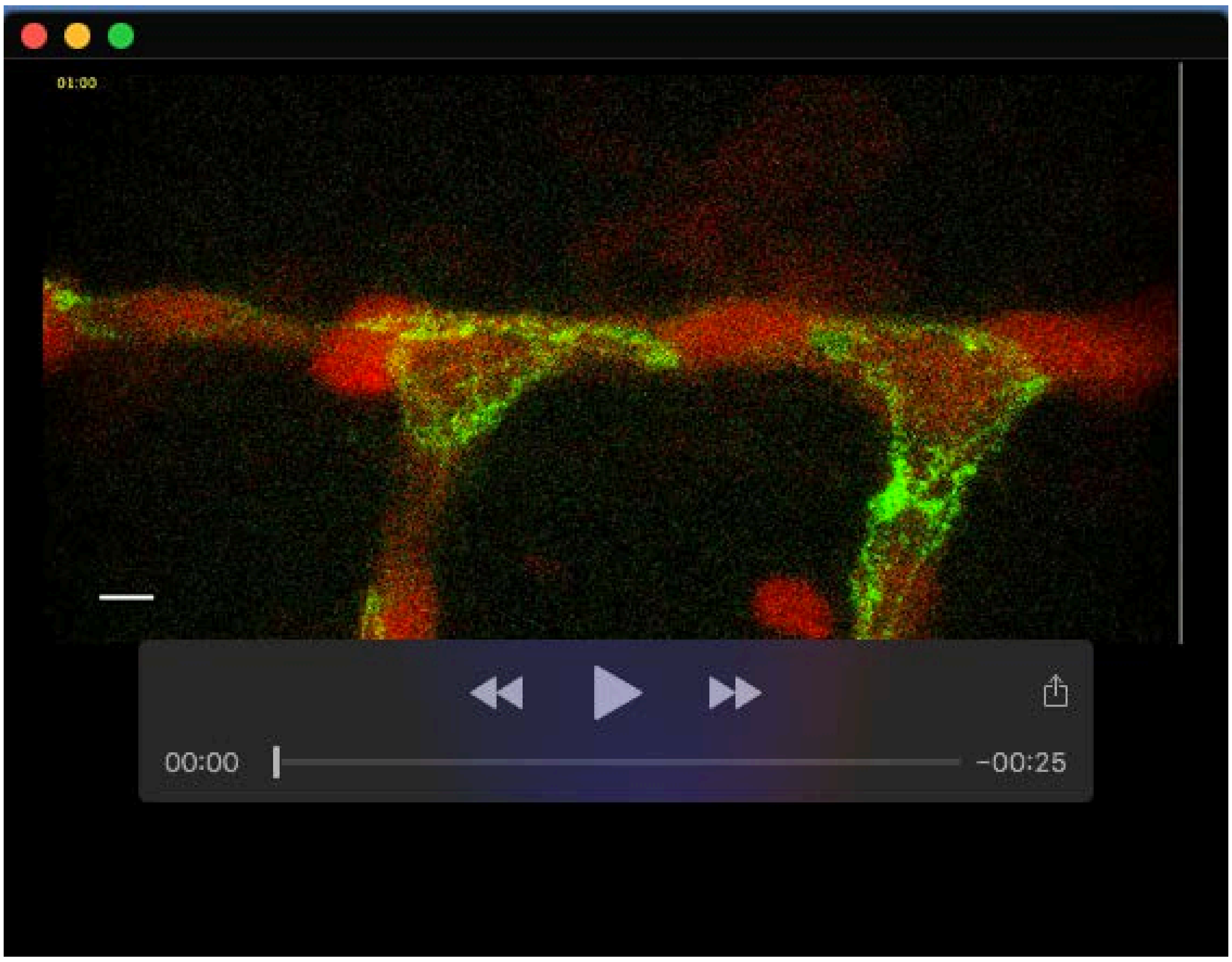

Movie 14 (Main figure 5D): Confocal time-lapse movie showing lumen formation in the ISV and DLAV from 34 hpf onwards in a wild-type embryo. Endothelial cells are labeled by cytoplasmic RFP (*Tg(fli1a:gal4ff)*^{ubs3}*; (UAS:mRFP)*) and endothelial cell junctions in green (VE-cad-Venus) ((*Tg(cdh5:cdh5-TFP-TENS-Venus)*^{uq11bh}).

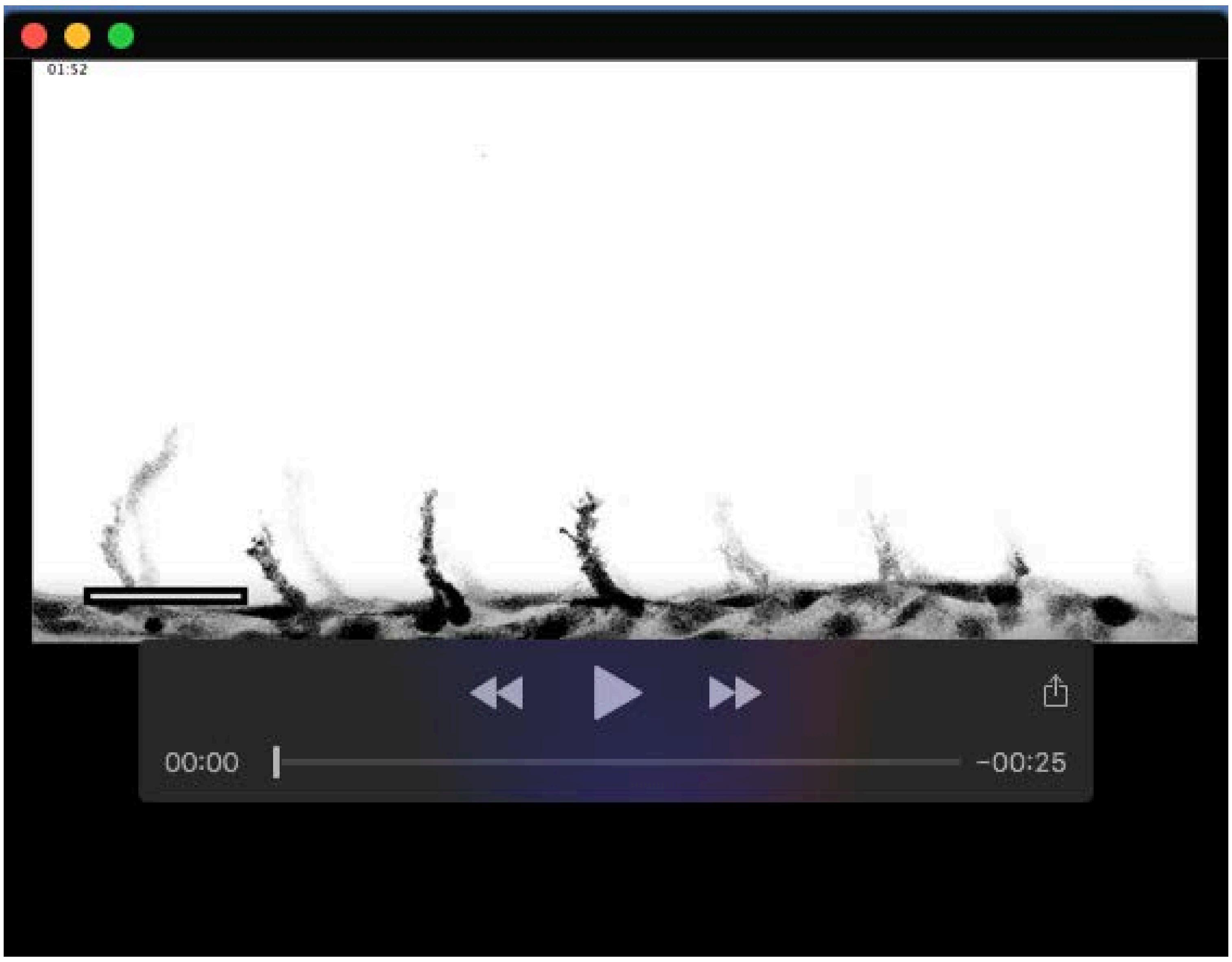

Movie 15 (Supplementary figure 3A): Confocal time-lapse movie of ISV formation (24-30 hpf) in wild-type and *rasip1*^{ubs28} embryos. Endothelial cells are labeled by *Tg(kdrl:EGFP)*^{s843} (inversed contrast). Compared to wild-type, *rasip1*^{ubs28} mutants display unsynchronized and disrupted angiogenetic sprouting. Scale bar, 50 µm.

Development • Supplementary information

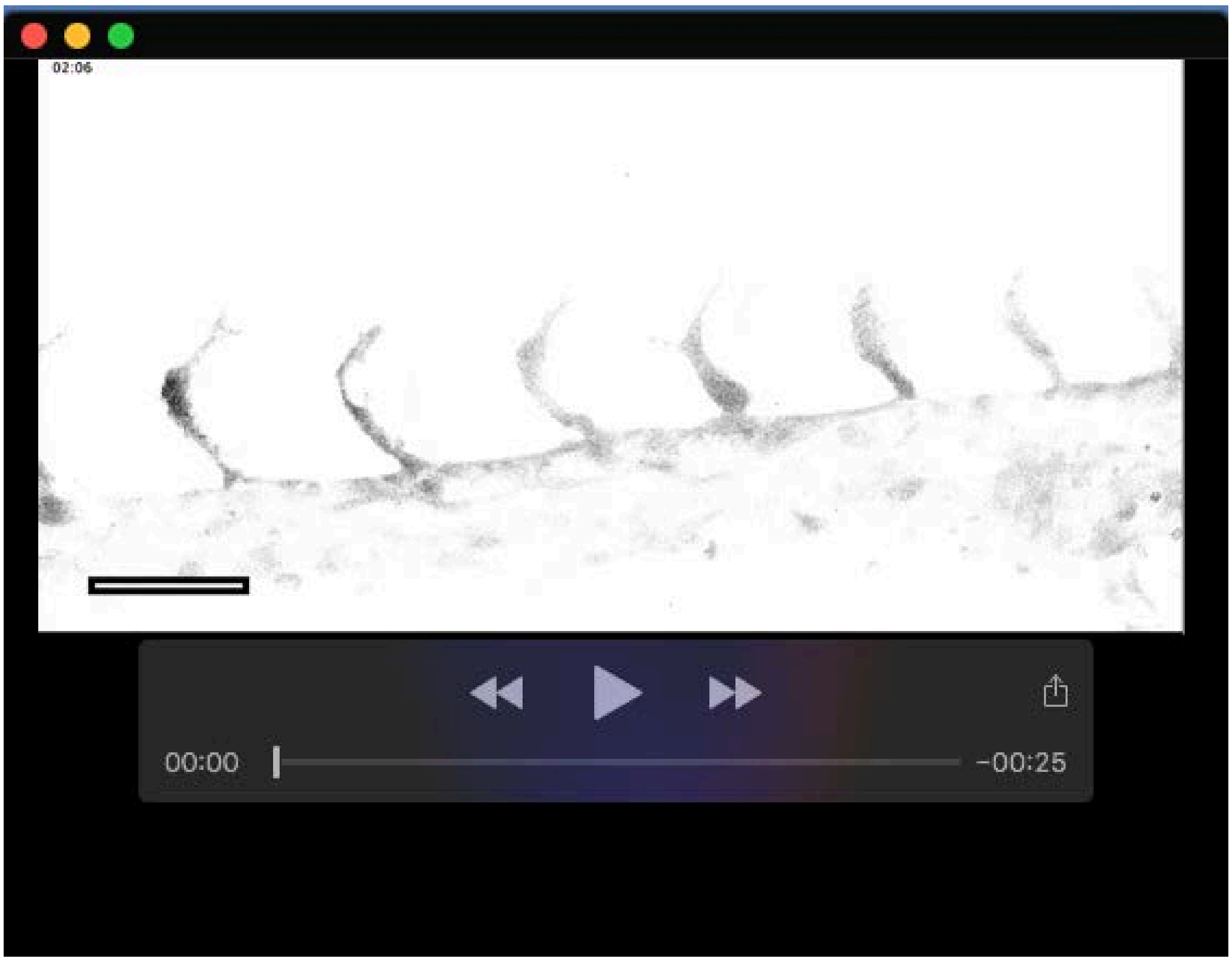

Movie 16 (Supplementary figure 3A): Confocal time-lapse movie of ISV formation (24-30 hpf) in wild-type and *rasip1*^{ubs28} embryos. Endothelial cells are labeled by *Tg(kdrl:EGFP)*^{s843} (inversed contrast). Compared to wild-type, *rasip1*^{ubs28} mutants display unsynchronized and disrupted angiogenetic sprouting. Scale bar, 50 µm.

Development • Supplementary information

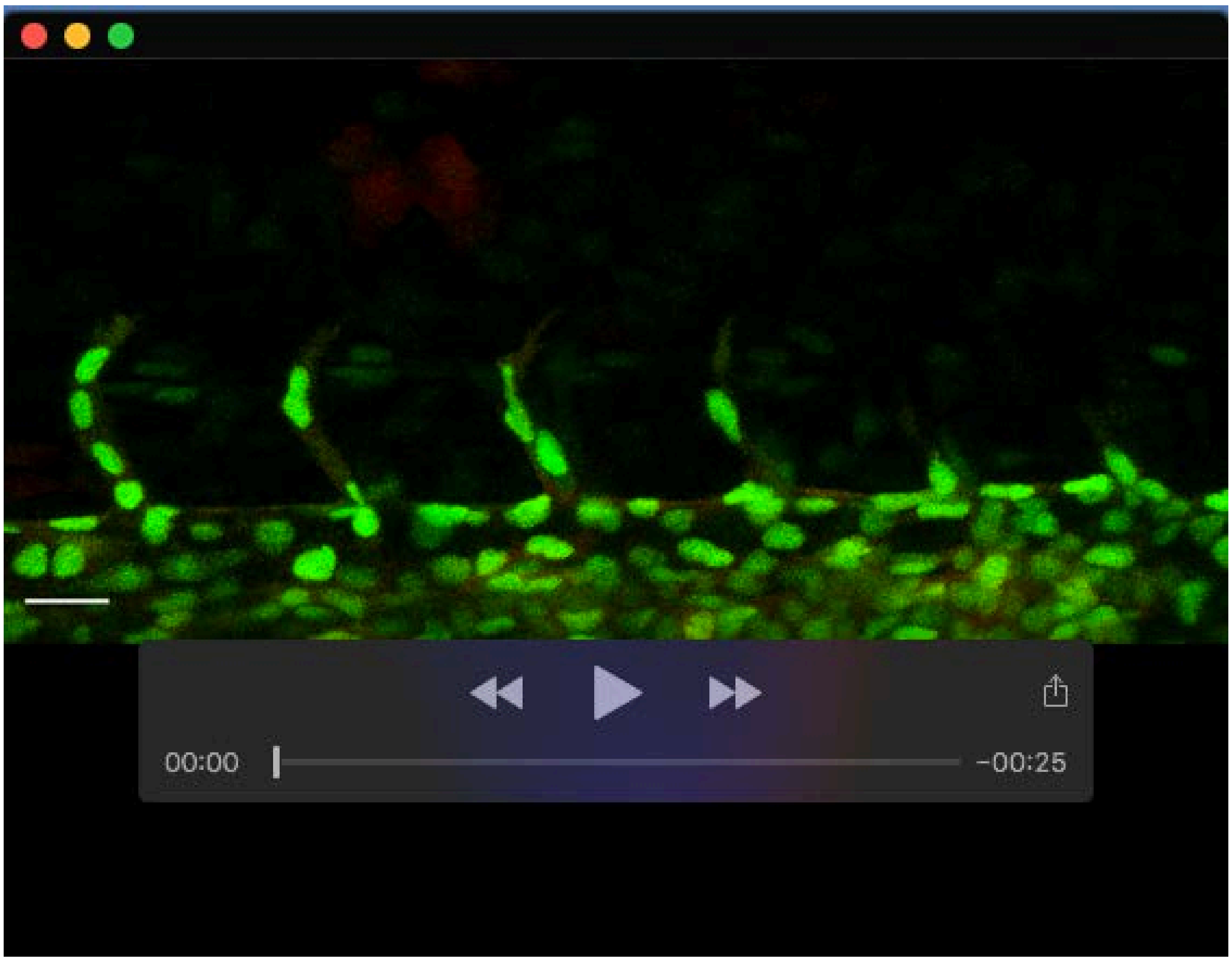

Movie 17 (Supplementary figure 3E,F): Confocal time-lapse movie of ISV formation from 24 hpf in wild-type and *rasip1*^{ubs28} embryos. Endothelial cells are labeled by *Tg(fli1a:gal4ff)*^{ubs3}*; (UAS:mRFP)* in red, and nuclei are labeled by *Tg(kdrl:EGFPnls)*^{ubs1} in green. Scale bar, 20 µm.

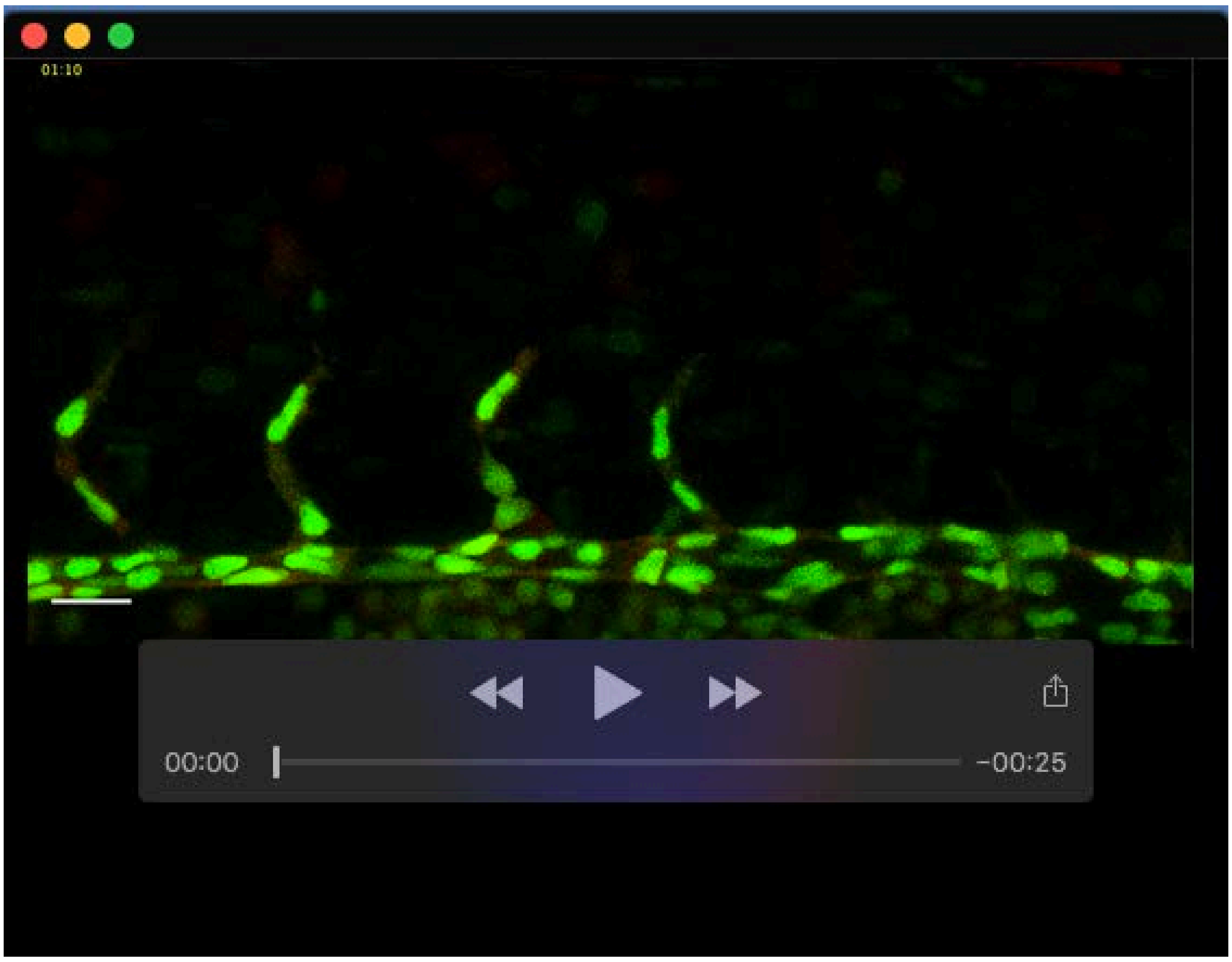

Movie 18 (Supplementary figure 3E,F): Confocal time-lapse movie of ISV formation from 24 hpf in wild-type and *rasip1*^{ubs28} embryos. Endothelial cells are labeled by *Tg(fli1a:gal4ff)*^{ubs3}*; (UAS:mRFP)* in red, and nuclei are labeled by *Tg(kdrl:EGFPnls)*^{ubs1} in green. Scale bar, 20 µm.

Development • Supplementary information

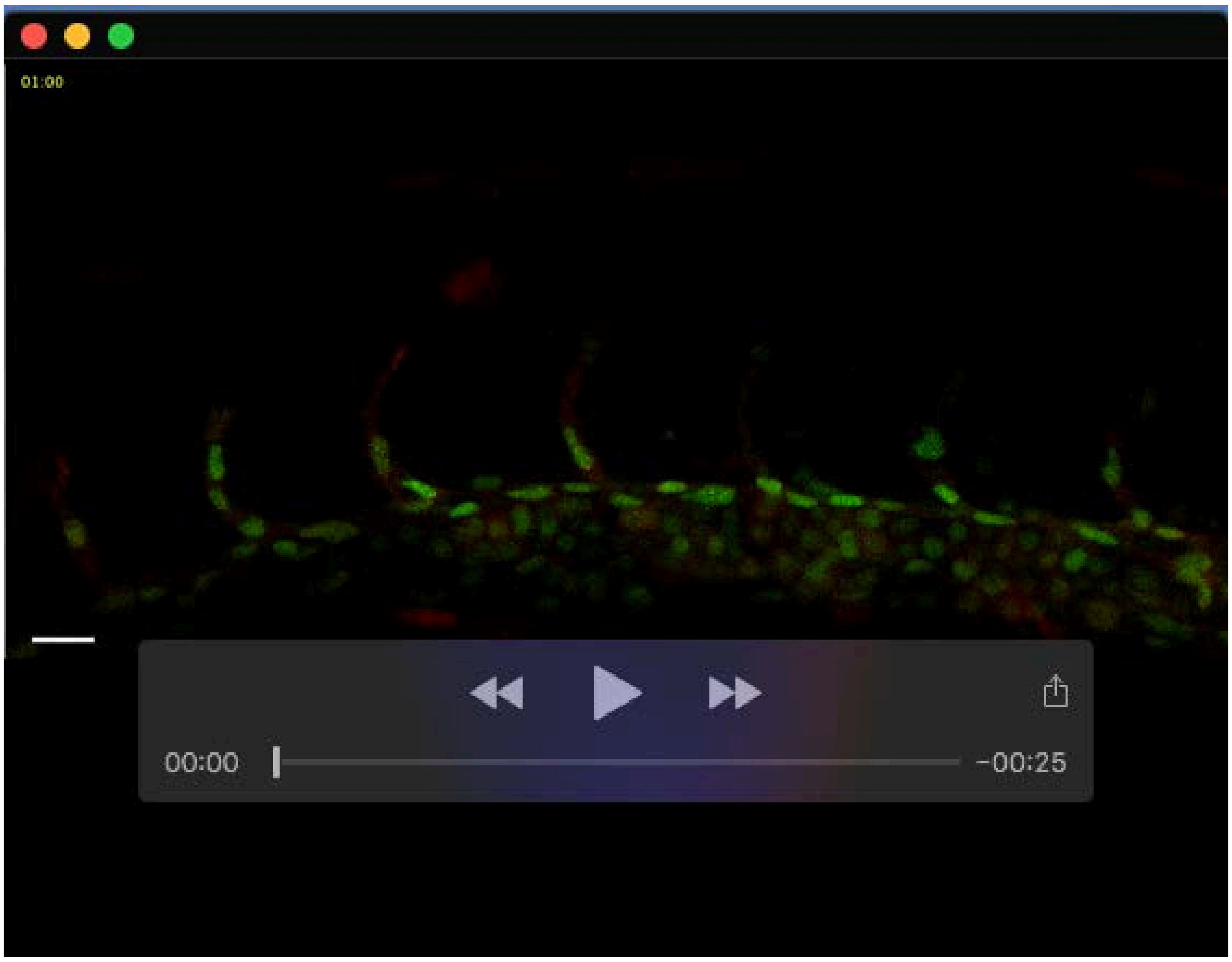

Movie 19 (Supplementary figure 3E,F): Confocal time-lapse movie of ISV formation from 24 hpf in wild-type and *rasip1*^{ubs28} embryos. Endothelial cells are labeled by *Tg(fli1a:gal4ff)*^{ubs3}*; (UAS:mRFP)* in red, and nuclei are labeled by *Tg(kdrl:EGFPnls)*^{ubs1} in green. Scale bar, 20 μm.

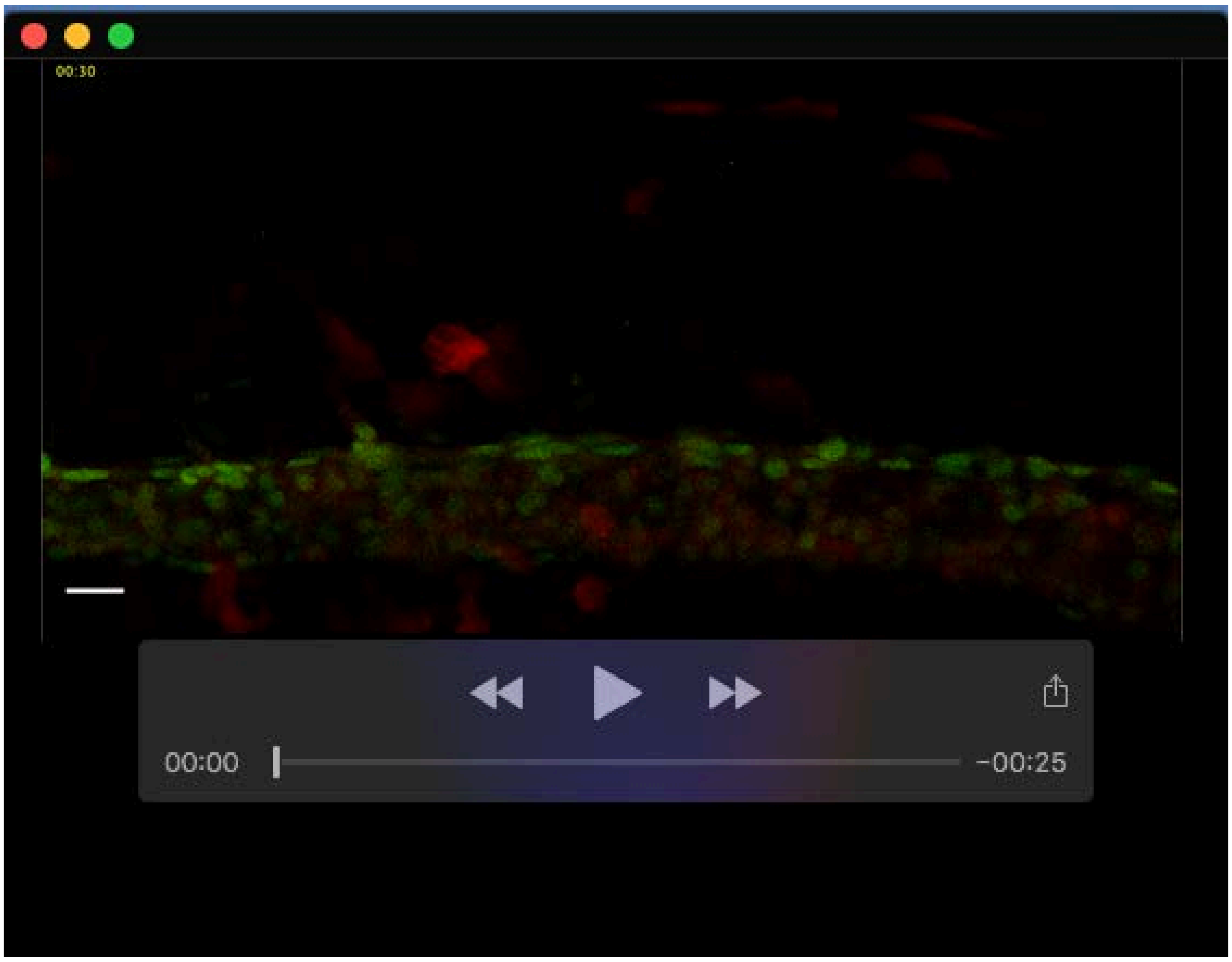

Movie 20 (Supplementary figure 3E,F): Confocal time-lapse movie of ISV formation from 24 hpf in wild-type and *rasip1*^{ubs28} embryos. Endothelial cells are labeled by *Tg(fli1a:gal4ff)*^{ubs3}*; (UAS:mRFP)* in red, and nuclei are labeled by *Tg(kdrl:EGFPnls)*^{ubs1} in green. Scale bar, 20 µm.

Development • Supplementary information

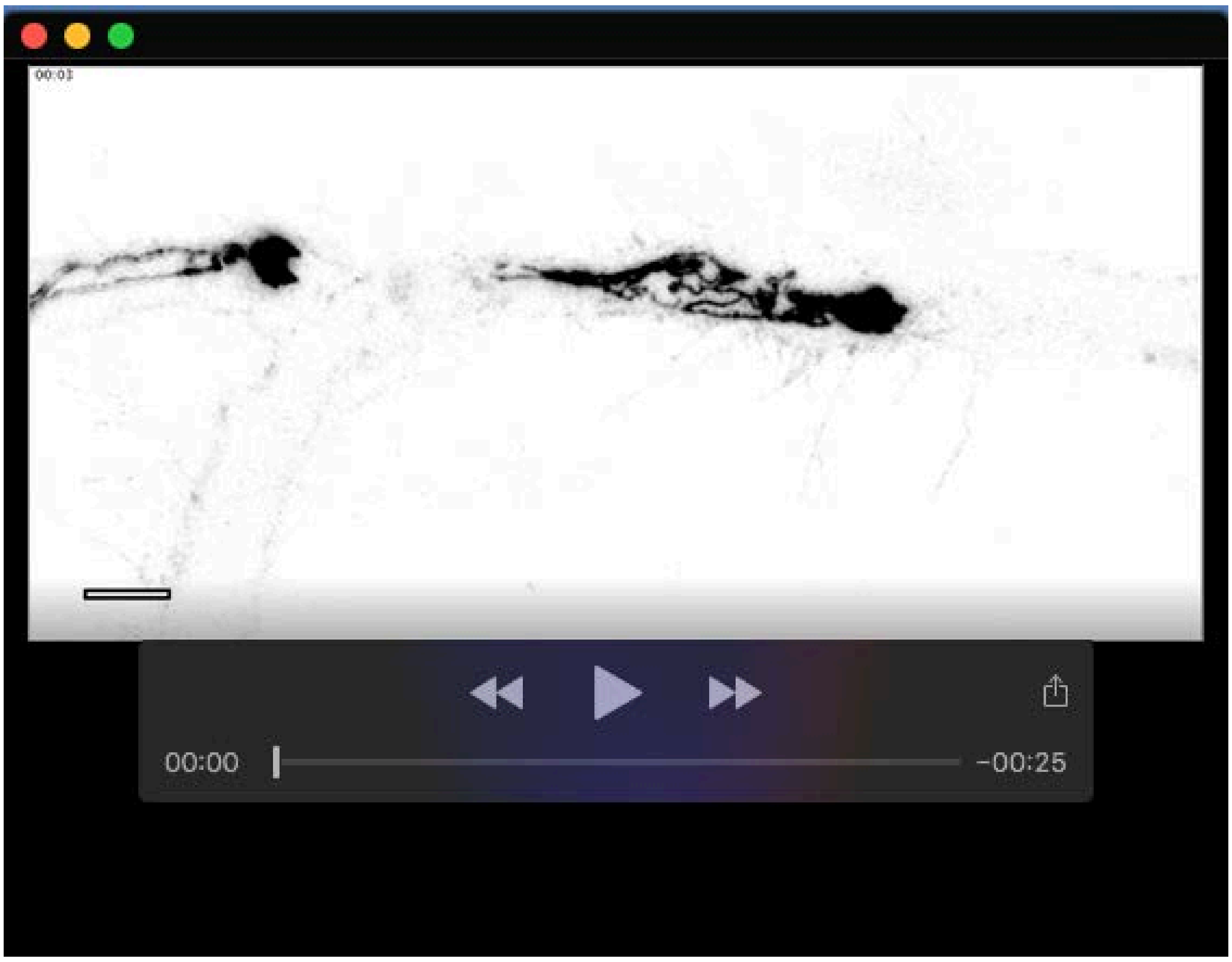

Movie 21 (Supplementary figure 4): Confocal time-lapse movie showing dynamic re-localization of Pecam-EGFP (*Tg(fli1a:pecam1-eGFP)*^{ncv27}) during anastomosis in a wild-type and in a *rasip1*^{ubs28} embryo, starting at 32 hpf and recorded at 1 frame/min (00:00 to 00:43). In the *rasip1*^{ubs28} mutant, a defect in the localization of apical junctional proteins was observed. Scale bars, 5 µm.

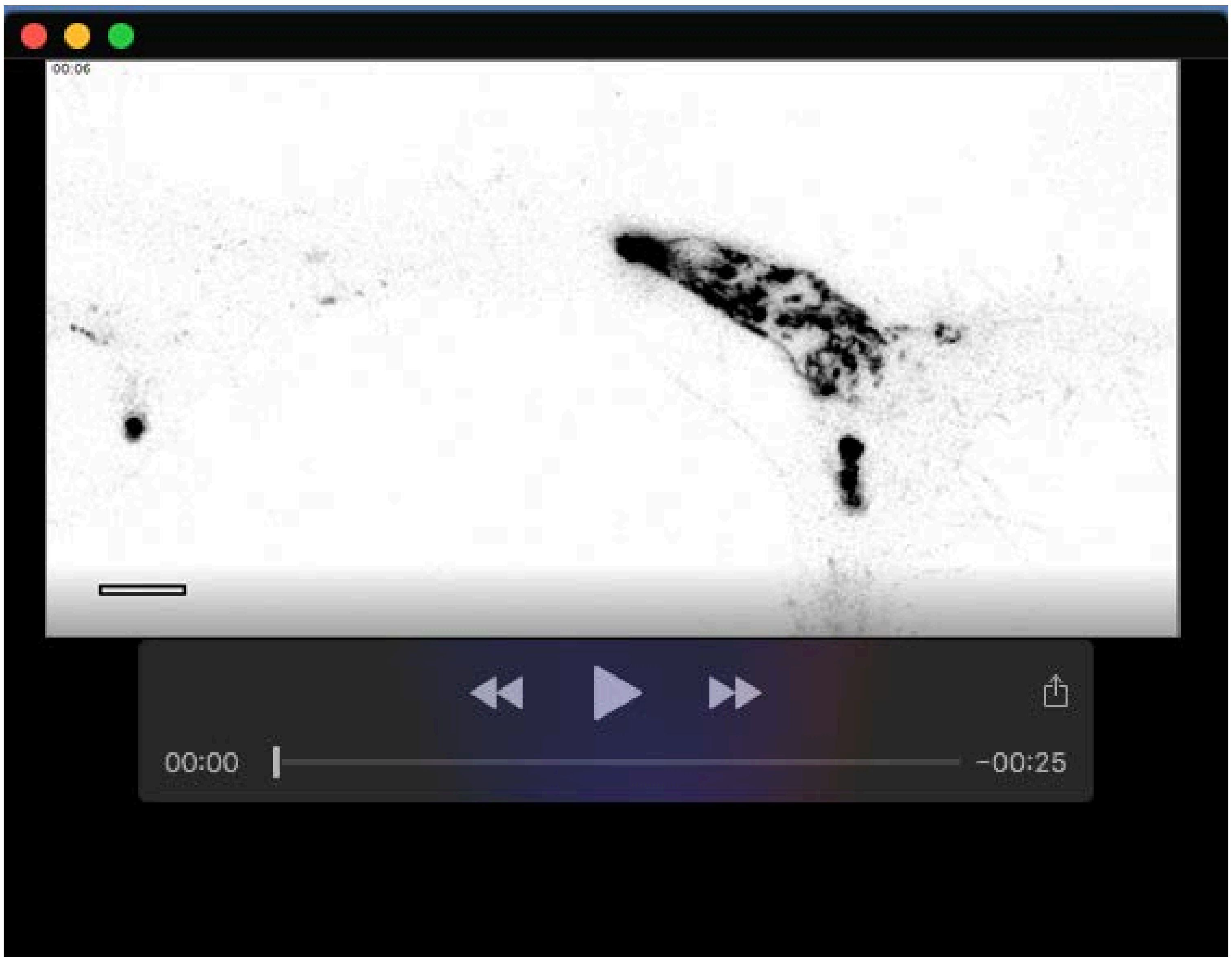

Movie 22 (Supplementary figure 4): Confocal time-lapse movie showing dynamic re-localization of Pecam-EGFP (*Tg(fli1a:pecam1-eGFP)*^{ncv27}) during anastomosis in a wild-type and in a *rasip1*^{ubs28} embryo, starting at 32 hpf and recorded at 1 frame/min (00:00 to 00:43). In the *rasip1*^{ubs28} mutant, a defect in the localization of apical junctional proteins was observed. Scale bars, 5 µm.
