# Supplementary material for: Control of dynamic cell behaviors during angiogenesis and anastomosis by Rasip1
Source: Development. 2021 Aug 12;148(15):dev197509. doi: 10.1242/dev.197509 (PMC8380458; doi:10.1242/dev.197509)
Supplement: Reviewer comments [file develop-148-197509_review_history.pdf]

Control of dynamic cell behaviors during angiogenesis and anastomosis by Rasip1

Minkyoung Lee, Charles Betz, Jianmin Yin, Ilkka Paatero, Niels Schellinx, Adam N. Carte, Christopher W. Wilson, Weilan Ye, Markus Affolter and Heinz-Georg Belting

DOI: 10.1242/dev.197509

Editor: Steve Wilson

Review timeline

Original submission:	6 October 2020
Editorial decision:	23 November 2020
First revision received:	4 May 2021
Accepted:	8 June 2021

Original submission

First decision letter

MS ID#: DEVELOP/2020/197509

MS TITLE: Control of dynamic cell behaviors during angiogenesis and anastomosis by Rasip 1

AUTHORS: Minkyoung Lee, Charles Betz, Ilkka Paatero, Niels C Schellinx, Jianmin Yin, Christopher W Wilson, Weilan Ye, Markus Affolter, and Heinz-Georg Belting

I have now received all the referees' reports on the above manuscript, and have reached a decision. The referees' comments are appended below, or you can access them online: please go to BenchPress and click on the 'Manuscripts with Decisions' queue in the Author Area.

As you will see, the referees express considerable interest in your work, but have some significant criticisms and suggestions for improving your manuscript. In comments to the editor, both referees 1 and 3 expressed the opinion that while the quality of the data you present is very good, the level of advance in understanding how Rasip functions is marginal and that further experimental work could significantly strengthen the study. If you are able to revise the manuscript along the lines suggested, I will be happy receive a revised version of the manuscript. Your revised paper will be re-reviewed by one or more of the original referees, and acceptance of your manuscript will depend on your addressing satisfactorily the reviewers' major concerns.
Please also note that Development will normally permit only one round of major revision.

We are aware that you may be experiencing disruption to the normal running of your lab that make experimental revisions challenging. If it would be helpful, we encourage you to contact us to discuss your revision in greater detail. Please send us a point-by-point response indicating where you are able to address concerns raised (either experimentally or by changes to the text) and where you will not be able to do so within the normal timeframe of a revision. We will then provide further guidance. Please also note that we are happy to extend revision timeframes as necessary.

Please attend to all of the reviewers' comments and ensure that you clearly highlight all changes made in the revised manuscript. Please avoid using 'Tracked changes' in Word files as these are lost in PDF conversion. I should be grateful if you would also provide a point-by-point response detailing how you have dealt with the points raised by the reviewers in the 'Response to Reviewers' box. If you do not agree with any of their criticisms or suggestions please explain clearly why this is so.

© 2021. Published by The Company of Biologists under the terms of the Creative Commons Attribution License (https://creativecommons.org/licenses/by/4.0/).

Reviewer 1

Advance summary and potential significance to field

The manuscript describes the phenotpyic consequences of mutations in rasip and radilb in zebrafish embryos. The data provide a carefully executed description of lack of Rasip and on junctional integrity at the ISV level, and suggest a number of ways how the respective proteins could exert their effect. Through comparison with ccm1 and heg1 morphants , which exhibit similar phenotypes, a case is made for a possible interaction of these proteins. The study confirms previous work in the mouse (Barry et al 2016) about the importance of Rasip, and will be of interest to readers in the field of vessel morphogenesis and junctional integrity.

Comments for the author

(1) the abstract reads a little choppy, which is in part due to the fact that not all of the experimental lines are logically connected among each other.

(2) The rasip mutants develop early edema, exhibit severe brain hemorrhaging, and show a phenotpye in ISV sprouting that is most prominent early, but appears to sort itself out over time (72hpf). Some of these phenotypic features raise the issue of systemic effects, and whether mutants suffer from a general delay. Cell transplantation of mutants cells into wild type vessels would demonstrate a cell autonomous effect of lack of Rasip, and at least partially adress the issue of a possible systemic effect in rasip mutants.

(3) rasip mutants show a dramatic hemorrhaging phenotype, but the authors restrain themselves to one panel in figure 1C. Have the authors checked whether the same junctional defects described in the trunk are causative for the brain vasculature defects?

(4) rasip mutants are reported to exhibit reduced blood flow - has this been quantified, and does this get fully restored at 72hpf?

(5) rasip mutants show reduced endothelial cell proliferation at the level of the dorsal aorta - can the authors speculate how this is brought about? In the discussion the obvious is stated ('fewer cells reflect a reduced rate of cell proliferation'), but could this be an effect of altered lumen formation or reduced blood flow as well?

(6) Rasip function has been analyzed previously. While the data presented in figs 2,3,5,6,7 of the present manuscript are certainly interesting, they do not necessarily provide a step advance in our understanding of junction formation and junction stability, or of developmental angiogenesis. One understands better the effects of Rasip loss of function in trunk vessels, but the scope of the work appears somewhat narrow. Data on radil-b and heg1/ccm1 add little to the main story line of the manuscript.

Reviewer 2

Advance summary and potential significance to field

The study from Lee et al reports the generation and characterization of vascular morphogenesis in Rasip1 and Radil-b loss of function zebrafish. A previous study using mouse models has identified that Rasip1 is essential for lumen formation (Barry et. al. 2016). This mouse study uncovered an underlying mechanism whereby Rasip1 prior to lumenisation is essential to clear adhesion sites and allow for the initial single lumen to arise between neighboring endothelial cells. Later during lumen formation, Rasip1 also permits for a certain level of actomyosin relaxation (by inhibiting RhoA) allowing lumen expansion.

Here, a zebrafish radil1 mutant model has been used to study endothelial cell behavior along with endothelial junction dynamics by high quality live imaging. In addition, the authors have carefully analysed other morphogenesis events that are driven by cellular rearrangements and essential for the establishment of a functional vascular network, like ISV formation and anastomosis. The results

© 2021. Published by The Company of Biologists under the terms of the Creative Commons Attribution License (https://creativecommons.org/licenses/by/4.0/).

indicate that Rasip1 supports cellular rearrangements in the ISVs and is also essential for clearance of adhesion molecules during the initial stages of anastomosis. This extensive functional analysis of Rasip1 is complemented by the first in vivo analysis of Radil function in the endothelium. Radil is a close orthologue of Rasip1 and has been shown to function redundantly in cultured ECs. Importantly, the work described here reveals overlapping but also distinctive roles for Rasip1 and Radil during vessel network formation.

Comments for the author

While there are some issues that need to be addressed (see below), the study should be well suited for publication.

1. In Figure 1 it appears that there is a higher abundance of ve-cad in the rasip1 mutant embryos. Rasip1 has also been shown to clear adhesion molecules during mouse lumen formation. A similar phenomenon was observed here in zebrafish whereby Rasip1 is essential to clear adhesion molecules during anastomosis. Together this might suggest that Rasip1 more generally is required for ve-cad recycling which in turn would also lead to the compromised cell dynamics identified in the ISVs. Have the authors considered this and examined the differences in ve-cad levels more broadly?

2. It is intriguing to note that there are transient defects in cardiac function and thus blood circulation. Do the authors have an idea why a certain percentage of the mutants can survive whilst others do not?
Interestingly, the vasculature in radilb mutants is described as being less severely affected and that in these embryos also flow is less compromised. Taking into account these observations, how does the reduction in flow pressure actually contribute to some of the earlier phenotypes that have been described?
For example, lumenisation of the ISVs is initiated at 4 dpf but this is also when circulation is described to recover. Do the vessels in the head also lumenise at this later stage? Do other phenotypes like the cellular rearrangements in the ISVs and ISV detachment also recover? How much does flow impact on anastomosis and clearance of adhesion proteins during this process? Can the authors compare their results to a scenario whereby flow is temporarily reduced or lost? It would be interesting to examine the capacity of the vessel network to reconnect and recover by doing a temporal analysis of a viable radil1 mutant. Embryo matched images taken at 2days and 6-7days would reveal if a compromised network re-establishes or whether the embryos are simply viable whilst lacking several ISVs.

3. The authors show that there are additional cells from the dorsal aorta that migrate into the ISVs to compensate for reduced cell division during sprouting angiogenesis. Does this phenomenon have an effect on dorsal aorta integrity and cell number?

4. The authors further explore the relevance of Heg in Rasip1 function. Heg is proposed to recruit Rasip1.
Have the authors examined whether Rasip1 is indeed mis-localised in heg morphants? This would really support the idea that heg loss of function could produce a phenocopy of rasip1 vascular defects.

Minor:

1. There is no reference to the movie numbers in the main text and the movies also do not have a descriptive name so it is not clear to figure out precisely which movie is being discussed in the main text.
This information can only be found in the Supplementary movie legends.

2. From the temporal analysis of Rasip1 protein expression in Sup Fig 4 the authors propose that Rasip1 becomes downregulated later during development and therefore might not be essential for vessel maintenance. It appears however that Rasip1 during the investigated timecourse becomes more localised/junctional? This does not necessarily mean that it is less relevant at the later stages. Perhaps the authors can rephrase these conclusions.

© 2021. Published by The Company of Biologists under the terms of the Creative Commons Attribution License (https://creativecommons.org/licenses/by/4.0/).

3. At 24hpf (Sup Fig 4a) there should already be sprouts that have migrated halfway the trunk and at 28hpf these sprouts would have reached the top (Sup Fig 4b). Presumably the authors have mis-labeled the images represented here. The authors should correct the text and the labelling of this figure to the representative developmental time points.

4. In Figure 6 the authors indicate that Rasip1 does not co-localise with VE-cadherin at initial contacts that lead up to anastomosis. However, since this is fixed tissue it can not be concluded that this VE-cadherin positive contact is the stable contact that would go on to anastomose. Would it be possible that Rasip1 could localise to the actual stable contacts that go on to anastomose?

Reviewer 3

Advance summary and potential significance to field

The manuscript of Lee at al., describes the blood vascular defects associate with the loss of function of Rasip1 (Ras-interacting protein 1), an endothelial-specific regulator of junctional dynamics in blood vessel formation. The authors report morphological defects in both the brain and the trunk vessels. Using static and live imaging they report that Rasip1 is required for tricellular junctions during the formation of ISV. Rasip1 cooperates only partially with other proteins in multimeric complexes consisting of Rap1, Rasip1, Radil. Based oh phenotype similarity Rasip1 seems to interact with Ccm1 and Heg 1 during anastomosis formation.

The imaging work presented in this manuscript is incredibly detailed and beautifully represented as it is typical for Dr. Belting work.
My comments are listed below. Overall the conceptual organization of the data makes the manuscript difficult to follow. For example, the initial description of some of the catastrophic brain and aorta diameter defects makes the data presented afterward difficult to interpret as direct effect of Rasip1 loss of function. For example, how much of ISV and anastomosis defect consequence from a secondary lack of aorta lumen formation, blood flow or hemorrhages?

Comments for the author

More in detail:

1) I would suggest moving the figure 1 as supplemental information as these phenotypes were previously reported and the generation of the mutant can be described in the methods.

2) It is interesting that Rasip1-/- defects are transient. Do the author work with MZ embryos? Can the author study or discuss of why this is the case?
For example, do they think that Rasip1 is only required for early vascular development and other mechanism will be in place later on? Is this phenomenon the result of genetics compensation or more interestingly Rasip1-/- are reveling new molecule(s) that function similarly?

3) It is unclear how the author defines the mutant, some time is called rasip1ubs28 and some other Rasip1 mut. Are these definitions refering to homozygous mutants? Did the author explore the phenotype in the het model? Could it be that the het have less major vascular phenotypes, while still have ISV tube formation defect?

4) It is difficult to interpret the ISV and anastomosis defect while the mutant has early DA defect. Can the author perform transplantation experiment to assay the formation of ISV by mutant cell in a WT DA?

5) Could it be that the proliferation defects maybe the primary cause of the ISV defect? Beside counting the nuclei I would suggest to perform an Edu staining to assay whether endothelial cells are less proliferative in general.

6) Is the function of Rasip1 is the ISV lumen formation different from what expected based on the previous studies e.g Barry et al., 2016. It is difficult to point out from the paper what is new about the authors claim.

© 2021. Published by The Company of Biologists under the terms of the Creative Commons Attribution License (https://creativecommons.org/licenses/by/4.0/).

7) Interestingly the VE-cad-Venus has a different intensity between WT and Rasip1-/- (Fig. 2a). Is this representative of the results? Do the mutant have more vecad expression or larger adherent junctions?

8) Can some of the defects observed in the ISV explained by the lack of flow? Can the author rescue this phenotype by expressing Rasip1in endothelial cells? The hemodynamics defect likely plays a role in both lumen formation and maintenance. It is critical to distinguish whether these defects are the direct result of lacking Rasip1 or a combination of the two.

9) Figure 6 showed the Rasip1 endogenous protein expression within the ISV. Can the author provide a quantification to prove the expression or localization changes that they clame?

10) None of the data provide in figure 7 are quantified. It is difficult to conclude that the effects of Rasip1 and Radil-b mutant alone or in combination are significant. Quantifications are required in all the genotypes for each ISV and anastomosis defect to further conclude the cooperation between Rasip1 and Radil-b.

11) It is difficult to conclude or even suggest that Rasip1 might cooperate with Heg1 and CCM1 based solely on the comparison between phenotype. More detail epistasis and possibly more molecular experiments are needed to prove this hypothesis.

First revision

Author response to reviewers' comments

Response to reviewer comments

Reviewers' comments in plain font, author reply in green
We thank the reviewers for their overall positive responses and appreciate their constructive criticism, which has helped us greatly to improve our study. In this revised version we have added new data. As requested by all three reviewers we have expanded our characterization of the *heg1/cm1* morphant phenotypes and show that Rasip1 is delocalized from EC junctions in these morphants. As requested by reviewers 1 and 3, we have addressed the question whether the vascular defects in *rasip1* mutants are autonomous (pleiotropic) or systemic by cell-transplantation and transient overexpression of *rasip1* in *rasip1* mutants. Overall, these experiments show that endothelial expression of Rasip1 is sufficient to rescue *rasip1* mutant phenotypes and that Rasip1 is required autonomously during blood vessel morphogenesis. We have also reorganized some of the figures to better present our data.

Below, please find our point-by-point responses to the reviewer’s comments.

Reviewer 1 Advance Summary and Potential Significance to Field:
The manuscript describes the phenotpyic consequences of mutations in rasip and radilb in zebrafish embryos. The data provide a carefully executed description of lack of Rasip and on junctional integrity at the ISV level, and suggest a number of ways how the respective proteins could exert their effect. Through comparison with ccm1 and heg1 morphants , which exhibit similar phenotypes, a case is made for a possible interaction of these proteins. The study confirms previous work in the mouse (Barry et al 2016) about the importance of Rasip, and will be of interest to readers in the field of vessel morphogenesis and junctional integrity.

Reviewer 1 Comments for the Author:
(1) the abstract reads a little choppy, which is in part due to the fact that not all of the experimental lines are logically connected among each other.
We agree with the reviewer. We think we have improved the flow and coherence of the abstract.

© 2021. Published by The Company of Biologists under the terms of the Creative Commons Attribution License (https://creativecommons.org/licenses/by/4.0/).

(2) The rasip mutants develop early edema, exhibit severe brain hemorrhaging, and show a phenotpye in ISV sprouting that is most prominent early, but appears to sort itself out over time (72hpf). Some of these phenotypic features raise the issue of systemic effects, and whether mutants suffer from a general delay. Cell transplantation of mutants cells into wild type vessels would demonstrate a cell autonomous effect of lack of Rasip, and at least partially adress the issue of a possible systemic effect in rasip mutants.

This point is well taken. We have done two types experiments to address the issue about systemic effects. First, we have performed transplantation experiments, in which we transplanted wild-type cells into mutant embryos, to test whether wild-type cells can behave normally in a mutant environment. Secondly, we performed transient rescue experiments, in which we expressed Rasip1 via an endothelial specific (fli1a) promoter in mutant embryos. We evaluated the phenotype by in vivo time-lapse imaging and immunofluorescent focusing on endothelial junctions, visualized by Pecam-EGFP and VE-cadherin. Taken together, both types of experiments show that autonomous *rasip1* expression is sufficient for normal EC behavior.

These experiments are presented in Figure 3.

(3) rasip mutants show a dramatic hemorrhaging phenotype, but the authors restrain themselves to one panel in figure 1C. Have the authors checked whether the same junctional defects described in the trunk are causative for the brain vasculature defects?
We did not study the cranial vasculature in detail, because our primary objective was to study Rasip1 function in tube formation during angiogenesis and anastomosis, and focused on the blood vessels in the trunk. From past practical experience we know that the midbrain vasculature is prone to hemorrhages. We have occasionally seen this in fli1a:EGFP^{y1} embryos that were wild-type otherwise. Nevertheless, we now have taken a closer look at the midbrain vasculature and found that in principle the junctional organization within the vessels appears normal. The increased incidence of hemorrhages is consistent with weakened endothelial cell junctions.
More importantly, we also saw that the dorsal mesencephalic vascular network (i.e. the mesencephalic central arteries; MMCtAs) is disrupted in some of these embryos, which is likely to be the primary cause for the hemorrhages.

The new images of the midbrain vasculature can be found in S-Figure 1.

(4) rasip mutants are reported to exhibit reduced blood flow - has this been quantified, and does this get fully restored at 72hpf?

In general, there is only a mild reduction in blood flow in the dorsal aorta at 48 hpf (see Figure below). Quantification of blood flow at 72 hpf is difficult because of its vast increase. We agree that hemodynamics play an important role in vascular morphogenesis and homeostasis. However, because of the mild defect and since our study is centered around morphogenetic events occurring up to 48 hpf, we feel that a detailed analysis of hemodynamics would be distracting and prefer to not include blood flow data in the manuscript.

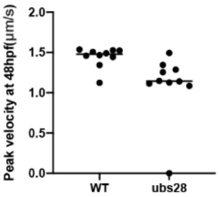

(5) rasip mutants show reduced endothelial cell proliferation at the level of the dorsal aorta - can the authors speculate how this is brought about? In the discussion the obvious is stated ('fewer cells reflect a reduced rate of cell proliferation'), but could this be an effect of altered lumen formation or reduced blood flow as well?
Beyond stating the obvious, the point we try to make is that although proliferation is reduced in ISV this effect is partly compensated by increased recruitment of ECs from the DA. The reduced diameter of the DA is consistent with a reduced number of ECs. We have performed EdU incorporation assays and find a mild reduction in EC proliferation in the DA (see Figure below).

© 2021. Published by The Company of Biologists under the terms of the Creative Commons Attribution License (https://creativecommons.org/licenses/by/4.0/).

However, this effect appears not to be statistically significant. We therefore prefer to exclude these data from the manuscript.

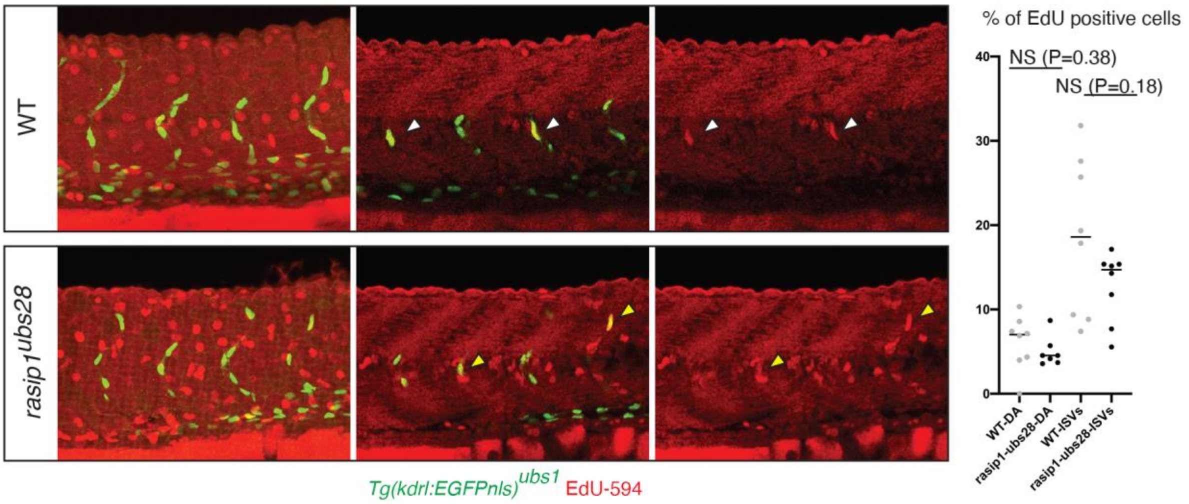

(6) Rasip function has been analyzed previously. While the data presented in figs 2,3,5,6,7 of the present manuscript are certainly interesting, they do not necessarily provide a step advance in our understanding of junction formation and junction stability, or of developmental angiogenesis. One understands better the effects of Rasip loss of function in trunk vessels, but the scope of the work appears somewhat narrow. Data on radil-b and heg1/ccm1 add little to the main story line of the manuscript.

In the current manuscript, we have added transplantation and rescue data showing autonomous and endothelial specific requirement of *rasip1* in different aspects of blood vessel formation. We also provide additional data showing mislocalization of Rasip1 in *heg1* and *ccm1* morphants.

We are aware that this study is for the most part a phenotypic description of the *rasip1* mutant. We describe hitherto unknown roles of Rasip1 in lumen formation and junctional stabilization during sprout outgrowth. The latter function also demonstrates the importance of tethering by tri-cellular junctions as a cellular mechanism to achieve multicellular tube formation. All these phenomena deserve a more in-depth analysis, but they are well out of the scope of this study, where we try to emphasize the wide range of morphogenetic processes where Rasip1 plays an essential role.
We will address these functions of Rasip1 in future studies in detail.

Reviewer 2 Advance Summary and Potential Significance to Field:
The study from Lee et al reports the generation and characterization of vascular morphogenesis in Rasip1 and Radil-b loss of function zebrafish. A previous study using mouse models has identified that Rasip1 is essential for lumen formation (Barry et. al. 2016). This mouse study uncovered an underlying mechanism whereby Rasip1 prior to lumenisation is essential to clear adhesion sites and allow for the initial single lumen to arise between neighboring endothelial cells. Later during lumen formation, Rasip1 also permits for a certain level of actomyosin relaxation (by inhibiting RhoA) allowing lumen expansion.

Here, a zebrafish radil1 mutant model has been used to study endothelial cell behavior along with endothelial junction dynamics by high quality live imaging. In addition, the authors have carefully analysed other morphogenesis events that are driven by cellular rearrangements and essential for the establishment of a functional vascular network, like ISV formation and anastomosis. The results indicate that Rasip1 supports cellular rearrangements in the ISVs and is also essential for clearance of adhesion molecules during the initial stages of anastomosis. This extensive functional analysis of Rasip1 is complemented by the first in vivo analysis of Radil function in the endothelium. Radil is a close orthologue of Rasip1 and has been shown to function redundantly in cultured ECs. Importantly, the work described here reveals overlapping but also distinctive roles for Rasip1 and Radil during vessel network formation.

Reviewer 2 Comments for the Author:

© 2021. Published by The Company of Biologists under the terms of the Creative Commons Attribution License (https://creativecommons.org/licenses/by/4.0/).

While there are some issues that need to be addressed (see below), the study should be well suited for publication.

1. In Figure 1 it appears that there is a higher abundance of ve-cad in the rasip1 mutant embryos. Rasip1 has also been shown to clear adhesion molecules during mouse lumen formation. A similar phenomenon was observed here in zebrafish whereby Rasip1 is essential to clear adhesion molecules during anastomosis. Together this might suggest that Rasip1 more generally is required for ve-cad recycling which in turn would also lead to the compromised cell dynamics identified in the ISVs. Have the authors considered this and examined the differences in ve- cad levels more broadly?

The reviewer raises several important points. It would be interesting to know whether and how VE-cad protein levels are changed in *rasip1* mutants. However, measuring differences in protein levels in the embryo by immunofluorescence or reporter gene expression is not trivial. In the case of immunofluorescence, we see quite some embryo-to-embryo variability in the signal. This is a technical problem. Therefore, protein quantification requires an internal control to differentiate relative levels.
Because, VE-cad is specifically localized at junction this differentiation is not possible. With respect to reporter genes, we noticed a degree of mosaicism (cell-to- cell variation), which makes quantification unreliable and hence translates into prohibitively large sample size for this analysis.

2. It is intriguing to note that there are transient defects in cardiac function and thus blood circulation. Do the authors have an idea why a certain percentage of the mutants can survive whilst others do not?

The short answer is no. Although all *rasip1* mutant embryos exhibit vascular defects, there is varying penetrance of the mutant phenotype among vessels and embryos.
The fact that we see a certain number of “escapers” may be a result of this variation rather than particular defects, which are permissive or non-permissive with respect to development. Nevertheless, *rasip1* expression (i.e. down-regulation in the DA upon blood flow initiation) and mutant phenotypes suggest that Rasip1 is important for blood vessel formation and dispensable during late development and adulthood. This is in agreement with mouse data, which showed that late deletion of *Rasip1* does not interfere with adult life (Koo et al., Angiogenesis (2016)).

Interestingly, the vasculature in radilb mutants is described as being less severely affected and that in these embryos also flow is less compromised. Taking into account these observations, how does the reduction in flow pressure actually contribute to some of the earlier phenotypes that have been described? For example, lumenisation of the ISVs is initiated at 4 dpf but this is also when circulation is described to recover. Do the vessels in the head also lumenise at this later stage? Do other phenotypes like the cellular rearrangements in the ISVs and ISV detachment also recover? How much does flow impact on anastomosis and clearance of adhesion proteins during this process?Can the authors compare their results to a scenario whereby flow is temporarily reduced or lost?

In our manuscript we describe that the *radilb* mutant phenotype is weaker than the *rasip1* mutant phenotype, whereas the double mutants show aggravated defects. We have focussed on the formation of ISVs (primary sprouts), which do not depend on blood flow as has been shown (among others) in *silent heart* and *ve-cad (cdh5)* mutants (e.g. Sauteur et al., Cell Rep. (2014)). Alterations of blood flow are therefore unlikely to modify the *rasip1* or *radilb* mutant during ISV sprouting (24-28 hpf). In a similar way, anastomosis during DLAV formation takes place in the absence in blood flow in both wild-type and *rasip1* mutants.

It would be interesting to examine the capacity of the vessel network to reconnect and recover by doing a temporal analysis of a viable radil1 mutant. Embryo matched images taken at 2days and 6-7days would reveal if a compromised network re- establishes or whether the embryos are simply viable whilst lacking
several ISVs.

If we understand correctly, the reviewer is asking to test how ISVs recover in *radilb* mutants over time - similar to the experiments presented in Figure 4B for *rasip1* mutants. In our study we have

© 2021. Published by The Company of Biologists under the terms of the Creative Commons Attribution License (https://creativecommons.org/licenses/by/4.0/).

compared the phenotypes of *radilb* and rasip1 mutants. *radilb* phenotypes are similar to those of *rasip1* mutants, but weaker. We have not gone into more depth of the *radilb* phenotype, because we feel it would unnecessarily distract from our analysis of the role of Rasip1 during blood vessel formation.

3. The authors show that there are additional cells from the dorsal aorta that migrate into the ISVs to compensate for reduced cell division during sprouting angiogenesis. Does this phenomenon have an effect on dorsal aorta integrity and cell number?

The reviewer is correct. Although we observe a small (albeit non-significant) reduction of EC proliferation in the dorsal aorta (see answer to point (5) of reviewer #1), we see a small but significant reduction of DA diameter in *rasip1* mutants, as we show in supplemental Figure 2B (see below, on the left). Although, we noticed some change in endothelial cell shape in the dorsal aorta (i.e. less elongated ECs - see below, but not included in the manuscript), we consider the change in diameter a likely consequence in reduced cell number. Cell shape regulation is a very important aspect of morphogenesis and it will be interesting to examine the role of Rasip1 in this process. However, this will require elaborate 3-D modelling and analysis of hemodynamic forces, which is outside the scope of this paper.

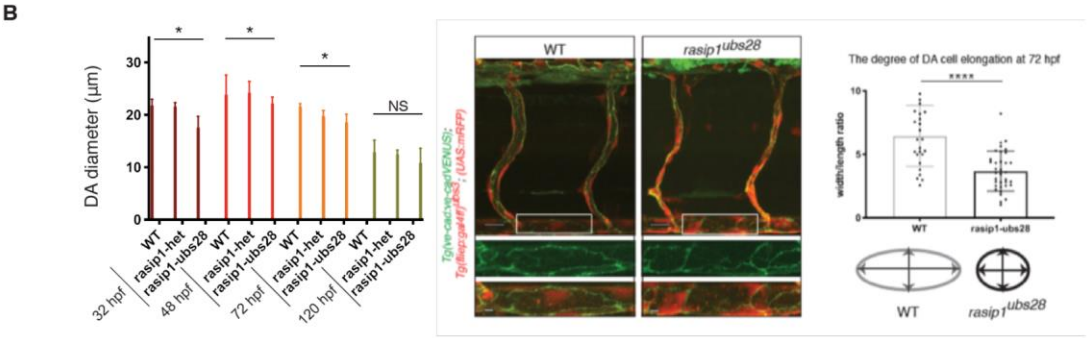

4. The authors further explore the relevance of Heg in Rasip1 function. Heg is proposed to recruit Rasip1. Have the authors examined whether Rasip1 is indeed mis-localised in heg morphants? This would really support the idea that heg loss of function could produce a phenocopy of rasip1 vascular defects.

We have followed up on the recommendation of the reviewer and analyzed Rasip1 localization by immunofluorescence in *heg1* and *ccm1* mutants. Because of the variability in immunofluorescent signals, we have carefully quantified Rasip1 signals at junctions and put them in relation to the signal at the nearby apical membrane.
Quantification of relative signal strength at cell junction/apical membrane shows a reduction of Rasip1 at EC junctions in *heg1* and *ccm1* morphants (n>50). The new data are presented in Figure 8D and E (see below).

© 2021. Published by The Company of Biologists under the terms of the Creative Commons Attribution License (https://creativecommons.org/licenses/by/4.0/).

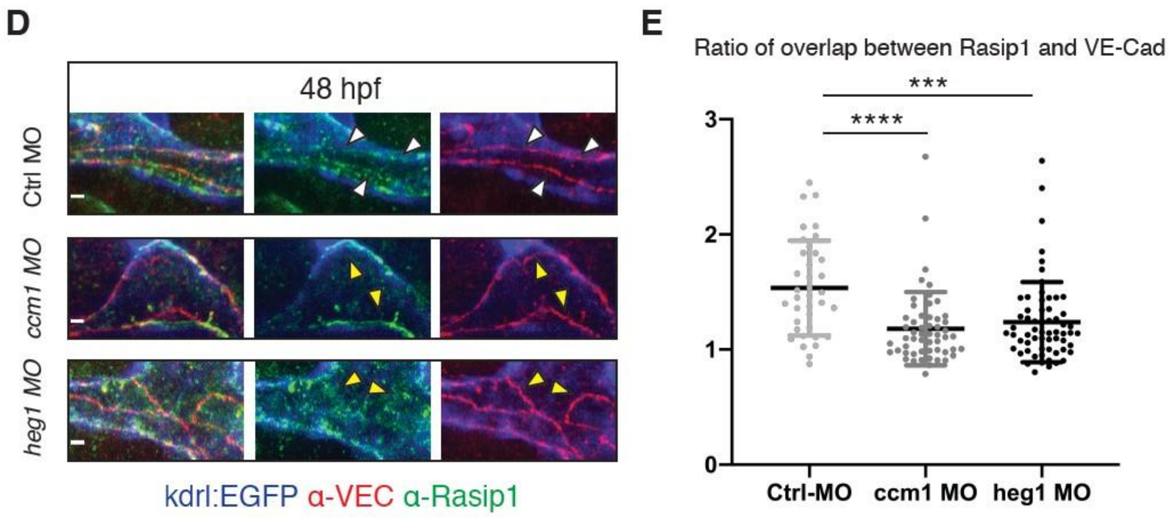

Minor:

1. There is no reference to the movie numbers in the main text and the movies also do not have a descriptive name so it is not clear to figure out precisely which movie is being discussed in the main text. This information can only be found in the Supplementary movie legends.

We have now referenced the movies also in the main text.

2. From the temporal analysis of Rasip1 protein expression in Sup Fig 4 the authors propose that Rasip1 becomes downregulated later during development and therefore might not be essential for vessel maintenance. It appears however that Rasip1 during the investigated timecourse becomes more localised/ junctional? This does not necessarily mean that it is less relevant at the later stages. Perhaps the authors can rephrase these conclusions.

With respect to Rasip1 expression and localization during blood vessel formation we observe three phases (1) apical (2) enriched at junctions and (3) overall downregulation. Our interpretation that Rasip1 may be less relevant in more mature vessels, is exemplified by the blood carrying dorsal aorta in supplemental Figure 5C. Our interpretation is in agreement with mouse data showing that Rasip 1 is non- essential in adult mice (Koo et al., Angiogenesis (2016)) and our own observation that *rasip1* mutants can develop to fertility.

3. At 24hpf (Sup Fig 4a) there should already be sprouts that have migrated halfway the trunk and at 28hpf these sprouts would have reached the top (Sup Fig 4b). Presumably the authors have mis-labeled the images represented here. The authors should correct the text and the labelling of this figure to the representative developmental time points.

We thank the reviewer for this observation. In our hands, ISV sprouting commences at around 24 hpf in the region of the yolk extension. However, it should be noted that the supplemental Figure 4 (now S-Figure 5!) shows Rasip1 and ZO1staining in order to show Rasip1 localization relative to EC junctions. Therefore, the front of the sprout (i.e. the tip cell) is not labelled and the sprout appears developmentally younger than it actually is. However, the panel B is clearly at 26 hpf. This has been fixed.

4. In Figure 6 the authors indicate that Rasip1 does not co-localise with VE-cadherin at initial contacts that lead up to anastomosis. However, since this is fixed tissue it can not be concluded that this VE-cadherin positive contact is the stable contact that would go on to anastomose. Would it be possible that Rasip1 could localise to the actual stable contacts that go on to anastomose?

We have worked on the subject of vascular anastomosis for many years. In a previous paper, we have quantified the number of VE-cadherin mediated contacts that are needed to initiate definitive anastomosis – usually one or two (Sauteur et al., Development (2017)). Thus, we are

© 2021. Published by The Company of Biologists under the terms of the Creative Commons Attribution License (https://creativecommons.org/licenses/by/4.0/).

quite confident that the description of the contacts in Figure 6 is accurate, especially since stable contacts are established already during filopodial contacts – prior to the stages shown in Figure 6.

Reviewer 3 Advance Summary and Potential Significance to Field:
The manuscript of Lee at al., describes the blood vascular defects associate with the loss of function of Rasip1 (Ras-interacting protein 1), an endothelial- specific regulator of junctional dynamics in blood vessel formation. The authors report morphological defects in both the brain and the trunk vessels. Using static and live imaging they report that Rasip1 is required for tricellular junctions during the formation of ISV. Rasip1 cooperates only partially with other proteins in multimeric complexes consisting of Rap1, Rasip1, Radil. Based oh phenotype similarity Rasip1 seems to interact with Ccm1 and Heg 1 during anastomosis formation.

The imaging work presented in this manuscript is incredibly detailed and beautifully represented as it is typical for Dr. Belting work. My comments are listed below. Overall the conceptual organization of the data makes the manuscript difficult to follow. For example, the initial description of some of the catastrophic brain and aorta diameter defects makes the data presented afterward difficult to interpret as direct effect of Rasip1 loss of function. For example, how much of ISV and anastomosis defect consequence from a secondary lack of aorta lumen formation, blood flow or hemorrhages?

Reviewer 3 Comments for the Author: More in detail:

1) I would suggest moving the figure 1 as supplemental information as these phenotypes were previously reported and the generation of the mutant can be described in the methods.
We agree. This has been changed.

2) It is interesting that Rasip1-/- defects are transient. Do the author work with MZ embryos? Can the author study or discuss of why this is the case? For example, do they think that Rasip1 is only required for early vascular development and other mechanism will be in place later on? Is this phenomenon the result of genetics compensation or more interestingly Rasip1-/- are reveling new molecule(s) that function similarly?

Yes, we work with mz-embryos. We did not notice any differences between homozygous embryos derived from homozygous mothers or a heterozygous incross, indicating no maternal contribution. In support of this, lack of maternal contribution, the expression levels of rasip1 are very low/absent during early development (see RNA-seq data obtained from White RJ, et al. Elife. 2017 16;6:e30860. in the figure below).

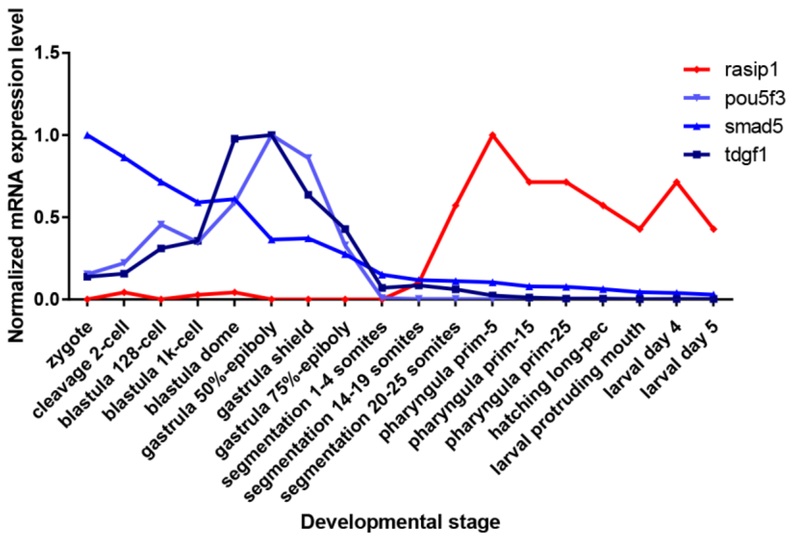

We did not explore the possibility of genetic compensation for three reasons. (1) Our ubs28 allele is a large deletion (>30kb) ablating most of the open reading frame. (2) The mutant phenotype is stronger than a previously published ATG-morpholino knockdown phenotype (Wilson et al., Blood (2013)). (3) The overall mutant phenotype is consistent with previously published mouse mutants.

© 2021. Published by The Company of Biologists under the terms of the Creative Commons Attribution License (https://creativecommons.org/licenses/by/4.0/).

Our interpretation is that Rasip1 is mostly required during morphogenetic stages of vascular development, when the EC junctions and the actin cytoskeleton are extremely dynamic. Upon initiation of blood flow and later stages of development, EC junctions become less dynamic and more stable as nicely shown by Lagendijk et al. (2017). Furthermore, additional stabilizing factors may be at work, e.g. mural cells, EC-ECM interaction etc.

3) It is unclear how the author defines the mutant, some time is called rasip1ubs28 and some other Rasip1 mut. Are these definitions refering to homozygous mutants? Did the author explore the phenotype in the het model? Could it be that the het have less major vascular phenotypes, while still have ISV tube formation defect?

Yes, when we talk about *rasip1* mutants, we mean homozygous *rasip1*^{ubs28}. In the main text we do not use the ubs28 allele designation to improve readability.
In several experiments, we have compared wild-type, heterozygous and homozygous embryos. We did not see any differences between wild-types and heterozygotes.

4) It is difficult to interpret the ISV and anastomosis defect while the mutant has early DA defect. Can the author perform transplantation experiment to assay the formation of ISV by mutant cell in a WT DA?

As requested by the reviewer, we have now also analyzed genetic mosaics. We have performed transplantation experiments, in which we transplanted wild-type cells into mutant embryos, to test whether wild-type cells can behave normally in a mutant environment. Secondly, we performed transient rescue experiments, in which we expressed Rasip1 via an endothelial specific (fli1a) promoter in mutant embryos. We evaluated the phenotype by in vivo time-lapse imaging and immunofluorescent focusing on endothelial junctions, as visualized by Pecam-EGFP andVE-cadherin.
Taken together, both types of experiments show that autonomous *rasip1* expression is sufficient for normal EC behavior.

These new data are presented in Figure 3.

5) Could it be that the proliferation defects maybe the primary cause of the ISV defect? Beside counting the nuclei I would suggest to perform an Edu staining to assay whether endothelial cells are less proliferative in general.

We have performed EdU incorporation experiments, which show a very minor, non- significant reduction in EC proliferation (data shown in response (Q5) to reviewer 1). Apparently, the EdU assay is not very sensitive in picking up small changes in proliferation. In the context of ISV sprouting, following individual nuclei appears to be the more robust assay.

The cell pairing defects cannot be explained by reduced cell proliferation. We have previously shown that reduced cell number does not prevent cell rearrangements and multicellular tube formation (Angulo-Urarte et al., Nat. Comm (2018)). We clearly identify dissociation of tri-cellular junctions as the underlying cause of pairing defects.

6) Is the function of Rasip1 is the ISV lumen formation different from what expected based on the previous studies e.g Barry et al., 2016. It is difficult to point out from the paper what is new about the authors claim.

In our study, we focus on angiogenic sprouting and anastomosis, whereas Barry et al. (2016) have described the role of Rasip1 during vasculogenesis. Nevertheless, the junctional clearing defect during anastomosis is essentially the same as described by Barry during DA formation – highlighting that lumen formation in both systems share similar cellular and molecular mechanisms. In addition, we observe the formation of intracellular lumens, which has not been described by Barry et al.. However, the most striking (and novel) defect we observe relates to the stabilization of tri-cellular junctions. Loss of Rasip1 leads to a dissociation of tri-cellular junctions, which in turn prevents proper cell rearrangements and multicellular tube formation (cord hollowing). We suggest that most of the lumen formation defects in ISV are caused by this rearrangement defect.

© 2021. Published by The Company of Biologists under the terms of the Creative Commons Attribution License (https://creativecommons.org/licenses/by/4.0/).

7) Interestingly the VE-cad-Venus has a different intensity between WT and Rasip1-/- (Fig. 2a). Is this representative of the results? Do the mutant have more vecad expression or larger adherent junctions?

This question is difficult to address. The expression of the cdh5:cdh5-TENS transgene can be quite variable. It is difficult to quantify expression levels over several embryos, when lacking an internal control. We have thought of taking a proteomic approach, but VE-cad is difficult to detect in our samples, because of low abundance in the embryo. At this moment, we prefer to not address this issue in the current manuscript.

8) Can some of the defects observed in the ISV explained by the lack of flow? Can the author rescue this phenotype by expressing Rasip1in endothelial cells? The hemodynamics defect likely plays a role in both lumen formation and maintenance. It is critical to distinguish whether these defects are the direct result of lacking Rasip1 or a combination of the two.

Blood flow is an important regulator of angiogenesis. Primary ISV sprouting and anastomosis, however, occur independently of blood flow, as determined by analysis of *silent heart* embryos (e.g. Herwig et al., *Current Biol.* (2011)). Especially anastomosis at the level of the DLAV is similar to lumen formation in the dorsal aorta, which also occurs prior to blood flow. However, we see blood flow defects in the ISVs later on (Figure 4). These blood flow defects are in agreement with the cell pairing defects in *rasip1* mutants, which prevent multicellular tube formation. In support of this, transplanted wild-type cells exhibit normal junctional remodeling in the mutant background. Furthermore, transient mosaic expression of Rasip1 in ECs is sufficient to rescue ISV development. These findings strongly argue that abnormal blood flow is not the cause of the vascular defects observed in *rasip1* mutants.

9) Figure 6 showed the Rasip1 endogenous protein expression within the ISV. Can the author provide a quantification to prove the expression or localization changes that they clame?

We describe the distribution of Rasip1 protein relative to the VE-cad. We deem the junctional/non-junctional localization presented in Figure 6 very obvious; upon anastomosis and junctional ring formation, Rasip1 localisation in the apical patch is seen in all mutant embryos analysed. We have, however, quantified the relative Rasip1 localization in the context of *heg1* and ccm1 knock-down.

10) None of the data provide in figure 7 are quantified. It is difficult to conclude that the effects of Rasip1 and Radil-b mutant alone or in combination are significant.
Quantifications are required in all the genotypes for each ISV and anastomosis defect to further conclude the cooperation between Rasip1 and Radil-b.

We have now included quantifications for junctional clearing and presence of blood flow in the different genotype combinations in Figure 7.

11) It is difficult to conclude or even suggest that Rasip1 might cooperate with Heg1 and CCM1 based solely on the comparison between phenotype. More detail epistasis and possibly more molecular experiments are needed to prove this hypothesis.
A cooperation between Rasip1, Heg1 and Ccm1 has been proposed before (This has been reviewed by Lampugnani et al. CSH Persp. Biol. (2017)). Here we show that *heg1* and *ccm1* knockdown mimics *rasip1* mutants, which supports this hypothesis. In particular we now include novel data showing cell pairing defects in *heg1/ccm1* morphants. We further substantiate these interactions with additional data showing mislocalization of Rasip1 in *heg1* and *ccm1* morphants. These data are presented in Figure 8.

<u>Second decision letter</u>

MS ID#: DEVELOP/2020/197509

© 2021. Published by The Company of Biologists under the terms of the Creative Commons Attribution License (https://creativecommons.org/licenses/by/4.0/).

MS TITLE: Control of dynamic cell behaviors during angiogenesis and anastomosis by Rasip 1

AUTHORS: Minkyoung Lee, Charles Betz, Jianmin Yin, Ilkka Paatero, Niels C Schellinx, Adam N Carte, Christopher W Wilson, Weilan Ye, Markus Affolter, and Heinz-Georg Belting
ARTICLE TYPE: Research Article

I am happy to tell you that the referees are happy with your revisions and your manuscript has been accepted for publication in Development, pending our standard ethics checks.

Reviewer 1

Advance summary and potential significance to field

See previous round of revision.

Comments for the author

The authors have added additional information and experimental evidence, which adresses most of my and teh other reviewers' comments.

Reviewer 2

Advance summary and potential significance to field

In the revised manuscript by Lee et al. the authors have positively addressed concerns that were raised. They included a range of new experiments and more extensive quantification of data.

Efforts made to examine Rasip1 function cell-autonomously, by using cell transplantation, are especially noteworthy since these experiments can be extremely time consuming and likely would have not been easy to coordinate whilst still dealing with COVID restrictions. The results show that wildtype endothelial cells can assemble ISVs normally in a rasip1 mutant environment and that thus it is unlikely that rasip1 deficiency outside the endothelium is what causes the vascular defects. The reciprocal experiment; rasip1 mutant endothelial cells in wildtype animals would have really supported cell-autonomy but I can appreciate that this might have been difficult to achieve considering time and pandemic restraints.

Also interesting is the new data on the aorta phenotype, showing less cells and an elongation phenotype which might lead into further studies.

Quantifications of Rasip1 expression in heg1 and ccm1 morphants supports the proposed mechanism and strengthens that aspect of this story.

Comments for the author

I strongly support publication of this revised work in Development.

© 2021. Published by The Company of Biologists under the terms of the Creative Commons Attribution License (https://creativecommons.org/licenses/by/4.0/).
